# Unexpected structural complexity of *d*-block metallosupramolecular architectures within the benzimidazole-phenoxo ligand scaffold for crystal engineering aspects

**DOI:** 10.1038/s41598-023-45109-z

**Published:** 2023-10-23

**Authors:** Dawid Marcinkowski, Maciej Kubicki, Giuseppe Consiglio, Zbigniew Hnatejko, Anna M. Majcher-Fitas, Robert Podgajny, Violetta Patroniak, Adam Gorczyński

**Affiliations:** 1 Faculty of Chemistry, Adam Mickiewicz University, Uniwersytetu Poznańskiego 8, 61-614 Poznań, Poland; 2 Dipartimento di Scienze Chimiche, Università di Catania, 95125 Catania, Italy; 3 Faculty of Physics, Astronomy and Applied Computer Science, Jagiellonian University, Łojasiewicza 11, 30-348 Kraków, Poland; 4 Faculty of Chemistry, Jagiellonian University, Gronostajowa 2, 30-387 Krakow, Poland

**Keywords:** Ligands, Molecular self-assembly, Crystal engineering

## Abstract

Design of metallosupramolecular materials encompassing more than one kind of supramolecular interaction can become deceptive, but it is necessary to better understand the concept of the controlled formation of supramolecular systems. Herein, we show the structural diversity of the bis-compartmental phenoxo-benzimidazole ligand H_3_**L**^**1**^ upon self-assembly with variety of *d*-block metal ions, accounting for factors such as: counterions, pH, solvent and reaction conditions. Solid-state and solution studies show that the parent ligand can accommodate different forms, related to (de)protonation and proton-transfer, resulting in the formation of mono-, bi- or tetrametallic architectures, which was also confirmed with control studies on the new mono-compartmental phenoxo-benzimidazole H_2_**L**^2^ ligand analogue. For the chosen architectures, structural variables such as porous character, magnetic behaviour or luminescence studies were studied to demonstrate how the form of H_3_**L**^**1**^ ligand affects the final form of the supramolecular architecture and observed properties. Such complex structural variations within the benzimidazole-phenoxo-type ligand have been demonstrated for the first time and this proof-of-concept can be used to integrate these principles in more sophisticated architectures in the future, combining both the benzimidazole and phenoxide subunits. Ultimately, those principles could be utilized for targeted manipulation of properties through molecular tectonics and crystal engineering aspects.

## Introduction

Nature can harness weak, non-covalent interactions to achieve highly complex architectures, ultimately being responsible for observed forms of Life that we know of^1–3^. Supramolecular chemistry takes inspiration from there in an attempt to demonstrate at least similar level of control and complexity, which could lead to materials formed via design, ultimately in a pre-programmed and controlled manner^4–7^. The information encoded within the molecular building blocks can lead to their self-assembly, which can be accomplished through implementation of the following approaches: (i) hydrogen-bonding motifs; (ii) metal–ligand coordination bonding; (iii) other non-covalent interactions e.g. electrostatic, van der Waals electrostatic and π-interactions^8–10^. Methods (i) and (ii) provide a high degree of directionality and therefore numerous architectures can be formed of varying dimensionality, specifically including cages^8,11,12^, metal–organic-frameworks (MOF)^13–15^, covalent-organic-frameworks (COF)^16–19^, HOFs^20–22^ and polymers^23–27^. Controlled formation of such architectures can however become challenging when more than one supramolecular interaction is taken under consideration.

We have turned our attention to the hydrazone ligands, since their flexibility and structural tunability, followed by facile synthesis can lead to multifunctional metallosupramolecular architectures^28–31^. These include e.g. photoresponsiveness^32,33^, stimuli-reponsive magnetism^29,34^, biological properties^35,36^, sensing, preparative organic chemistry^37^ and so on^33^. Whereas the hydrazone counterpart can solely serve as the connector of molecular tectons, we became specifically interested in the structure/properties relations of the benzimidazole (‘benz’)^38^ and phenoxide (‘phenox’) based architectures. The former ones were particularly recognized as influencing the biological activity and thus potential pharmaceutical applications^39–44^. The latter ones are effective chelating units towards various coordination architectures, important from the point of view of molecular tectonics.

**Figure 1:**
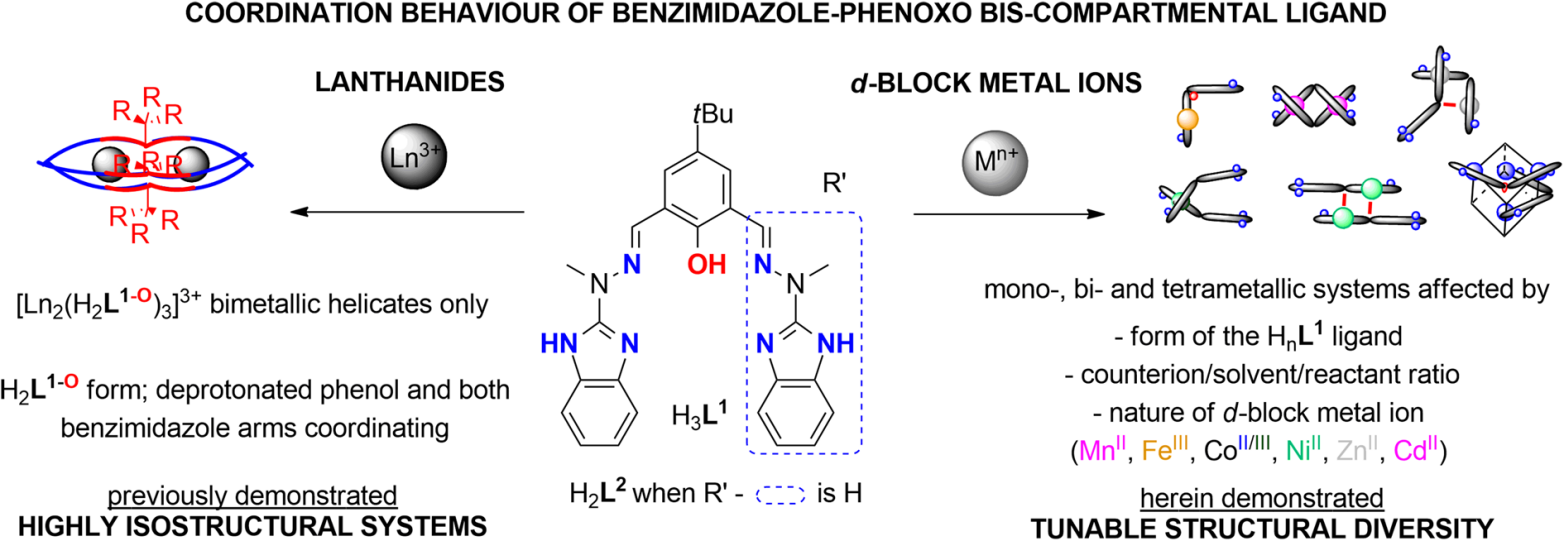
Coordination behaviour of bis-compartmental phenoxo-benzimidazole ligand H_3_**L**^**1**^ in the presence of lanthanides^49^ and *d*-block metal ions.

**Figure 2:**
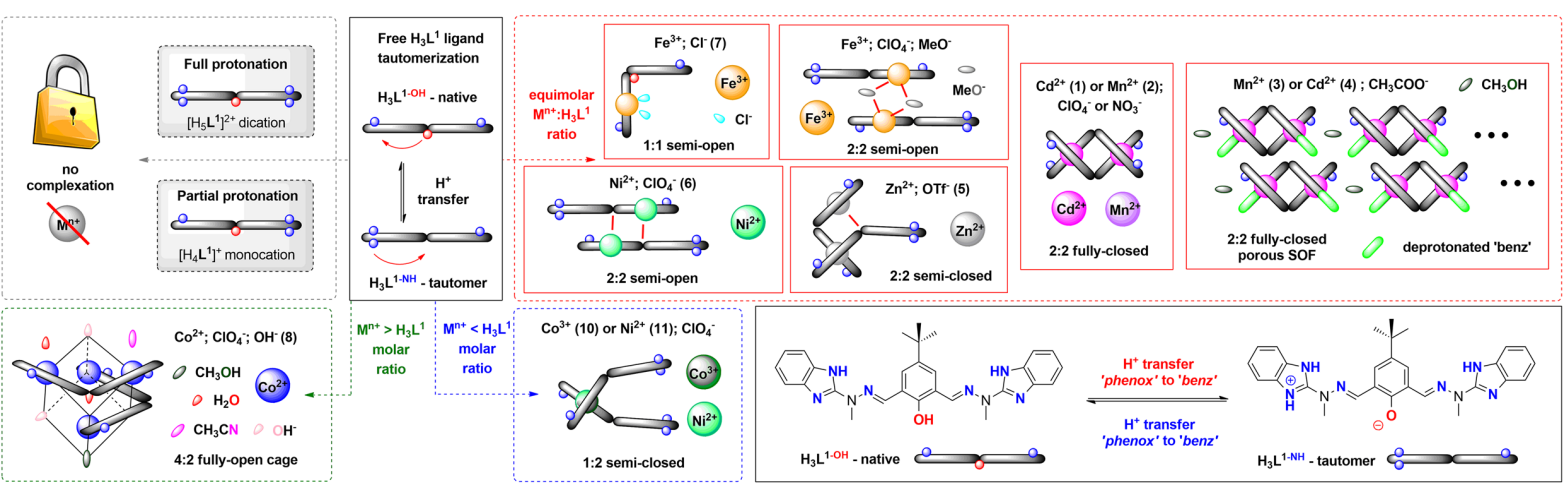
Schematic representation of ligand H_3_**L**^**1**^, its varying (de)protonation modes and associated molecular architectures. Semi-open (**S**–**O**); semi-closed (**S–C**), fully-open (**F–O**) and fully-closed (**F–C**) nomenclature is associated with the ligand conformations and associated binding of the metal ion. Molecular formulas and in-depth discussion is presented in Fig. 3 (**F–C**), Fig. 4 (**S–O** and **S–C**) and Fig. 5 (**F–O** and **S–C**). Coloured oval marks stand for auxiliary monodentate ligands, whereas ‘phenox’ and ‘benz’ phrases stand for the phenoxide and benzimidazole moieties of the ligand scaffold, respectively.

Our previous experience demonstrates that even subtle structural alterations such as topology of the non-coordinating (benz)imidazole group can be responsible for significant changes in the catalytic processes of silanes with unsaturated organic compounds^45–47^, as well as for the biological outcomes in terms of interactions with nucleic acids^48^. When ‘benz’ is combined with ‘phenox’ in a bis-compartmental ligand H_3_**L**^**1**^, we were able to demonstrate how lanthanide(III) assemblies can help in better understanding of molecular nanomagnetism and the underlying symmetry aspects^49,50^. When combined with Mn^II^ or Fe^III^, bimetallic assemblies were constructed that function as selective sensors for detection of neutrotransmitters^51,52^. Importantly, we did observe highly isostructural character of synthesized Ln^III^ helicates^49^, conversely to the *d*-block metal ions where tunability of structure was hinted on (Fig. 1). We therefore decided to pursue the extent to which the architectures with *d*-block metal ions can be modulated herein, thus leading to unexpected structural diversity for such a deceptively simple bis-compartmental ligand H_3_**L**^**1**^. We herein provide a multilevel approach for modulation of varying coordination architectures via (i) different *d*-block metal ions; (ii) counterion and experimental conditions (Fig. 2). We also rationalize our understanding of the solid state/solution structural features and how it translates to the observed luminescence and magnetic properties.

## Results and discussion

Ligand H_3_**L**^**1**^, thanks to its conformational flexibility and possibility of different protonation states, allows for the synthesis of a variety of *d*-block metal ions complexes (Fig. 2), even though it uses only three coordination centers: two ring nitrogen atoms from terminal benzimidazole ring and the phenolic oxygen atom. When acid/base character of these groups is compared, 7 forms of ligand can be envisaged (Scheme S3), out of which 5 are observed in the solid state (see Sect. “Solid state structural description of synthesized architectures”): diprotonated [H_5_**L**^**1**^]^2+^, neutral H_3_**L**^**1–OH**^ and H_3_**L**^**1–NH**^ as well as mono- [H_2_**L**^**1–O**^]^–^ and bis-deprotonated [H**L**^**1**^]^2**–**^. Please note that when acidity (pKa) constants are compared, phenol (ca. 10)^53^ is easier deprotonated than benzimidazole (ca. 12.8)^54^. But the relatively small differences allow both forms H_3_**L**^**1-OH**^ and H_3_**L**^**1-NH**^ to be observed under synthetic reaction conditions, with basic character of the ‘benz’ promoting the H^+^ transfer. Such appears to be strongly dependent on the nature of the metal ion^55^. The existence of monoprotonated [H_4_**L**^**1**^]^+^ was established through the UV–Vis titrations (Fig. S10) and happens at the benzimidazole nitrogen atoms. The protonated benzimidazole arms were often observed upon the proton transfer from phenol to benzimidazole (see semi-closed **S-C** and semi-open **S–O** structures in Sect. “Solid state structural description of synthesized architectures”).

Among the coordination compounds studied here, we group them according to the general molar ratio of the metal ion M^n+^ and H_3_**L**^**1**^ ligand, since the observed structural diversity can then be presented in a more organized manner. The basic coordination algorithms allow for M^n+^:H_3_**L**^**1**^ molar ratio being in the 1:2; 1:1 and 2:1 modes or their subsequent multiplication. When M^n+^  < H_3_**L**^**1**^, an anticipated 1:2 semi-closed **S-C** system is observed with one binding subunit of the ligand occupied, as exemplified by structures (**10**) and (**11**) with weakly coordinating counterions—here ClO_4_^–^—and Co(III) or Ni(II) metal ions, respectively (Fig. 2–blue arrow). A similar reference compound was prepared with mono-compartmental phenoxo-benzimidazole H_2_**L**^**2**^ ligand analogue with Co(III) ions (**9**). These species are stable under ^1^HNMR solution conditions and give a clear molecular fingerprint of the locked 1:2 (M^n+^:H_m-1_**L**^**n**^) conformation with deprotonated phenoxo-group (see Sect. “Solution NMR studies”). The equimolar ratio (Fig. 2–red arrow) allows to complexate one or two binding subunits of the ligand, leading to the most diverse and unexpected self-assembling examples.

Depending on the nature of the M^n+^ ion and the corresponding counterions, semi-open **S–O** (**7**–Fe^III^/Cl^–^; **6**–Ni/ClO_4_^–^; Fe^III^/ClO_4_^–52^), semi-closed **S–C** (**5**–Zn^II^/OTf^–^) and fully-closed **F–C** (**1**–**4**; Mn(II)/Cd(II)/ClO_4_^–^/NO_3_^–^/AcO^–^) types of complexes are obtained. While only a combination of Fe(III) and Cl^–^ leads to the single occupation of the H_3_**L**^**1**^ ligands NNO binding pocket in a 1:1 (M^n+^:**L**) **S**–**O** manner (**7**), the same **S**–**O** coordination algorithm is observed for Fe(III)^51^ and Ni(II) in the presence of perchlorates but as the 2:2 (M^n+^:**L**) dimeric complexes, respectively. Proton transfer from phenolic oxygen to benzimidazole nitrogen is observed, however the bridging through phenoxo-moiety is established only when Ni(II) ions are employed; with Fe(III) the deprotonated methoxy anions are responsible for the dimerization phenomenon. The initially anticipated 2:2 (M^n+^:**L**) fully-closed **F**–**C** coordination compounds (i.e. with the H_2_**L**^**1-O**^ form; deprotonated phenol and both benzimidazole arms coordinating) were observed only for Mn(II) and Cd(II) metal ions, irrespectively if we chose weakly (ClO_4_^–^) or strongly (NO_3_^–^) coordinating anions. This suggest that such architectural composition is preferred when there are no crystal field stabilization effects, as these are non-existent for d^5^-high spin configuration octahedral ligand field splitting (Mn(II)) and fully-closed d^10^ (Cd(II)) complexes. Very unexpectedly, when acetate anions are used during the synthesis, the 2:2 **F**–**C** architectures become deprotonated at one of the benzimidazole-ligand arms, thus leading to the porous supramolecular organic frameworks (SOF)—(**3–**Cd(II)/AcO^–^) and (**4–**Mn(II)/AcO^–^)—formed through self-organization via the H-bonding-methanol of the above architectures. Each organic ligand exists in the doubly deprotonated form [H**L**^**1**^]^2–^, thus formed complexes are electrostatically neutral and plausibly constitute the limit to the degree of deprotonation of the H_3_**L**^**1**^ ligand. In addition, we were delighted to observe that when Zn(II) as the metal ion and OTf^-^ were used, the 2:2 (M^n+^:H_3_**L**^**1**^) semi-closed **S**–**C** structure was observed, which is the missing link between the 2:2 (M^n+^:**L**) **S**–**O** and the 2:2 (M^n+^:**L**) **F**–**C** molecular compositions. These differences were also observed in the structural solution and luminescent studies (see Sects. “Solution NMR studies” and “Absorption and emission measurements”).

We did not observe the similar structural diversity when M^n+^  > H_3_**L**^**1**^ reaction conditions were ensured. One could increase the yield of the 1:1 (M^n+^:**L**) **S**–**O** complex formed from FeCl_3_ salts, since additional equivalent of Fe(III) ions exists in the form of the [FeCl_4_]^–^ entity as the counterion (**7**). However, we did observe the expected full saturation of the ligand resulting in the 2:1 (M^n+^:H_3_**L**^**1**^) composition with Co(ClO_4_)_2_ salt (Fig. 2–green arrow), which can bind with the adventitious hydroxyl OH^–^ anions, thus leading to the unexpected characterization of the 4:2 cuban-type architecture with the Co_4_O_4_ cube-like core (**8**). Interestingly, when reaction was carried out in a 1:1 (M^n+^:H_3_**L**^**1**^) molar ratio with Co(ClO_4_)_2_ as the coordinating salt, a mixture of crystals was obtained, which constitute both the **F–O** [Co_4_(H_2_**L**^**1–O**^)_2_] cubane (**8**) and the **S**-**C** [Co(H_3_**L**^**1–NH**^)_2_] (**10**) complexes, proving that neither is thermodynamically more preferred and one can tune the final yield of obtained compounds with the molar ratio only.

The ligand was previously presented by us^51^, but due to important impact on crystallographic discussion it is presented in this paper. The coordination of the metal centers in presented complexes is in general equal to 6 and adopted octahedral environment, but there are some interesting exceptions; also the geometry of the ligands differs from case to case. Moreover, the crystal structures of the complexes, besides the counterions, contain various solvent molecules, more or less disordered, which are responsible for relatively high crystallographic R factors. In our opinion however, this does not makes an obstacle against the reasonable discussion of the geometry of the complexes or the general features of the crystal structures.

### Solid state structural description of synthesized architectures

#### Diprotonated form of ligand H_3_L^1^: [H_5_L^1^]^2+^ (ClO_4_^–^)_2_

There are two symmetry-independent cations in the asymmetric part of the unit cell, and they have very similar geometry (Fig. S1). One can however note, that relative orientation of phenolic OH and *tert*-butyl groups are reverse in both molecules (OH hydrogen in each case makes reasonable hydrogen bonds). The cations are almost planar, the dihedral angles between the mean planes of the terminal ring systems are 13.5° and 10.2° (the relevant geometrical features of the ligands are listed in the Table S1 in SI). Intramolecular OH···N hydrogen bonds help in adopting such conformation. In the crystal structure, besides four perchlorate anions, the methanol and (heavily disordered) toluene molecules have been found. The crystal structure results – besides the electrostatic interactions between charged species) – form the extensive network of hydrogen bonds, involving all strong hydrogen bond donors (Fig. S2). The details of the hydrogen bonds are listed in Table S2 in SI.

#### M^n+^: H_3_L^1^ equimolar, fully-closed architectures (the ones with both benzimidazole arms are occupied for coordination—Fig. 3A)

##### [Cd_2_(H_2_L^1–O^)_2_](ClO_4_)_2_; (1)

**Figure 3:**
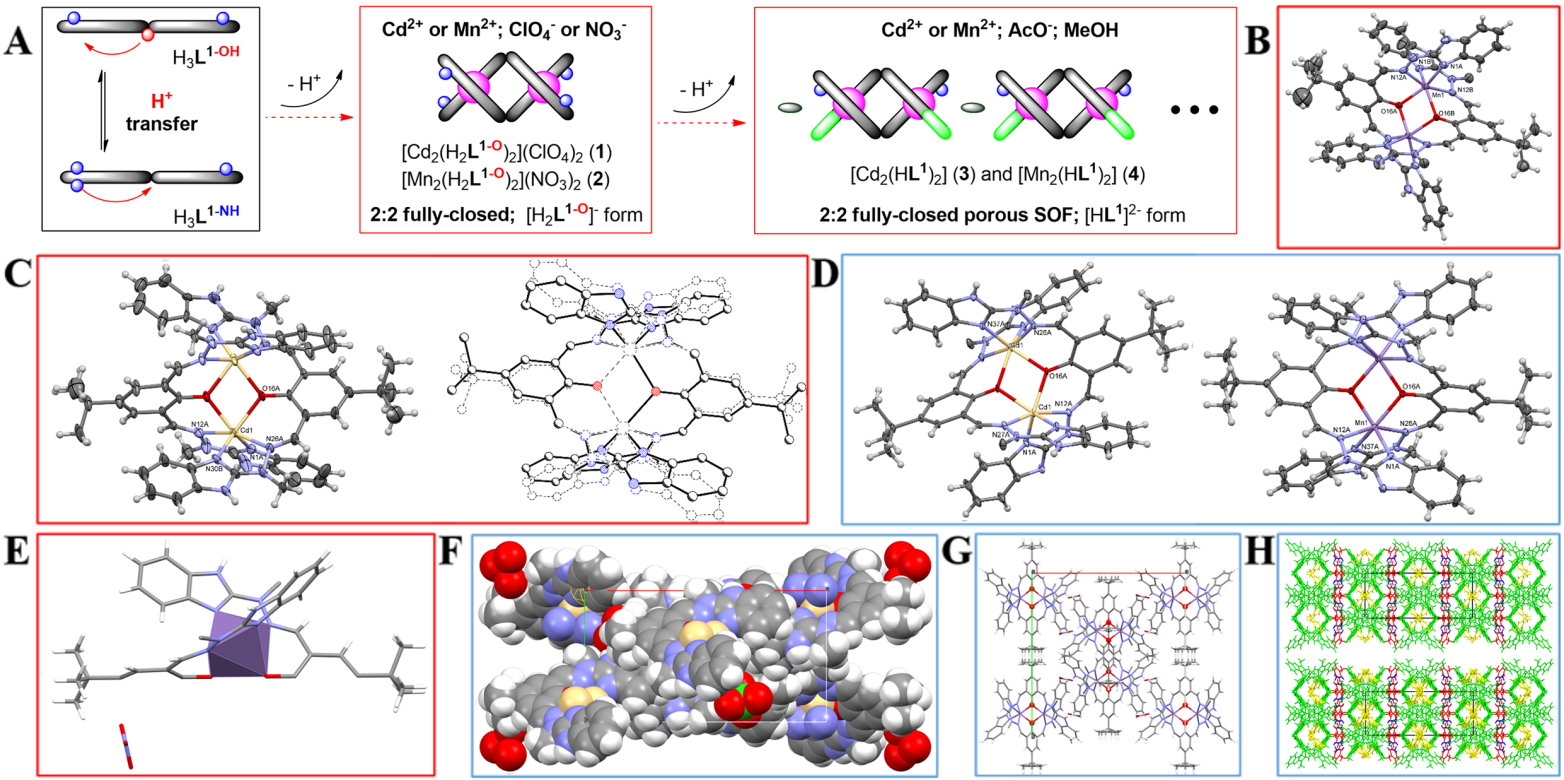
(**A**) Schematic representation of 2:2 **F–C** complexes; (**B**) Anisotropic ellipsoid representation (only one of the disordered *t*-Bu groups is shown for clarity) of complex (**2**); unlabelled atoms are related by symmetry operation 1–x,y,1/2–z.; (**C**) (left) Anisotropic ellipsoid representation of complex (**1**), non-labelled atoms are related to the labelled ones by symmetry operation 1–x,y,3/2–z; (right) comparison of two symmetry-independent molecules (see text).; (**D**) Anisotropic ellipsoid representation of the neutral complex (**3**) (left) and (**4**) (right); (**E**) Asymmetric unit of complex (**2**) with polyhedral representation of the central metal ion viewed perpendicular to the axis of phenoxide moieties; (**F**) Crystal packing of complex (**1**); (**G**); Crystal packing of complex (**2**); (**H**) Crystal packing of (**3**) (top) and (**4**) (bottom): green – complex, red/yellow – ordered/disordered toluene, blue – MeOH.

This complex belongs to the family of fully-closed **F–C** architectures and crystallizes in the C2/c space group, rendering the molecule *C*_2_-symmetrical (twofold axis passes through midpoint between two deprotonated phenoxy oxygen atoms). The asymmetric unit contains one Cd(II) ion, one ligand molecule and one disordered perchlorate anion. There is an interesting disorder, over a center of symmetry, leading to non-physically short interatomic contacts. The explanation can be found in the space-averaging nature of the diffraction element; the center of symmetry is not real; in fact, in the crystal structures there are two symmetry-independent molecules (in the space group C2), of slightly different conformation (Fig. 3C), but – in order to avoid strong correlations – it is easier to refine it as a disordered one in centrosymmetric space group. The complex is two-centered one, with almost planar Cd_2_O_2_ ring (roof angle of 0.2°). Cd(II) ions are 6-coordinated, in a distorted octahedral geometry. There are no solvent molecules in the structure, but there are relatively large voids which may accommodate small, disordered molecules. Hydrogen bond network connects the cations and anions into the crystal structure (Fig. 3F). Please note that we demonstrated the electrochemical sensing properties of the related [Mn_2_(H_2_**L**^**1–O**^)](ClO_4_)_2_ complex^52^ and here its magnetic properties will be studied (see Sect. “Magnetic properties of oligonuclear systems M_x_L_y_”).

##### [Mn_2_(H_2_L^1–O^)](NO_3_)_2_; (2)

The two-centered fully-closed **F–C** complex is also *C*_*2*_-symmetrical (twofold axis passes through C20A, C19A, C15A, O16A, O16B, C15B, C19 and C20 atoms) in the space group C2/c, with the exactly planar Mn_2_O_2_ ring (due to the symmetry). The asymmetric unit contains half of the Mn(II) ion, two halves of two ligand molecules and one nitrate anion. The *tert*-butyl groups are disordered over positions related by the twofold axis. Mn(II) cations are six-coordinated, in a distorted octahedral fashion (Fig. 3B). NO_3_^–^ anion exhibits strong coordinative tendencies to the metal ions, so here we expected, that it will coordinate, but surprisingly they are present only in the outer coordination sphere (Fig. 3E). This compound is very similar to the complex previously presented by us^52^, where non-coordinating ClO_4_^–^ ions were used, and to the isostructural cadmium analogue **1**. Crystal packing is showed in Fig. 3G. Magnetic properties Sect. “Magnetic properties of oligonuclear systems M_x_L_y_” will compare the effect of anion: NO_3_^–^ [Mn_2_(H_2_**L**^**1–O**^)](NO_3_)_2_ and ClO_4_^–^ [Mn_2_(H_2_**L**^**1–O**^)](ClO_4_)_2_.

##### [Cd_2_(HL^1^)_2_] (3) and [Mn_2_(HL^1^)_2_] (4)

These two complexes are highly isostructural: they crystallize in the same C2/c space group with very similar unit-cell parameters. They occupy the same positions in the respective unit cells and show almost identical crystal packing schemes, therefore they are discussed together (Fig. 3D,E). Similarity of Mn(II) and Cd(II) architectures was demonstrated for the cationic complexes **1** and **2**. Both complexes are *C*_*2*_-symmetrical (space group C2/c, twofold axis passes through the middlepoint of O···O), roof shaped with roof angles 6.9° for **3** and 6.0° for **4**. The asymmetric units contain one metal cation, one whole ligand molecule and additionally solvent: two toluene (of which one is disordered) and methanol molecules. The metal cations are six-coordinated in a distorted octahedral fashion. It might be noted that these are only neutral two-centered complexes in the studied series, as a result of an additional deprotonation of the benzimidazole NH ligand arm, facilitated by the choice of acetate anions. In the crystal structures (Fig. 3H) the apparent layers of complex and solvent molecules can be observed; there are hydrogen bonds between ligand and methanol molecules, resulting in the SOF (Supramolecular Organic Framework)^56^ architecture of potential porosity properties.

#### M^n+^: H_3_L^1^ equimolar, semi-open architectures (the ones where at least one benzimidazole arms becomes protonated—Fig. 4A)

##### [Zn(H_2_L^1–O^)(H_3_L^1–NH^)Zn(H_2_O)](OTf)_3_ (5)

**Figure 4:**
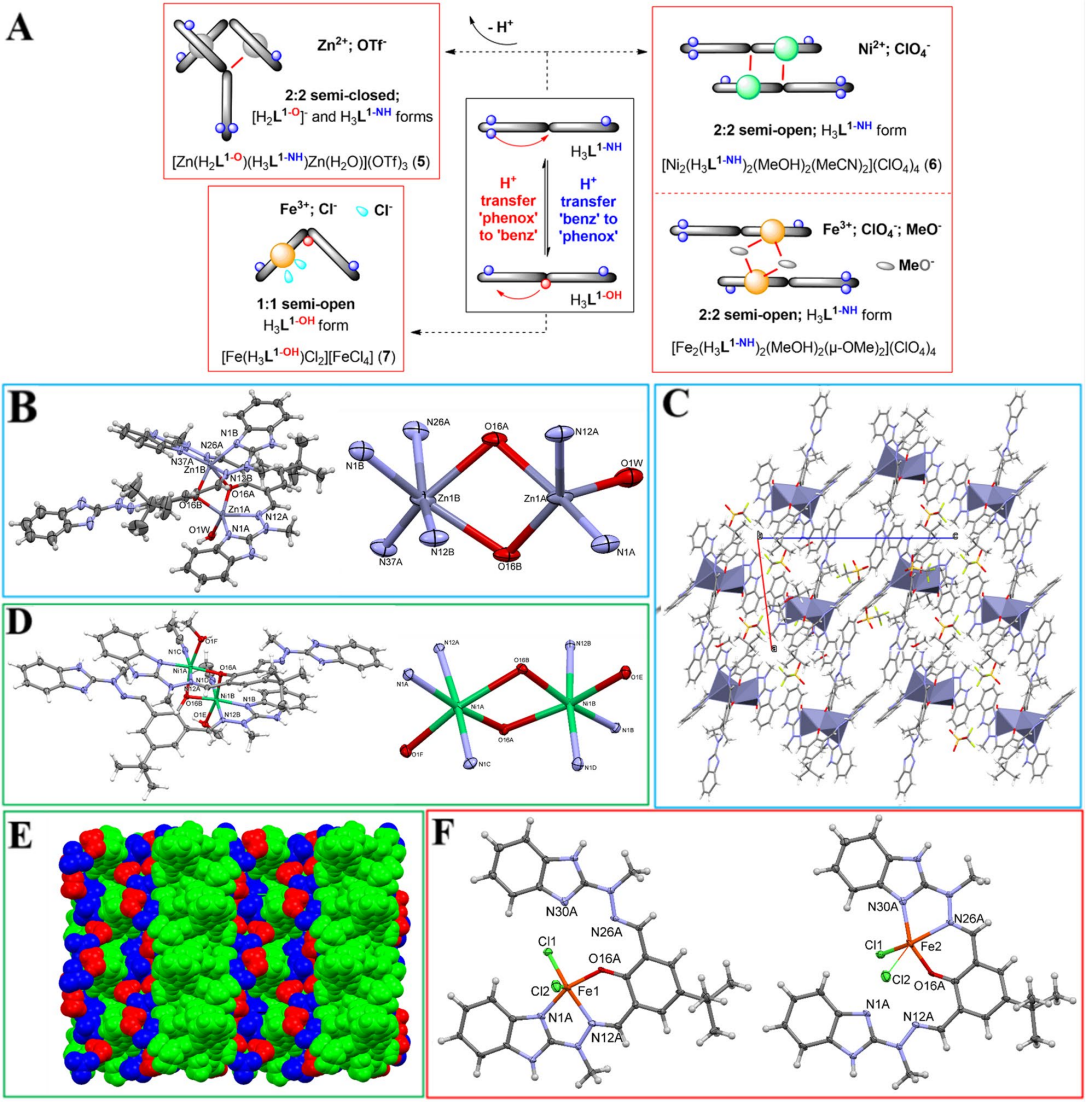
(**A**) Schematic representation of the obtained equimolar semi-open architectures; (**B**) (left) Anisotropic ellipsoid representation of complex (**5**) (here and throughout the paper the details as in Fig. 3); (right) Coordination schemes for Zn atoms; (**C**) crystal packing of complex (**5**) as seen along b-direction; (**D**) (left) Anisotropic ellipsoid representation; (right) Coordination schemes for Ni atoms; (**E**) Van der Waals spheres representation of the crystal structure, as seen along a-direction (green – complex, blue – perchlorates, red – methanols); (**F**) Anisotropic representation of more (left) and less (right) occupied complexes in the structure of (**7**).

This complex is roof-shaped two-centered, with two oxygen atoms from two ligand molecules at bridging position (Fig. 4B). The roof angle (defined as a dihedral between two ZnO_2_ planes) is 27.1(3)°. The peculiarity of the structure lies in the different coordination numbers of both Zn cations, and in different protonation states of the ligands. Zn1A is five coordinated, by two ligand nitrogen atoms, two bridging oxygens, and additionally by a coordinated water molecule; the coordination is close to square pyramid (τ parameter is 0.13), while Zn2 is six coordinated, in a distorted octahedral fashion, by four nitrogen atoms from two ligand molecules, and two bridging oxygens. In a consequence, the ligand A adopts the deprotonated [H_2_**L**^**1–O**^]^–^ form and uses five of its atoms for coordination, while ligand B adopts the H_3_**L**^**1**–**NH**^ form and uses only three dative bonds (with one benzimidazole arm protonated). In order to make reasonable hydrogen bonding scheme, ligand A is double-protonated, while ligand B – single, as it accepts the hydrogen bond from the methanol molecule. In the crystal structure, besides methanol, also the diisopropyl molecule found a place for itself. Hydrogen bonding connects all these elements into three dimensional network (Fig. 4C).

##### [Ni_2_(H_3_L^1–NH^)_2_(MeOH)_2_(MeCN)_2_](ClO_4_)_4_ (6)

This complex is also a roof shaped two-centered, with the roof angle of 16.18(6)°; however in this case both Ni(II) cations are six coordinated in a distorted octahedral fashion, by two nitrogen atoms and two bridging oxygen atoms from ligand molecules, and by one nitrogen from acetonitrile and one oxygen from methanol molecules (Fig. 4D). In the crystal structure (Fig. 4E), besides four perchlorate anions, four methanol molecules are also present and involved in extensive hydrogen bonding network. A rather similar complex was previously obtained by us^51^ with Fe(III) cations and ClO_4_^–^ anions—[Fe_2_(H_3_**L**^**1–NH**^)_2_(MeO)_2_(MeOH)_2_](ClO_4_)_2_, where the bridging character and [Fe_2_O_2_] core was a result of the contribution of the deprotonated methoxide groups.

##### [Fe(H_3_L^1–OH^)Cl_2_][FeCl_4_] (7)

It is a monomeric complex, in which iron cation occupies (with 87.6(3)%: 12.4(3)% frequency) two possible coordination sites (Fig. 4F), the result of the space-averaging by diffraction experiment; indeed in the crystal structures there are two distinguished complexes. The Fe ion is five coordinated (interestingly, in the less-occupied structure, one of the Fe-Cl bonds becomes quite long), in the slightly distorted square-pyramid fashion. This example shows that spherical anions of moderate coordination propensity can successfully lock only one of the benzimidazole arms, thus preventing more complex di- or polynuclear assemblies.

#### M^n+^: H_3_L^1^ varying ratios (M^n+^  < H_3_L^1^ and M^n+^  > H_3_L^1^) architectures (at least one benzimidazole arm becomes protonated—Fig. 5A)

##### [Co_4_(H_2_L^1–O^)_2_(OH)_2_(H_2_O)_2_(MeCN)(MeOH)](ClO_4_)_4_ (8)

**Figure 5:**
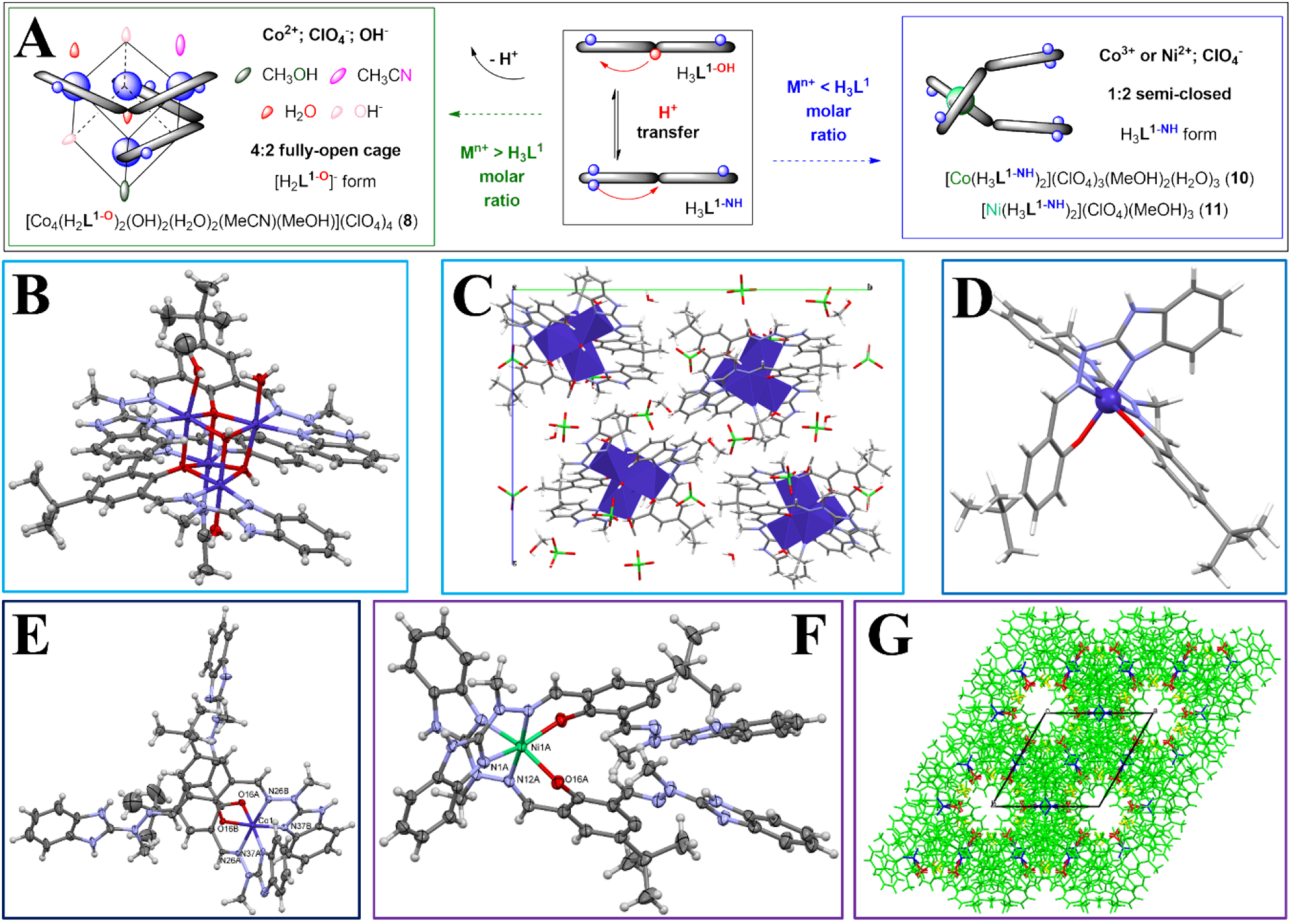
(**A**) Schematic representation of the obtained non-equimolar architectures; (**B**) Anisotropic ellipsoid representation of (**8**); (**C**) Unit cell and crystal packing of (**8**) along a axis; (**D**) Anisotropic ellipsoid representation of (**9**); (**E**) Anisotropic ellipsoid representation of **10**; (**F**) Anisotropic ellipsoid representation of **11**; unlabeled atoms are related by symmetry operation –x + y, y, 3/2–z; (**G**) in the crystal structure of (**11**) one can observe empty (or rather filled with the dispersed electron density, maybe disordered solvent) channels along six-fold axis z-direction in the crystal structure of complex (**11**).

Here four symmetry-independent Co(II) cations occupy the vertices of the approximate cube; the other four vertices come from two ligand oxygen atoms and two hydroxyl ions (Fig. 5B). All metal ions are six-coordinated in an octahedral fashion, the sixth coordination place (three are neighbouring O-vertices, two nitrogen atoms from ligand molecules) being water, acetonitrile and methanol. Four perchlorate anions, water and methanol molecules are involved in extensive network of H-bonds (Fig. 5C).

##### [Co(HL^2^)_2_](ClO_4_) (9)

This monomeric Co(III) complex crystallizes in P2_1_/c monoclinic space group with distorted, octahedral 6-coordinated fashion by four nitrogen atoms (two from imidazole ring and two imine nitrogen) and two oxygen atoms from ligand molecules (Fig. 5D). In the crystal structure, extensive H-bond network involves two water and one methanol molecules, creating three dimensional network of cations, anions, and neutral molecules. This is a structural analogue of H_3_**L**^**1**^ ligand with only one benz coordinating arm and ligand adopts a deprotonated form without the proton transfer to the benzmidazole arm (cf. with (**10**)).

##### [Co(H_3_L^1–NH^)_2_](ClO_4_)_3_(MeOH)_2_(H_2_O)_3_ (10)

This is nonsymmetrical, monomeric complex with octahedrally 6-coordinated Co(III) ion. Extensive H-bond network involves three water and two methanol molecules, creating three dimensional system of cations, anions, and neutral molecules (Fig. 5E). Interestingly, the ‘phenoxo’ part deprotonated but the proton is sequestered by the second benz arm, making it non-coordinating.

##### [Ni(H_3_L^1–NH^)_2_](ClO_4_)_2_(MeOH)_2_ (11)

This monomeric compounds crystallizes in relatively rare P6_1_22 hexagonal space group, in the CSD there are only 607 examples of such symmetry (combined for two enantiomeric space groups, P6_1_22 and P6_5_22), which is ca. 0.05% of the total number of structures deposited there. The complex is *C*_2_-symmetrical, as it lies across the twofold axis passing through the Ni(II) ion. Asymmetric unit contains one half of the Ni atom, one ligand molecule, two halfs of the perchlorate anion – also *C*_2_-symmetrical – and additionally two methanol molecules, one ordered and one disordered across the twofold axis. The coordination number for Ni(II) is 6, in quite regular octahedral geometry (Fig. 5F). In the crystal structure one can observe empty (or rather filled with the dispersed electron density, maybe disordered solvent) channels along six-fold axis (z-direction, Fig. 5G).

### Magnetic properties of oligonuclear systems M_x_L_y_

#### Mn(II) dimers—[Mn_2_(H_2_L^1–O^)](X or Y)_2_; (X–NO_3_ (2); Y–ClO_4_^52^)

**Figure 6:**
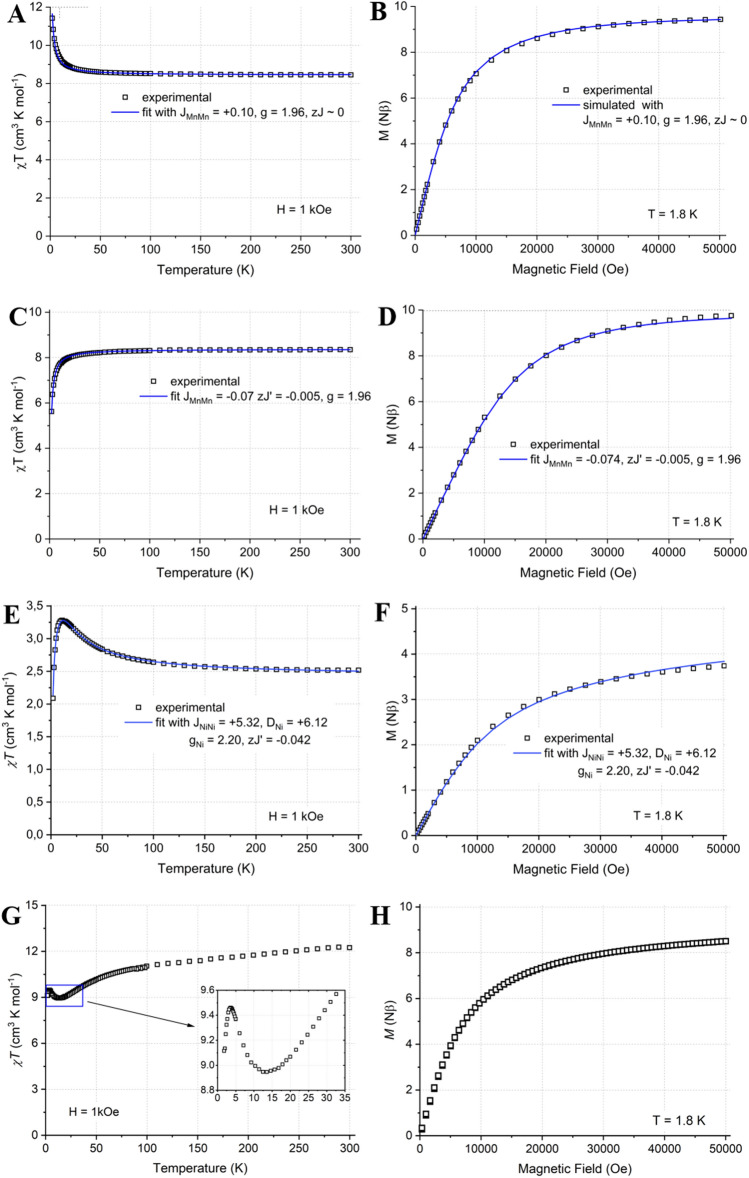
Magnetic properties of [Mn_2_(H_2_**L**^**1–O**^)](NO_3_)_2_ (**2**): (**A**) the thermal dependence of molar magnetic susceptibility *χT*(*T*) at *H* = 1 kOe and (**B**) the variation of the magnetization versus magnetic field *M*(*H*) at *T* = 1.8 K. Magnetic properties of [Mn_2_(H_2_**L**^**1-O**^)](ClO_4_)_2_^52^: (**C**) the *χT*(*T*) at *H* = 1 kOe and (**D**) the *M*(*H*) at *T* = 1.8 K. Magnetic properties of (**6**): (**E**) the *χT*(*T*) at *H* = 1 KOe and (**F**) the *M*(*H*) at 1.8 K. The relevant fits (**A**,**C**–**F**) or simulation (**B**) performed using the PHI^57^ software are shown as blue lines. The super-exchange magnetic coupling Hamiltonian term is – 2*JS*_**1**_**S**_**2**_. Magnetic properties of (**8**) (**G**) the *χT*(*T*) at *H* = 1 KOe and (**H**) the *M*(*H*) at *T* = 1.8 K. Inset (**G**): the *χT*(*T*) in low temperature region.

The thermal dependence of molar magnetic susceptibility *χT*(*T*) at H = 1 KOe (Fig. 6A) and variation of the magnetization versus magnetic field *M*(*H*) at 1.8 K for [Mn_2_(H_2_**L**^**1–O**^)](NO_3_)_2_ (**2**) (Fig. 6B) are presented. At 298 K the *χT* is equal 8.50 cm^3^·K·mol^−1^, which is comparable to 8.75 cm^3^·K·mol^−1^ calculated for two uncoupled Mn(II) centres of *S* = ^5^/_2_ and *g* = 2.0. On cooling *χT*(*T*) increases very slowly down to *ca*. 20 K, then rises more steeply to the value 11.4 cm^3^·K·mol^−1^ at 1.8 K. The *M*(*H*) dependence is close to the general shape of Brillouin function for *S* = 5 and *g*_iso_ slightly below 1.96. The *χT*(*T*) at *H* = 1 kOe (Fig. 6C) and *M*(*H*) at 1.8 K for [Mn_2_(H_2_**L**^**1–O**^)](ClO_4_)_2_^52^ (Fig. 6D) are presented. At 298 K the *χT* is equal 8.35 cm^3^·K·mol^−1^, which is comparable to 8.75 cm^3^·K·mol^−1^ calculated for two uncoupled Mn(II) centres of *S* = ^5^/_2_ and *g* = 2.0. On cooling *χT*(*T*) decreases very slowly down to *ca.* 20 K, then decline more steeply to the value 5.63 cm^3^·K·mol^−1^. The *M*(*H*) dependence run slightly below the general shape of Brillouin function for two uncoupled spins *S* = ^5^/_2_ and *g*_iso_ = 1.96 in the region below 50 kOe. The *χT*(*T*) and *M*(*H*) curves were reasonably reproduced using PHI software^57^ considering magnetic super-exchange interactions model for cyclic phenoxo-bridged Mn_2_O_2_ dimers. For compound [Mn_2_(H_2_**L**^**1–O**^)](ClO_4_)_2_^52^ very weak antiferromagnetic interactions with *J*_MnMn_ = – 0.07 cm^–1^ were found, being a value somehow smaller than those in the range of tenths to units of cm^–1^ found typically for molecular motifs of that type^58–60^. Conversely to that, compound **2** showed effective weak ferromagnetic interactions with *J*_MnMn_ =  + 0.1 cm^–1^. The above observations suggest that notable (in our weak interactions scale) ferromagnetic contribution is operating and could be attributed to the unique and sophisticated supramolecular synthons present in both crystal structures^61^. This include (i) the parallel π–π stacking and perpendicular C–H···centroid contacts involving the side systems of ligands and (ii) side hydrogen bonds involving benzimidazole N–H protons exposed fairly for external supramolecular bridging by NO_3_^–^ (**2**) or ClO_4_^–^^52^, forming a ladder of [Mn_2_(H_2_**L**^**1–O**^)_2_] bars. The small differences in Mn–O distances, Mn–O–Mn angles and Mn···Mn distances are rather of minor importance in face of literature systems. The detailed description of such complex magnetic supramolecular ladders composed of spins *S* = 5/2 would require more sophisticated treatment involving numerical fitting and DFT calculations, and can be a topic of further work.

#### Ni(II) dimer [Ni_2_(H_3_L^1–NH^)_2_(MeOH)_2_(MeCN)_2_](ClO_4_)_4_ (6)

The *χT*(*T*) at *H* = 1 KOe and *M*(*H*) at 1.8 K for (**6**) are presented in Fig. 6E,F. At 298 K the *χT* is equal 2.49 cm^3^·K·mol^−1^, which is lightly larger than 2 cm^3^·K·mol^−1^ calculated for two uncoupled Ni(II) centres of *S* = 1 and *g* = 2.0. On cooling *χT*(*T*) increases slowly to reach the maximum of 3.29 cm^3^·K·mol^−1^ in T = 11.4 K, then fall down sharply to 2.08 cm^3^·K·mol^−1^. The *M*(*H*) dependence runs slightly below the general shape of Brillouin function for two uncoupled spins *S* = 1 or one spin S = 2 (assuming *g*_av_ = 2), reaching 3.75 Nβ but not tending to quick saturation. The *χT*(*T*) and *M*(*H*) were fitted simultaneously using PHI^57^ software considering the ferromagnetic ground state *S*_gr_ = 2 and non-zero zero-field splitting (ZFS) parameter *D* for Ni(II) ions to yield reasonable parameters set of *J*_NiNi_ =  + 5.32 cm^–1^, *D*_Ni_ =  + 6.12 cm^–1^, *g*_Ni_ = 2.20 and *zJ*’ = − 0.042, in line with the results obtained for the similar systems^62–66^. The Ni–O–Ni angles of 97.2 and 98.2 deg in the Ni_2_O_2_ core locate very close to the critical point between the ferromagnetic domain (Ni–O–Ni smaller than 98 deg) and antiferromagnetic domain (Ni–O–Ni larger than 98 deg) and support the results of our magnetic fit. Moreover, the significant out-of-plane shift of phenyl rings and the notable hinge distortion of the core, here 18.7 deg, are in line with the effective ferromagnetic interactions. The fitting procedure disregarding the zero-field splitting (ZFS) *D* parameter gives an alike values set considering the *χT*(*T*) data only (*J*_NiNi_ =  + 5.9 cm^–1^, *g*_Ni_ = 2.20 and *zJ*’ = − 0.075), whereas the *M*(*H*) cannot be fairly reproduced in this way.

#### Co(II) cube [Co_4_(H_2_L^1–O^)_2_(OH)_2_(H_2_O)_2_(MeCN)(MeOH)](ClO_4_)_4_ (8)

The *χT*(*T*) at *H* = 1 KOe and *M*(*H*) at 1.8 K for (**8**) are presented in Fig. 6G,H. At 298 K the *χT* is equal 12.2 cm^3^·K·mol^−1^, which falls fairly within the range 10.8–13.6 cm^3^·K·mol^−1^ calculated for four uncoupled Co(II) centres of *S* = ^3^/_2_, assuming that *g* is located in range 2.4–2.7^67^. On cooling *χT*(*T*) decreases very slowly down to ca. 75 K, then decline more steeply to the minimum of 8.93 cm^3^·K·mol^−1^ at 13.6 K. Further it rises sharply to the maximum of 9.46 cm^3^·K·mol^−1^ at 3.7 K and, again, falls down to 9.11 cm^3^·K·mol^−1^ in 1.8 K limit. Such course of *χT*(*T*) locates fairly within the range of curves obtained by Sakiyama and Powell using the multi-parameter model involving contributions of appropriate Co–Co exchange coupling, zero-field splitting, spin–orbit coupling, orbital reduction and intermolecular interactions^68^. In particular, the distinct small maximum located in low temperature region (inset of Fig. 6G) indicates only a faint predomination of ferromagnetic interactions between effective spins *S*_eff_ = ^1^/_2_, in the face of the above mentioned model. The *M*(*H*) dependence tends very slowly to saturation and reach the value of 8.52 Nβ at H = 50 kOe. Such course of M(H) is consistent with the presence of weakly coupled four Co(II) ions with the effective spin *S*_eff_ = ^1^/_2_ and effective *g* value of 4.33 T due to combined effects of ZFS and SCO^67^. Some anisotropy can be present in this system, which is fairly indicated by the reduced anisotropy *M*(*H*/*T*) (not shown), however, no slow magnetic relaxation was found down to 1.8 K in any static field conditions, probably due to distinct combined supramolecular contacts along the hydrogen bonds and π-π interactions^69,70^.

### Solution NMR studies

**Figure 7:**
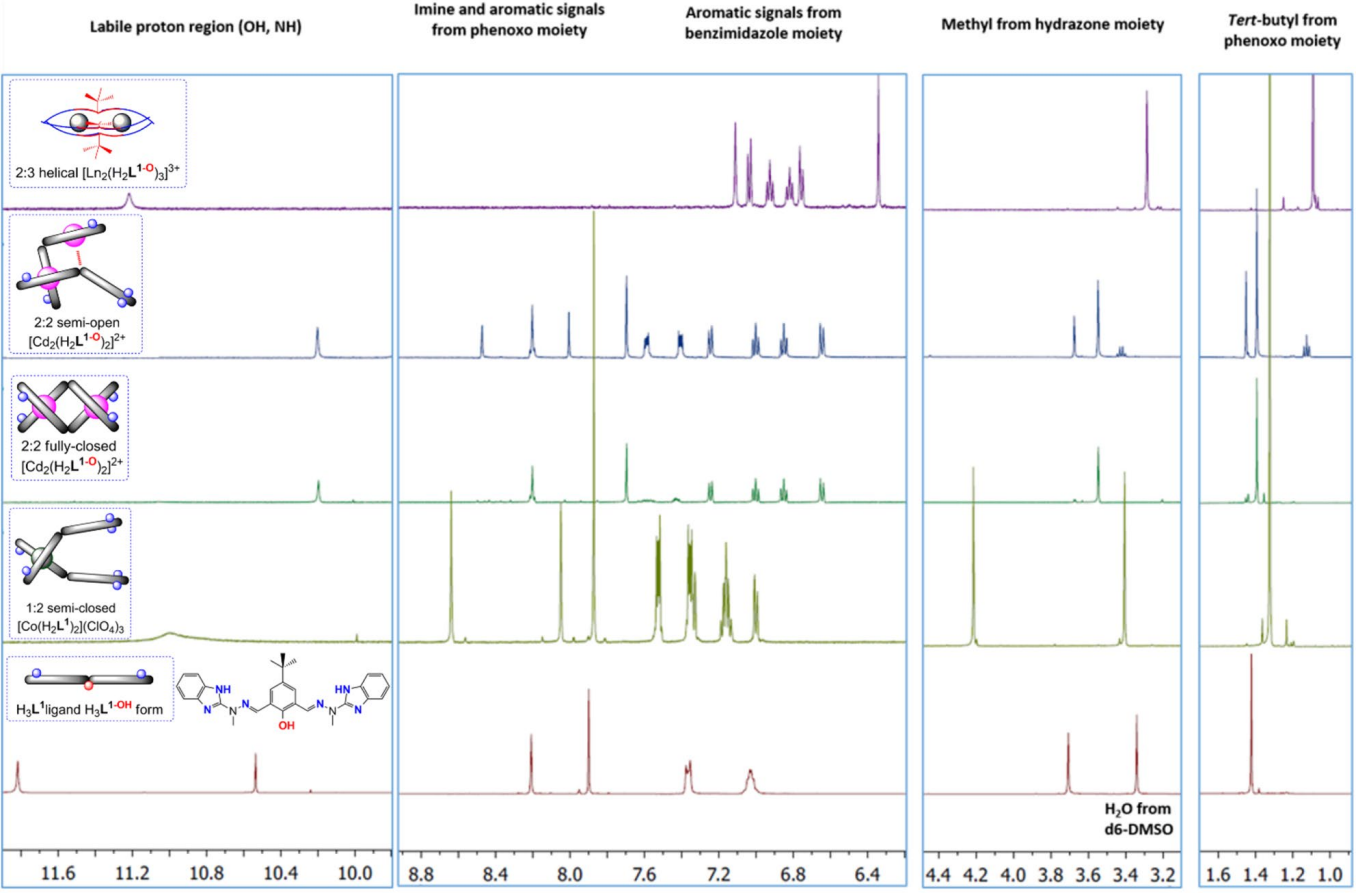
Representative ^1^HNMR spectra of stable supramolecular diamagnetic architectures from the H_3_**L**^**1**^ ligand with Co^III^ (1:2 semi-closed), Cd^II^ (2:2 fully-closed and semi-open) and La^III^ (2:3 helical) metal ions. Spectrum of ligand is in d6-DMSO, whereas the remaining ones are in CD_3_CN.

Comparison of X-ray solid state structures with diamagnetic coordination compounds (Co(III);ClO_4_^–^ – (**9**) and (**10**); Cd(II); ClO_4_ – (**1**); Zn(II);OTf^-^ – (**5**)) allowed us to better understand their solution behavior and the self-assembly algorithms of H_3_**L**^**1**^ ligand. Figure 7 shows ^1^HNMR spectra of H_3_**L**^**1**^ ligand and representative diamagnetic architectures observed in solution. Aliphatic *t*-Bu and hydrazone-methyl singlet signals are clearly observed in the low ppm regions (1.0–1.5 and 3.5–4.0 respectively); for H_3_**L**^**1**^-based compounds, the methyl group is sensitive to duplication, which then indicates desymmetrization of coordinated ligand or more complex architectures formed. The 6.2–8.7 ppm aromatic region covers the remaining non-labile protons, with the benzimidazole arm (up to 7.6 ppm), the imine and phenoxo (above 7.7 ppm) signals being clearly discernible. Again, multiplication of signals is indicative for coordination modes that are other than the symmetrical ones. The most downfield shifted signals are attributed to the -OH and -NH groups, however their presence is dependent on the nature of the complex as well as solvent.

Comparison of H_3_**L**^**1**^ and H_2_**L**^**2**^ ligands with their thermodynamically stable and inert Co^III^ complexes (**9**, **10**) gives a ^1^HNMR fingerprint of the non-coordinated ligand frameworks and the semi-closed **S–C** [ML_2_] moieties (Fig. S3). Interestingly, in situ reaction of H_3_**L**^**1**^ with twofold excess of CoCl_2_ under oxidative conditions, gives ^1^HNMR spectrum that is similar to the [Co(H_2_**L**^**1**^)_2_]^+^ (Fig. S4) but differences in the labile proton region and exchangeable acetonitrile solvent signals suggest the [Co^III^(H_3_**L**^**1-OH**^)Cl_2_]Cl architecture, of similarity to the Fe^III^ complex (**7**). This would suggest that the chloride anion is rather effective in maintaining the 1:1 semi-open architectures also in d3-MeCN solution, not only in the solid state. Titration of [Co(H_3_**L**^**1–NH**^)_2_](ClO_4_)_3_(MeOH)_2_(H_2_O)_3_ (**10**) with Ag^I^ triflate unambiguously confirms the locked character of the non-coordinating benzimidazole arm as a result of the proton transfer from the phenoxo group (Fig. S5).

Closed-shell *d*^10^ metal ions like Cd^II^ and Zn^II^ are known for their labile character in solution^71, 72^ and therefore compounds (**1**) and **5** where dissolved in different solvents. For compound (**5**), very complex spectra were observed, which precluded any meaningful characterization; most probably the already complex dissymmetric character of the Zn^II^ compound observed in the solid state is further amplified with the additional dissociative equilibria (Fig. S6).

An interesting situation however is observed for the Cd^II^ complex. The immediate dissolution of crystals of (**1**) gives ^1^HNMR ascribed to the highly symmetric 2:2 fully-closed **F–C** structure observed in the solid state, which almost immediately starts to transform to the 2:2 semi-open **S–O** architecture (Fig. 7). Time and solvent dependent spectra show subsequent generation of additional species in CD_3_CN, which can be however ‘reseted back’ upon heating (Fig. S7). Such time and temperature dependent equilibrium is not observed in CD_3_OD, where only the 2:2 **S–O** system is thermodynamically most stable (Fig. S8). Overall, such comparison shows that for Cd(II) and Zn(II) more complex species can also form, related to the lack of crystal field stabilization effects (contrary to e.g. low spin Co^III^ species). This is also responsible for changes observed in the absorption and emission spectra (see Sect. “Absorption and emission measurements”) Finally, complexation of ligand H_3_**L**^**1**^ with diamagnetic La(OTf)_3_ allows one to observe a single set of species, which can be ascribed to the bimetallic triple-stranded helicates, per similarity to the previously observed systems (Fig. 7 top)^49,50^.

### Absorption and emission measurements

Complexes (**1**) ([Cd_2_(H_2_**L**^**1–O**^)](ClO_4_)_2_), (**5**) ([Zn(H_2_**L**^**1–O**^)(H_3_**L**^**1–NH**^)Zn(H_2_O)](OTf)_3_), (**12**) (Zn(H**L**^**2**^)_2_) and (**13**) (Cd(H**L**^**2**^)_2_) were chosen as a representative examples for the absorption and emission measurements, thanks to their emission and structural properties. Complexes (**1**) and (**5**) represent the group of structural diversity with the H_3_**L**^**1**^ ligand (see Sects. “Solid state structural description of synthesized architectures” and “Solution NMR studies”), whereas (**12**) and (**13**) are stable, neutral components with deprotonated form of ligand H_2_**L**^**2**^. The aim of this measurements was to check how the possible structural transformations in solution influence their absorption/emission properties. Measurements were carried out at room temperature in four different solvents (MeOH, MeCN, DMF and DMSO) and the absorption spectra of compounds (**1**), (**5**), (**12**), (**13**) are shown in Figs. S9–S12 in SI. The characteristic absorption bands between 240 and 380 nm in all complexes can be assigned to π → π* transitions of the Schiff-base ligands and their intensity is time- and solvent-dependent^73, 74^.

Emission measurements were carried out for complexes in the solvents mentioned above immediately after dissolution and after five days from dissolution (**P**). All spectra were shown in Figs. S13–S16 in SI. Highest emission intensity for (**1**) and (**13**) was in MeOH, with an excitation wave of 389 nm, with a maximum at 476 nm for (**1**) and with an excitation wave of 379 nm with a maximum at 457 nm for (**13**). Emission measurements (**P**) of (**1**) showed in MeCN, a fourfold increase in the emission intensity and for MeOH a two-fold increase in the emission intensity in comparison with the intensities obtained immediately after dissolution, which would imply that the dissymmetric architectures formed in solution (see Sect. “Absorption and emission measurements”) are beneficial from the standpoint of emission properties. For (**13**) only a slight increase in intensity was noted, but in MeCN formation of second band with maximum at 470 nm was observed, possibly connected to aggregation^75^. The similar behaviour was observed for (**12**), but the maximum of the second band was at 430 nm. In (**5**) no changes were noted. As we can observe, for (**1**), the changes in emission intensity were the highest probably connected with defined structural changes. For “open” system (**5**) and stable complexes (**12**) and (**13**) only slight changes were noticed. Quantum efficiency measurements were carried out for complexes that exhibited better emission properties (**1** and **5**) (see Table S4 for details). We also checked the emission properties in solid state, where no changes were noticed, which confirmed, that changes are only present in solution (see Fig. S17 in SI).

To investigate the forms of ligand present in solution, titrations were carried out with the acid–base, the changes were tracked by UV–VIS spectroscopy (Fig. S18). This experiment helped illustrate the ligand forms present in solution, and thus proved that pH has a strong influence in the process of self-organization, and thus on the obtained structures of complex compounds.

## Materials and methods

The metal salts, organic compounds and solvents were supplied by Merck Chemical Company and POCH. All chemicals mentioned above were of analytical grade quality and were used as obtained without further purification. Fourier Transform Infrared (FT-IR) spectra were performed by means of a FT-IR Bruker IFS 66v/S spectrophotometer, in the range between 400 and 4000 cm^–1^ with a resolution of 4 cm^–1^. An average of 24 scans has been carried out for each sample. The samples were prepared on a KBr pellet under a pressure of 0.01 torr. Mass spectra (ESI–MS) were determined by a Waters Micromass ZQ spectrometer in acetonitrile or methanolic solutions with concentrations ∼10^−4^ M. The samples were run in the positive-ion mode. Sample solutions were introduced into the mass spectrometer source with a syringe pump with a flow rate of 40 μL min^–1^ with a capillary voltage of + 3 kV and a desolvation temperature of 300 °C. Source temperature was 120 °C. Cone voltage(Vc) was set to 30 V to allow transmission of ions without fragmentation processes. Scanning was performed from m/z = 100 to 2000 for 6 s, and 10 scans were summed to obtain the final spectrum. Simulations of mass spectra were conducted with enviPat programme^76^. Microanalyses were performed using a Elementar Analyser Vario EL III. NMR spectra were run on a Spektrometer NMR Varian VNMR-S 400 MHz spectrometer and were calibrated against the residual protonated solvent signals (DMSO-d6, d 2.50) which are given in parts per million. All electronic absorption spectra were recorded with a Shimadzu UVPC 2001 spectrophotometer, between 220 and 800 nm, in 10 × 10 mm quartz cells using solutions 2 × 10^–5^ M with respect to the metal ions. Excitation and emission spectra were measured at room temperature on a Hitachi 7000 spectrofluorimeter with excitation and emission slits of 2.5 nm. Magnetic properties were measured using QD MPMS 5 XL magnetometer. The samples were sealed in plastic foil before the measurements. The (**8**) was measured in the residue of mother liquor due to its instability in air. The original emu signals were carefully corrected in respect to all diamagnetic contributions (foil and molecular diamagnetism). All fitting and simulations were performed using the procedures included in PHI software^57^. The detailed synthetic procedures are demonstrated in the Supporting Information section.

## Conclusions

We demonstrated the rich structural diversity of the benzimidazole/phenoxo ligand H_3_**L**^**1**^ in the presence of variety of *d*-block metal ions, which is tunable through the pH, nature of the metal and its molar ratio with respect to the ligand, counterions and solvent. 10 new solid state structures were obtained (12 overall) in the solid state, which demonstrated the possibility to form: 1:1 and 2:2 semi-open, 2:2 semi-closed and fully-closed architectures, the latter of which can be subsequently deprotonated to induce the porous character and form the SOF-type materials. When ligand is subjected to non-equimolar mixtures, 1:2 semi-closed or 4:2 fully-open cage-like entities can be obtained, which overall demonstrate the various protonation/deprotonation levels of the H_3_**L**^**1**^ ligand and how it affects the final metallosupramolecular architectures. With the help of the H_2_**L**^**2**^ ligand analogue with only one benzimidazole pending arm, improved understanding of the solid state/solution behavior of the diamagnetic architectures was ascertained. The dynamic equilibrium was observed for the d^10^-species, which affected the absorption and emission properties of the Zn(II) and Cd(II) compounds under study. The solvent and anions were also crucial to the construction of the various architectures. Chosen paramagnetic compounds were studied for their magnetic properties to further demonstrate how small structural differences elicit significantly varied magnetic responses. Ni(II) and Mn(II) dimeric supramolecular architectures are very peculiar examples, where the [M_2_O_2_] core is at the structural intersection of the transition within the magnetic exchange interactions, resulting in tunable ferromagnetic or antiferromagnetic interactions.

Current investigation further reinforces that combined H-bonding and dative bonds can successfully lead to variety of supramolecular architectures, with the final properties/function being dependent on the final composition, rendering the a priori, pre-programmed assembly even a bigger challenge. The systematic exploration of these systems will focus in the future on transfer of the observed properties to functional materials.

### Supplementary Information

Supplementary Information 1.

Supplementary Information 2.

## Data Availability

Crystallographic data (excluding structure factors) for the structural analysis has been deposited with the Cambridge Crystallographic Data Centre, Nos. CCDC-1482453-1482463 for compounds [H_5_**L**^**1**^](ClO_4_)_2_, **1** – **11**. Copies of this information may be obtained free of charge from: The Director, CCDC, 12 Union Road, Cambridge, CB2 1EZ, UK. Fax: + 44(1223)336-033, e-mail:deposit@ccdc.cam.ac.uk, or www: www.ccdc.cam.ac.uk. The data that support the findings of this study are available from the corresponding author upon reasonable request.
