# Supplementary material for: Unexpected structural complexity of d-block metallosupramolecular architectures within the benzimidazole-phenoxo ligand scaffold for crystal engineering aspects
Source: Sci Rep. 2023 Oct 23;13:18055. doi: 10.1038/s41598-023-45109-z (PMC10593740; doi:10.1038/s41598-023-45109-z)
Supplement: Supplementary file 1 — Supplementary Information 1. [file 41598_2023_45109_MOESM1_ESM.pdf]

Unexpected structural complexity of *d*-block metallosupramolecular architectures within the benzimidazole-phenoxo ligand scaffold for crystal engineering aspects

Dawid Marcinkowski,[a] Maciej Kubicki,[a] Giuseppe Consiglio,[b] Zbigniew Hnatejko,[a] Anna M. Majcher-Fitas,[c] Robert Podgajny, [d] Violetta Patroniak,[a] Adam Gorczyński[a]*

[a]Faculty of Chemistry, Adam Mickiewicz University, Uniwersytetu Poznańskiego 8, 61-614 Poznań, Poland
[b]Dipartimento di Scienze Chimiche, Università di Catania, I-95125 Catania, Italy
[c]Faculty of Physics, Astronomy and Applied Computer Science, Jagiellonian University, Łojasiewicza 11, 30-348 Kraków, Poland
[d]Faculty of Chemistry, Jagiellonian University, Gronostajowa 2, 30-387 Krakow, Poland

corresponding author email: adam.gorczynski@amu.edu.pl

Supplementary Information

Table of contents:

**I.**	**Materials and methods**	**S3**
**II.**	**Experimental section**	**S4**
**Scheme S1**	**Synthesis of ligand H_3L^1**	**S4**
**Spectrum 1**	**^{1}H NMR of H_3L^1**	**S4**
**Scheme S2**	**Synthesis of ligand H_2L^2**	**S5**
**Spectrum 2**	**^{1}H NMR of H_2L^2**	**S5**
**Spectrum 3**	**ESI-MS spectra of 8**	**S9**
**Spectrum 4**	**^{1}H NMR of 9**	**S10**
**Spectrum 5**	**ESI-MS spectrum of 9 with theoretical prediction in red**	**S11**
**Spectrum 6**	**^{1}H NMR of 12.**	**S12**
**Spectrum 7**	**^{1}H NMR of 13.**	**S13**
**III.**	**Crystallographic data**	**S14**
**Figure S1**	**(top left) Anisotropic ellipsoid representation of the molecule A of a cation $(H_5L^1)^{2+}$; ellipsoids are drawn at the 50% probability level, hydrogen atoms are shown as spheres of arbitrary radii, hydrogen bond is shown as thin blue line. (top right) Comparison of two symmetry-independent cations (fitting of the central rings).[1]**	**S15**
**Figure S2**	**A crystal structure of $[H_5L^1](ClO_4)_2$ as seen along b-direction. Anions and solvent molecules are shown in van der Waals spheres representation in order to visualize their filling-structure role.**	**S16**
**Table S1**	**Relevant geometric parameters (Å, °).A, B, C, D, E – mean planes of subsequent planar fragments: A: N1 – N9; B: C2, N10, N12, C13, C14; C: C14, C15, C17, C18, C19, C24; D: C17, C25, N26, N27, C29; E: C30 – N37. The three (for c.n. 6) or two (c.n. 5) largest angles around metal ion are listed**	**S16**
**Table S2**	**Hydrogen bond data (Å, °)**	**S18**
**IV.**	**^{1}H NMR solution studies**	**S21**
**Figure S3**	**Comparison of H_3L^1 and H_2L^2 ligands with their semi-closed 1:2 complexes with Co^{III} ions in the presence of perchlorate. Please note that solvent for ligands is d^6-DMSO, whereas for complexes it is CD_3CN.**	**S21**

**Figure S4**	**Comparison of H_3L^1 ligand with semi-closed 1:2 complexes with Co^{III} ions in the presence of perchlorate and chloride anions. Please note that solvent for ligand is d^6-DMSO, whereas for complexes it is CD_3CN**	**S21**
**Figure S5**	**Titration of $[Co(H_3L^{1-NH})_2](ClO_4)_3$ (10) with AgOTf in CD_3CN**	**S22**
**Figure S6**	**Comparison of H_3L^1 ligand with semi-closed 2:2 Zn(OTf)2 complexes in deuterated acetonitrile and DMSO**	**S22**
**Figure S7**	**Time-and temperature dependent changes of the $[Cd_2(H_2L^{1-O})_2](ClO_4)_2$; (1) complex in CD_3CN solvent.**	**S23**
**Figure S8**	**Time-and temperature dependent changes of the $[Cd_2(H_2L^{1-O})_2](ClO_4)_2$; (1) complex in CD_3OD solvent.**	**S23**
**V.**	**Absorption and emission spectra**	**S24**
**Scheme S3**	**Possible forms of H_3L^1 ligand in a function of their protonation and deprotonation.**	**S24**
**Figure S9**	**Absorption spectra of compound 1 in different solvents**	**S24**
**Figure S10**	**Absorption spectra of compound 13**	**S25**
**Figure S11**	**Absorption spectra of compound 5 in different solvents**	**S25**
**Figure S12**	**Absorption spectra of compound 12 in methanol**	**S25**
**Table S3**	**Molar extinction coefficients for compounds 1, 5, 12, 13**	**S26**
**Figure S13**	**Emission spectra of compound 1 in: a) methanol, b) acetonitrile, c) dimethylformamide, d) dimethylsulfoxide. P- measurement after 5 days of dissolution**	**S26**
**Figure S14**	**Emission spectra of compound 13 in: a) methanol, b) acetonitrile, c) dimethylformamide, d) dimethylsulfoxide. P- measurement after 5 days of dissolution**	**S27**
**Figure S15**	**Emission spectra of compound 5 in: a) methanol, b) acetonitrile, c) dimethylformamide, d) dimethylsulfoxide. P- measurement after 5 days of dissolution**	**S27**
**Figure S16**	**Emission spectra of compound 12 in: a) methanol, b) acetonitrile, c) dimethylformamide, d) dimethylsulfoxide. P- measurement after 5 days of dissolution**	**S28**
**Table S4**	**Collected results of quantum yields for complexes 1 and 5 with schematic representation.**	**S28**
**Figure S17**	**Solid state emission spectra for compound 1, 5, 12, 13**	**S29**
**Figure S18**	**Absorption spectra of titration of ligand L^1 (up) with acid - HCl and (bottom) with base – triethylamine ($c = 2 \cdot 10^{-5}$ M in methanol).**	**S30**
**VI.**	**Literature**	**S31**

I. Materials and methods

The metal salts, organic compounds and solvents were supplied by Merck Chemical Company and POCH. All chemicals mentioned above were of analytical grade quality and were used as obtained without further purification. Fourier Transform Infrared (FT-IR) spectra were performed by means of a FT-IR Bruker IFS 66v/S spectrophotometer, in the range between 400 and 4000 cm^{-1} with a resolution of 4 cm^{-1}. An average of 24 scans has been carried out for each sample. The samples were prepared on a KBr pellet under a pressure of 0.01 torr. Mass spectra (ESI-MS) were determined by a Waters Micromass ZQ spectrometer in acetonitrile or methanolic solutions with concentrations $\sim 10^{-4}$ M. The samples were run in the positive-ion mode. Sample solutions were introduced into the mass spectrometer source with a syringe pump with a flow rate of 40 $\mu L\ min^{-1}$ with a capillary voltage of +3 kV and a desolvation temperature of 300ºC. Source temperature was 120ºC. Cone voltage(Vc) was set to 30 V to allow transmission of ions without fragmentation processes. Scanning was performed from m/z = 100 to 2000 for 6 s, and 10 scans were summed to obtain the final spectrum. Simulations of mass spectra were conducted with enviPat programme[2]. Microanalyses were performed using a Elementar Analyser Vario EL III. NMR spectra were run on a Spektrometer NMR Varian VNMR-S 400 MHz spectrometer and were calibrated against the residual protonated solvent signals (DMSO-d6, d 2.50) which are given in parts per million. All electronic absorption spectra were recorded with a Shimadzu UVPC 2001 spectrophotometer, between 220 and 800 nm, in 10 x 10 mm quartz cells using solutions 2 x 10^{-5} M with respect to the metal ions. Excitation and emission spectra were measured at room temperature on a Hitachi 7000 spectrofluorimeter with excitation and emission slits of 2.5 nm. Magnetic properties were measured using QD MPMS 2 XL magnetometer. The samples were sealed in plastic foil before the measurements. The **8** was measured in the residue of mother liquor due to its instability in air. The original emu signals were carefully corrected in respect to all diamagnetic contributions (foil and molecular diamagnetism). All fitting and simulations were performed using the procedures included in PHI software.[3]

II. Experimental section

Synthesis of ligand H_3L^1

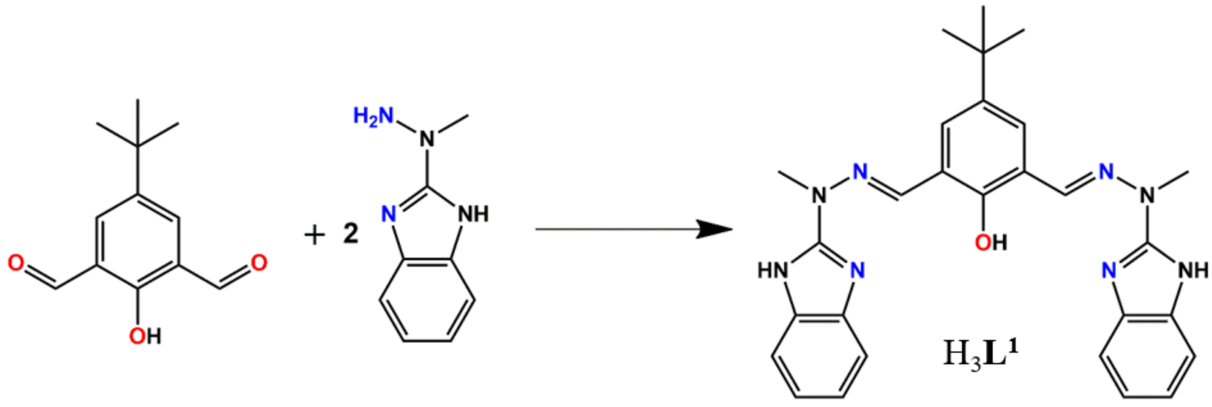

Scheme S1. Synthesis of ligand $H_3\mathbf{L^1}$.

Ligand $H_3\mathbf{L^1}$ was prepared as reported previously.[1] After synthesis ^{1}H NMR was performed to confirm structure and purity of final product. ^{1}HNMR (400 MHz, d_6 -DMSO): δ = 1.42 (s, 9H), 3.71 (s, 6H), 7.03 (m, 4H), 7.36 (m, 4H), 7.90 (s, 2H), 8.21 (s, 2H) ppm.

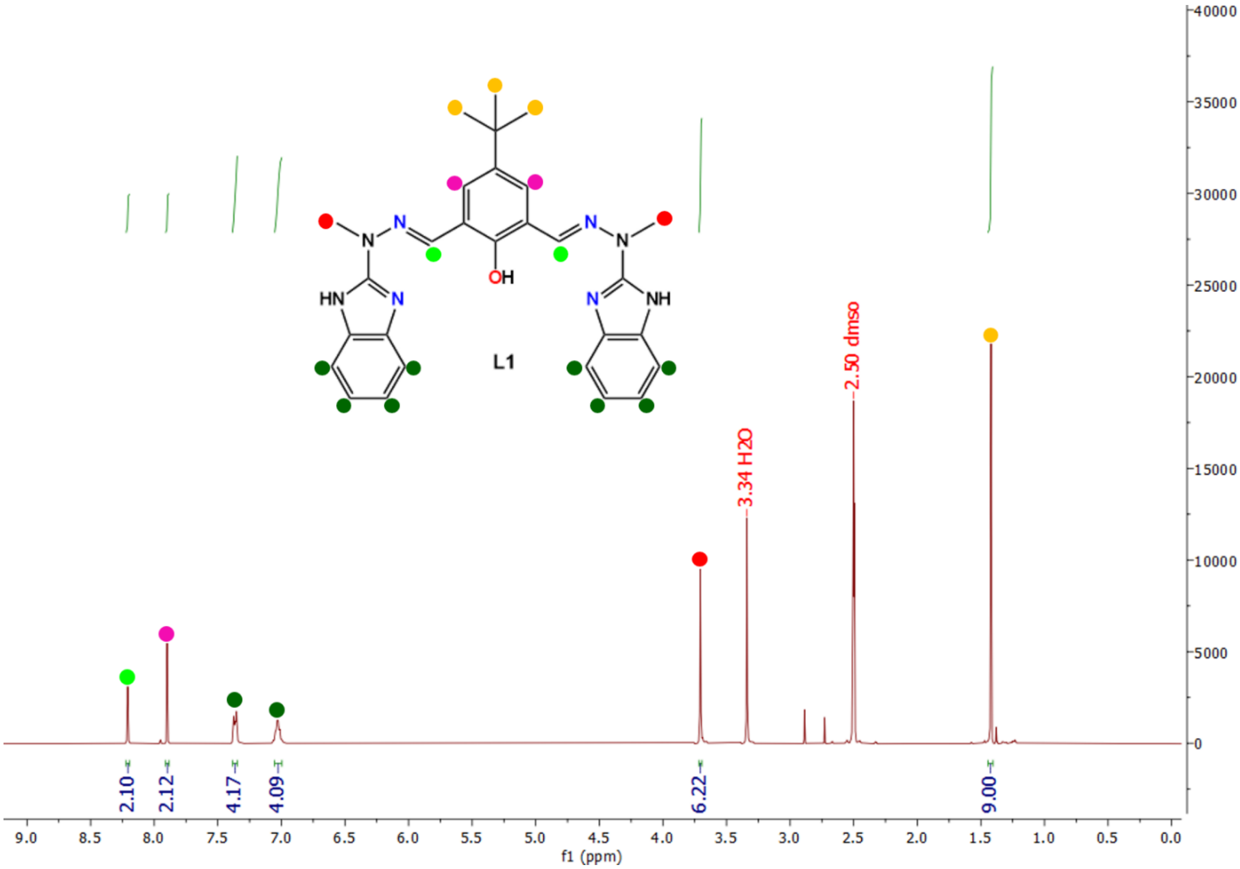

Spectrum 1. ^{1}H NMR of $H_3\mathbf{L^1}$.

Synthesis of ligand H$_2$L^2

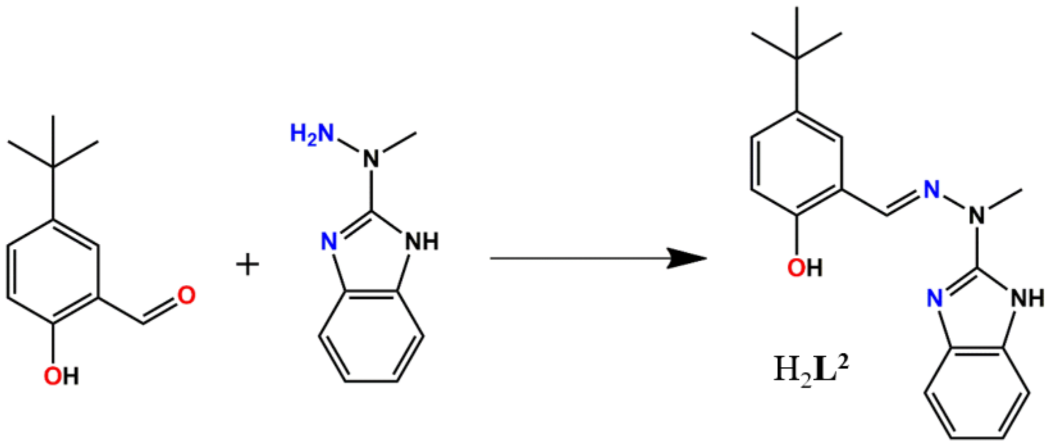

Scheme S2. Synthesis of ligand H$_2$**L**^{2}.

Ligand H$_2$**L**^{2} To a 50 ml pressure tube containing 2-(1-methylhydrazinyl)-1Hbenzo[d]imidazole (synthesized via slightly modified procedure adapted from Povstyanoi et al., yield: 90%) (53.3 mg, 0.33 mmol), 5-tert-butyl-2-hydroxybenzaldehyde (58.8 mg, 0.33 mmol) dissolved in 20 ml of EtOHabs was added. Clear reaction mixture was flushed with inert gas, stirred and heated in reflux for 4h, which resulted in formation of yellow precipitate. Residue were filtered via suction filtration and dried in the vacuum.

Yield: 98.0 mg (93.0 %) ^{1}HNMR (400 MHz, d$_6$ -DMSO): δ = 1.33 (s, 9H), 3.65 (s, 3H), 6.85 (d, 1H), 7.01 (d, 2H), 7.25 (dd, 1H), 7.34 (m, 2H), 7.89 (d, 1H), 8.07 (s, 1H) ppm.^{13}CNMR (125 MHz, d$_6$ -DMSO): δ = 31.4, 31.7, 33.9, 115.6, 119.9, 123.48, 127.12, 135.6, 141.5, 153.5, 153.8 ppm.

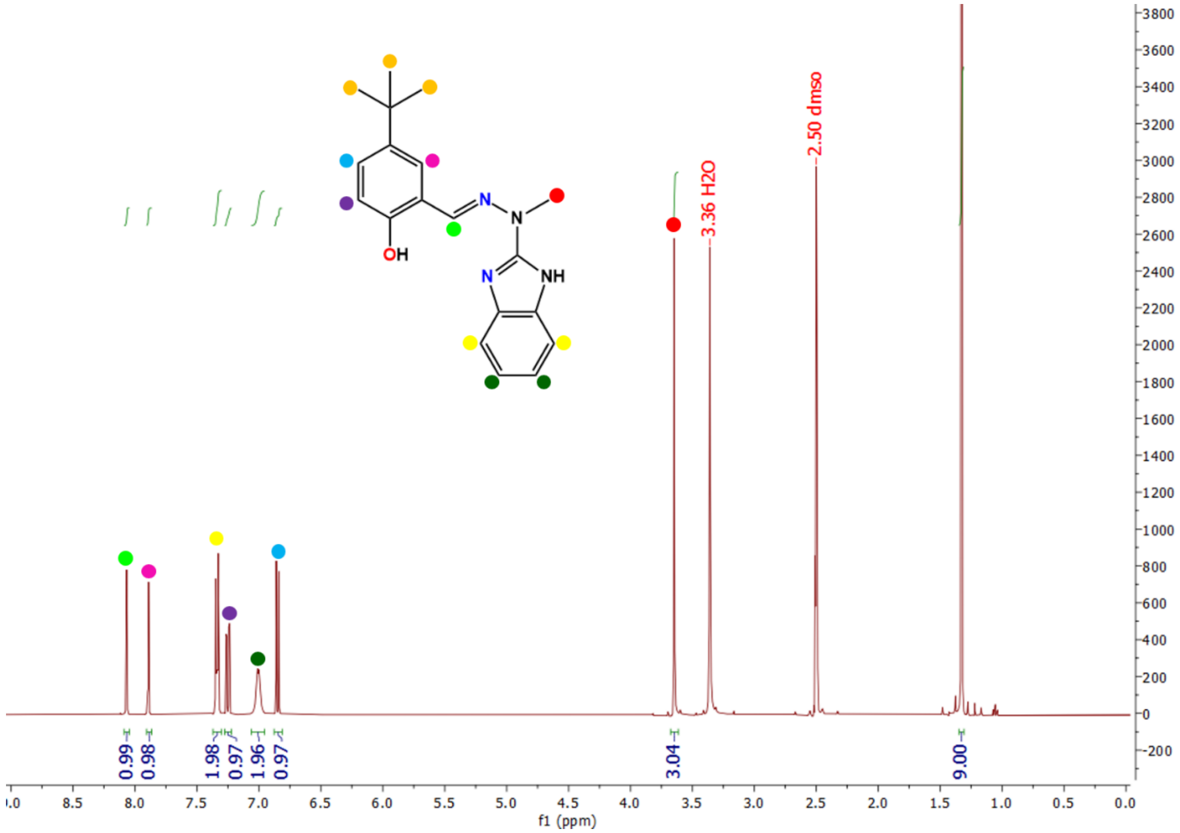

Spectrum 2. ^{1}H NMR of H$_2$**L**^{2}.

Complexes (**1 - 8**) and (**10 - 11**) were prepared in the same manner. To a solution of ligand H_3**L**[1] (100.0 mg, 0.20 mmol) the appropriate metal salt was added (0.20 mmol) ($Cd(ClO_4)_2 \cdot 6H_2O$ – **1**, $Mn(NO_3)_2 \cdot 4H_2O$ – **2**, $Cd(CH_3COO)_2 \cdot 2H_2O$ – **3**, $Mn(CH_3COO)_2 \cdot 2H_2O$ – **4**, $Zn(CF_3SO_3)_2$ – **5**, $Ni(ClO_4)_2 \cdot 6H_2O$ – **6**, **11**, $FeCl_3 \cdot 6H_2O$ – **7** $Co(ClO_4)_2 \cdot 6H_2O$ – **8**, **10**) in 15 ml of MeOH/MeCN (1:1 v:v). Yellow (**1-6**, **11**), orange (**8**, **10**), dark green (**7**) solution formed instantly and then triethylamine (0.20 mmol) was added. The color of the solutions has changed to a more intense one and the reaction mixtures were stirred for 24 h at room temperature. After evaporation of solvents under reduced pressure, the residues were dissolved in minimum volume of MeOH/MeCN (1:1 v:v) and precipitated by excess of Et_2O. Yellow (**1-6**, **11**), brown (**8**, **10**), dark green (**7**) solids were filtered via suction filtration and dried in the vacuum. Compounds (**6** - yellow) and (**11** - yellow) were obtained from reaction (**6**) by recrystallization of crude product via slow diffusion methods in MeOH, MeCN/iPr_2O system for **6** and MeOH/tBuOMe for **11**. To increase yield of compound **11** the reaction should be carried out in 1:2 ratio (metal : ligand). Compounds (**8** - red) and (**10** - brown) were obtained from reaction (**8**) by recrystallization of crude product via slow diffusion methods in MeOH, MeCN/iPr_2O system, mixture of brown and red crystals were separated manually. To increase yield of compound **8** the reaction should be carried out in 2:1 ratio (metal : ligand).

Complexes (**9**, **12 - 13**) were prepared in the same molar ratio of the ligand to the appropriate metal salt 2:1. To a solution of ligand H_2**L**[2] (64.5 mg, 0.20 mmol) the appropriate metal salt was added (0.10 mmol) ($Co(ClO_4)_2 \cdot 6H_2O$ – **9**, $Zn(CF_3SO_3)_2$ – **12**, $Cd(ClO_4)_2 \cdot 6H_2O$ – **13**) in 15 ml of MeOH. For complexes (**11 - 12**) yellow solution formed instantly and then triethylamine (0.20 mmol) was added. The color of the solutions has changed to a more intense one and the reaction mixtures were stirred for 24 h at room temperature. For complex (**9**) brown solution formed instantly with visible brown precipitate. To the reaction mixture 1 ml of 30% H_2O_2 was added and reaction was stirred for 24 h at room temperature, which resulted in formation of clear solution. After evaporation of solvent under reduced pressure, the residues were dissolved in minimum volume of MeOH and precipitated by excess of Et_2O. Brown (**9**) and yellow (**12 - 13**), solids were filtered via suction filtration and dried in the vacuum.

Complex (**1**) $Cd_2(H_2\mathbf{L}^{1\text{-}O})_2](ClO_4)_2$

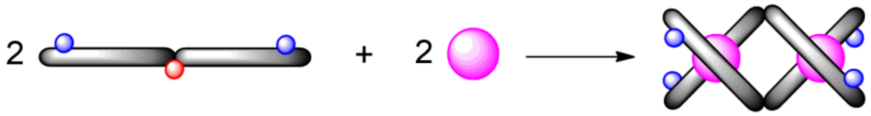

Yield: 124.3 mg, 87% based on ligand. Crystal suitable for X-ray analysis were obtained via slow diffusion methods in MeOH, MeCn/iPr_2O system. IR (KBr, cm^{-1}): $\nu_{broad}(O\text{-}H)_{methanol}$ 3467; ν(N-H) 3196 ; $\nu(C\text{-}H)_{ar}$ 3070; ν_{as} (CH_3) 2960; ν_s (CH_3) 2865; $\nu(C{=}C)_{ar}$ 1659, 1592; ν(C=N) 1477, 1380, 1377; ν(C-O) 1275; δ(OClO) 1162; $\gamma(C\text{-}H)_{ar}$ 1028, 952, 929, 829, 756, 734, 635, 574, 516, 432; γ(ClO) 641. ESI-MS(+) m/z (%):606 (100) $[Cd_2(H_2\mathbf{L}^{1\text{-}O})_2]^{2+}$, 1311 (15) $[Cd_2(H_2\mathbf{L}^{1\text{-}O})_2(ClO_4)]^{+}$. Microanalysis was not carried out due to the explosive properties of perchlorates.

Complex (**2**) $[Mn_2(H_2\mathbf{L}^{1\text{-}O})_2](NO_3)_2 \cdot 9\ H_2O$

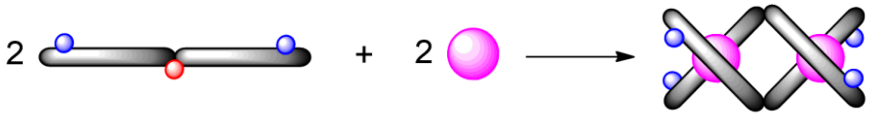

Yield: 119.0 mg, 85% based on ligand. Crystal suitable for X-ray analysis were obtained via slow diffusion methods in MeOH, MeCN/Et_2O system. IR (KBr, cm^{-1}): $\nu_{broad}(O\text{-}H)_{methanol}$ 3414; $\nu(C\text{-}H)_{ar}$ 3091, 3048; ν_{as} (CH_3) 2948; ν_s (CH_3) 2863; $\nu(C{=}C)_{ar}$ 1760, 1631, 1607, 1575; $\nu_{as}(NO_2)$ 1463; ν(C=N) 1383, 1325, 1279, 1216; ν(C-O) 1263; ν_s,broad(NO_2) 1279, 1242; ν(NO) 1051; $\gamma(C\text{-}H)_{ar}$ 1184, 1147, 1041, 1108, 1036, 1004, 944, 922, 899, 775, 656, 635, 564, 517, 496, 431; γ(NO) 821. ESI-MS(+) m/z (%):548 (70) $[Mn_2(H_2\mathbf{L}^{1\text{-}O})_2]^{2+}$, 1095 (10) $[Mn_2(H_2\mathbf{L}^{1\text{-}O})(H_1\mathbf{L}^{1\text{-}O})]^{+}$. Anal. calc. for $[Mn_2(H_2\mathbf{L}^{1\text{-}O})_2(NO_3)_2(H_2O)_9]$ (1385.21): C, 48.56; H, 5.68; N, 18.20; found: C, 48.96; H, 5.71; N, 18.23%.

Complex (**3**) $[Cd_2(H\mathbf{L}^1)_2]$

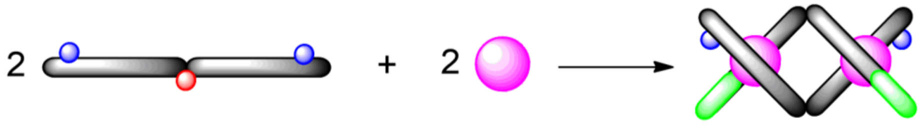

Yield: 99.4 mg, 81% based on ligand. Crystal suitable for X-ray analysis were obtained via slow diffusion methods in MeOH, MeCN/PhMe system. IR (KBr, cm^{-1}): ν_{broad}(O-H)$_{methanol}$ 3401; ν(C-H)$_{ar}$ 3049; ν_{as}(CH_3) 2956; ν_s (CH_3) 2865; ν(C=C)$_{ar}$ 1632, 1499, 1466; V_{as}(O-C-O) 1575; V_s(O-C-O) 1443; ν(C=N) 1410, 1377, 1332, 1244, 1216; ν(C-O) 1268; γ(C-H)$_{ar}$ 1192, 1151, 1108, 1059, 1040, 1005, 946, 901, 823, 773, 736, 633, 601, 560, 520; δ(O-C-O) 675. ESI-MS(+) m/z (%):606 (100) $[Cd_2(H_2\mathbf{L}^{\mathbf{1}})_2]^{2+}$**,** 1211 (15) $[Cd_2(H_2\mathbf{L}^{\mathbf{1}})(H\mathbf{L}^{\mathbf{1}})]^{+}$. Anal. calc. for $[Cd_2(H\mathbf{L}^{\mathbf{1}})_2]$ (1216.32): C, 55.40; H, 4.98; N, 18.46; found: C, 54.20; H, 4.51; N, 17.30%.

Complex (**4**) $[Mn_2(H\mathbf{L}^1)_2]$

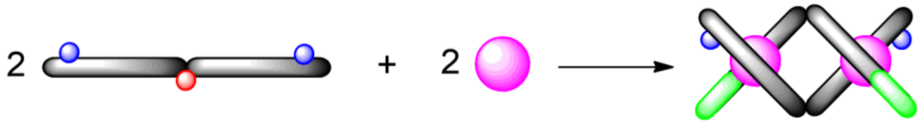

Yield: 88.9 mg, 80% based on ligand. Crystal suitable for X-ray analysis were obtained via slow diffusion methods in MeOH, MeCN/PhMe system. IR (KBr, cm^{-1}): ν_{broad}(O-H)$_{methanol}$ 3396; ν(C-H)$_{ar}$ 3041; ν_{as}(CH_3) 2956; ν_s (CH_3) 2865; ν(C=C)$_{ar}$ 1626, 1615, 1489, 1461; V_{as}(O-C-O) 1578; V_s(O-C-O) 1441; ν(C=N) 1359, 1326, 1244; ν(C-O) 1273; γ(C-H)$_{ar}$ 1182, 1145, 1106, 1059, 1033, 1004, 942, 901, 822, 772, 739, 622, 563, 521; δ(O-C-O) 661. ESI-MS(+) m/z (%):548 (100) $[Mn_2(H_2\mathbf{L}^{\mathbf{1}})_2]^{2+}$**,** 1095 (20) $[Mn_2(H_2\mathbf{L}^{\mathbf{1}})(H\mathbf{L}^{\mathbf{1}})]^{+}$. Anal. calc. for $[Mn_2(H\mathbf{L}^1)_2]$ (1098.38): C, 55.9; H, 5.79; N, 17.38; found: C, 52.51; H, 5.43; N, 15.77%.

Complex (**5**) $Zn(H_2\mathbf{L}^{1\text{-}O})(H_3\mathbf{L}^{\mathbf{1\text{-}NH}})Zn(H_2O)](OTf)_3$

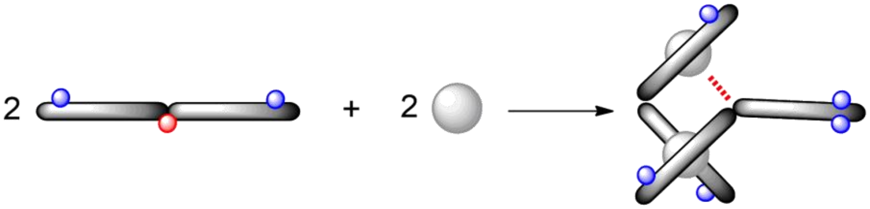

Yield: 134.7 mg, 85% based on ligand. Crystal suitable for X-ray analysis were obtained via slow diffusion methods in MeOH, MeCN/iPr_2O system. IR (KBr, cm^{-1}): ν(N-H) 3286; ν(C-H)$_{ar}$ 3054; ν_{as}(CH_3) 2947(m); ν_s (CH_3) 2867; ν(C=C)$_{ar}$: 1646, 1629, 1574; ν(C=N) 1467, 1372; ν_{as}(SO_3); 1331; ν_{as}(CF_3) 1283, 1248; ν_s(CF_3); 1122; ν_s(SO_3) 1031; γ(C-H)$_{ar}$ 1004, 939, 822, 737, 625, 558, 517, 491, 428. ESI-MS(+) m/z (%):559 (100) $[Zn_2(H_2\mathbf{L}^{\mathbf{1\text{-}O}})(H_2\mathbf{L}^{\mathbf{1\text{-}NH}})]^{2+}$**,** 1267 (15) $[Zn_2(H_2\mathbf{L}^{\mathbf{1\text{-}O}})(H_2\mathbf{L}^{\mathbf{1\text{-}NH}})(CF_3SO_3)]^{+}$. Anal. calc. for $Zn(H_2\mathbf{L}^{\mathbf{1\text{-}O}})(H_3\mathbf{L}^{\mathbf{1\text{-}NH}})Zn(H_2O)](OTf)_3 \cdot 3\ H_2O$ (1639.21): C, 43.23; H, 4.18; N, 13.67; found: C, 43.43; H, 4.53; N, 13.52%.

Complex (**6**) $[Ni_2(H_3\mathbf{L}^{\mathbf{1\text{-}NH}})_2(MeOH)_2(MeCN)_2](ClO_4)_4$

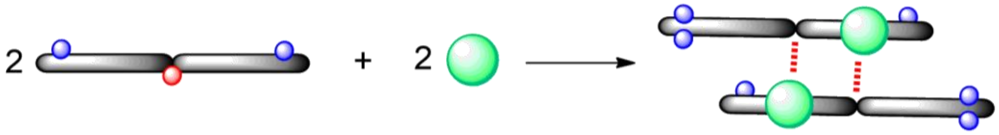

Yield: 125.1 mg, 75% based on ligand. Crystal suitable for X-ray analysis were obtained via slow diffusion methods in MeOH, MeCN/tBuOMe system. IR (KBr, cm^{-1}): ν_{broad}(O-H)$_{methanol}$ 3381; ν(C-H)$_{ar}$ 3097, 3056; ν_{as} (CH_3) 2961; ν_s (CH_3) 2860; ν(C=C)$_{ar}$ 1661, 1629, 1581; ν(C=N) 1467, 1395, 1358, 1321, 1289, 1233; ν(C-O) 1257; δ(OClO) 1120, 1084; γ(C-H)$_{ar}$ 1041, 1002, 948, 922, 821, 739, 526, 430; γ(ClO) 625. ESI-MS(+) m/z (%): 551 (20) $[Ni_2(H_2\mathbf{L}^{\mathbf{1\text{-}NH}})_2]^{+}$**,** 651 (10) $[Ni(H_2\mathbf{L}^{\mathbf{1\text{-}NH}})_2(ClO_4)]^{+}$. Microanalysis was not carried out due to the explosive properties of perchlorates.

Complex (**7**) $[Fe(H_3\mathbf{L}^{\mathbf{1\text{-}OH}})Cl_2][FeCl_4]$

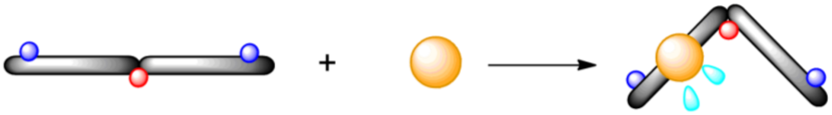

Yield: 71.2 mg, 86% based on ligand. Crystal suitable for X-ray analysis were obtained via slow diffusion methods in MeOH, MeCn/PhMe system. IR (KBr, cm^{-1}): ν(N-H) 3282; ν(C-H)$_{ar}$ 3044; ν$_{as}$(CH_3) 2960; ν$_s$ (CH_3) 2850; ν(C=C)$_{ar}$ 1653, 1608, 1590, 1541, 1474, 1464; ν(C=N) 1361, 1329, 1283, 1226; ν(C-O) 1257; γ(C-H)$_{ar}$ 1185, 1135, 1046, 1026, 1002, 966, 956, 924, 901, 854, 824, 809, 747, 675, 622, 611, 594, 542, 525. ESI-MS(+) m/z (%):548 (60) [Fe(H**L**$^{\mathbf{1\text{-}OH}}$)]$^+$**,** 584 (30) [Fe(H_2**L**$^{\mathbf{1\text{-}OH}}$)Cl]$^+$. Anal. calc. for [Fe(H_3**L**$^{\mathbf{1\text{-}OH}}$)Cl_2[$FeCl_4$](MeOH)$_2$(MeCN)$_2$] (965.19): C, 42.31; H, 4.59; N, 14.51; found: C, 43.53; H, 5.05; N, 14.25%.

Complex (**8**) [Co_4(H_2**L**$^{\mathbf{1\text{-}O}}$)$_2$(OH)$_2$(H_2O)$_2$(MeCN)(MeOH)](ClO_4)$_4$

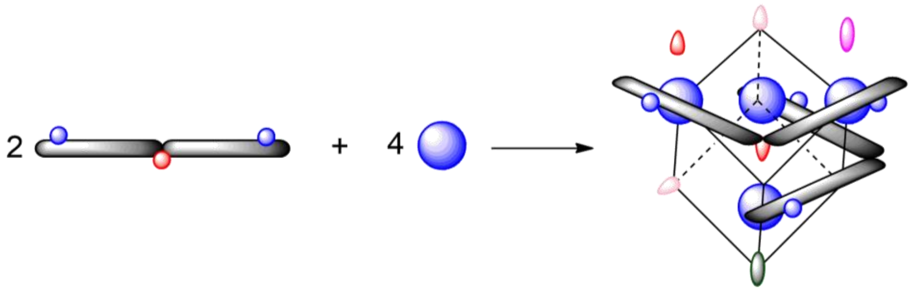

Yield: 47.9 mg, 28% based on ligand. Crystal suitable for X-ray analysis were obtained via slow diffusion methods in MeOH, MeCN/iPr$_2$O system. IR (KBr, cm^{-1}): ν$_{broad}$(O-H)$_{methanol}$ 3434; ν(N-H) 3140 ; ν(C-H)$_{ar}$ 3039; ν$_{as}$(CH_3) 2950; ν$_s$ (CH_3) 2863 ν(C=C)$_{ar}$ 1653, 1611, 1560; ν(C=N) 1488, 1445, 1361, 1227; ν(C-O) 1272; δ(OClO) 1158, 1089; γ(C-H)$_{ar}$ 1052, 1010, 973, 931, 826, 741, 686, 535; γ(ClO) 624. ESI-MS(+) m/z (%): 523 (48) [Co(H_3**L**$^{\mathbf{1\text{-}O}}$)$_2$]$^{2+}$; 1045 (100) [Co(H_2**L**$^{\mathbf{1\text{-}O}}$)$_2$]$^+$. Cubic complex was instable in ESI-MS conditions, so we were observed only peaks from defragmentation of the complex. Microanalysis was not carried out due to the explosive properties of perchlorates.

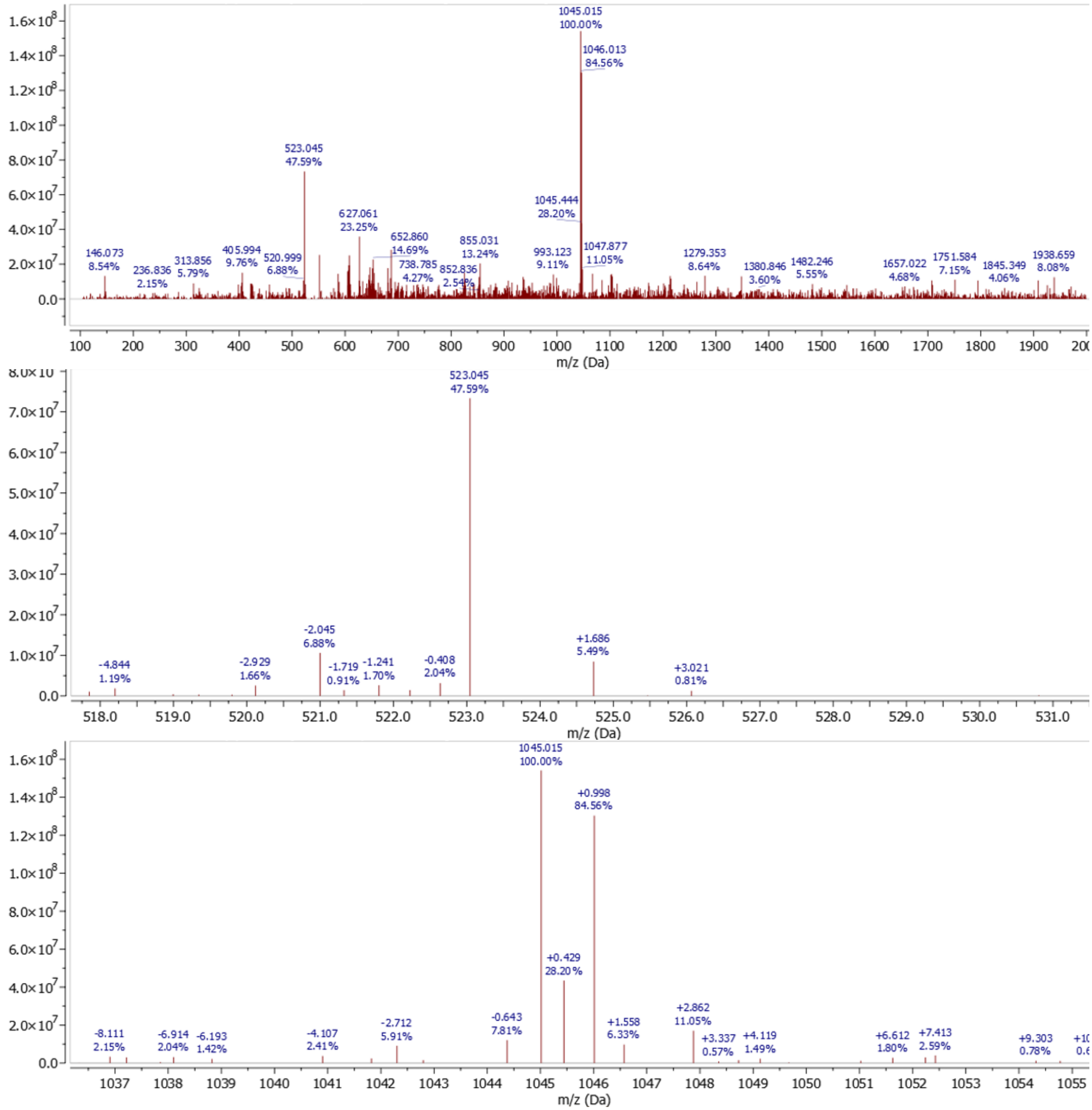

Spectrum 3. ESI-MS spectra of **8**.

Complex (**9**) $[Co(HL^2)_2](ClO_4)$

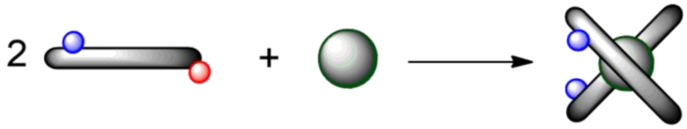

Yield: 107.1 mg, 86% based on ligand. Crystal suitable for X-ray analysis were obtained via slow diffusion methods in MeOH/Et_2O system. IR (KBr, cm^{-1}): ν_{broad}(O-H)$_{methanol}$ 3391; ν(N-H) 3144 ; ν(C-H)$_{ar}$ 3048, 3023; ν_{as}(CH_3) 2954; ν_s (CH_3) 2861 ν(C=C)$_{ar}$ 1611, 1533; ν(C=N) 1480, 1463, 1362, 1259, 1214; ν(C-O) 1274; δ(OClO) 1178, 1149; γ(C-H)$_{ar}$ 1107, 1047, 1009, 975, 909, 832, 735, 681; γ(ClO) 615. ESI-MS(+) m/z (%): 701 (100) $[Co(H_2L^2)_2]^+$. Microanalysis was not carried out due to the explosive properties of perchlorates. ^{1}HNMR (400 MHz, CD_3CN): δ = 1.24 (s, 9H), 4.16 (s, 3H), 6.58 (d, 1H), 6.86 (m, 1H), 7.01 (m, 2H), 7.10 (dd, 1H), 7.22 (m, 1H), 7.52 (d, 1H), 8.42 (s, 1H) ppm.

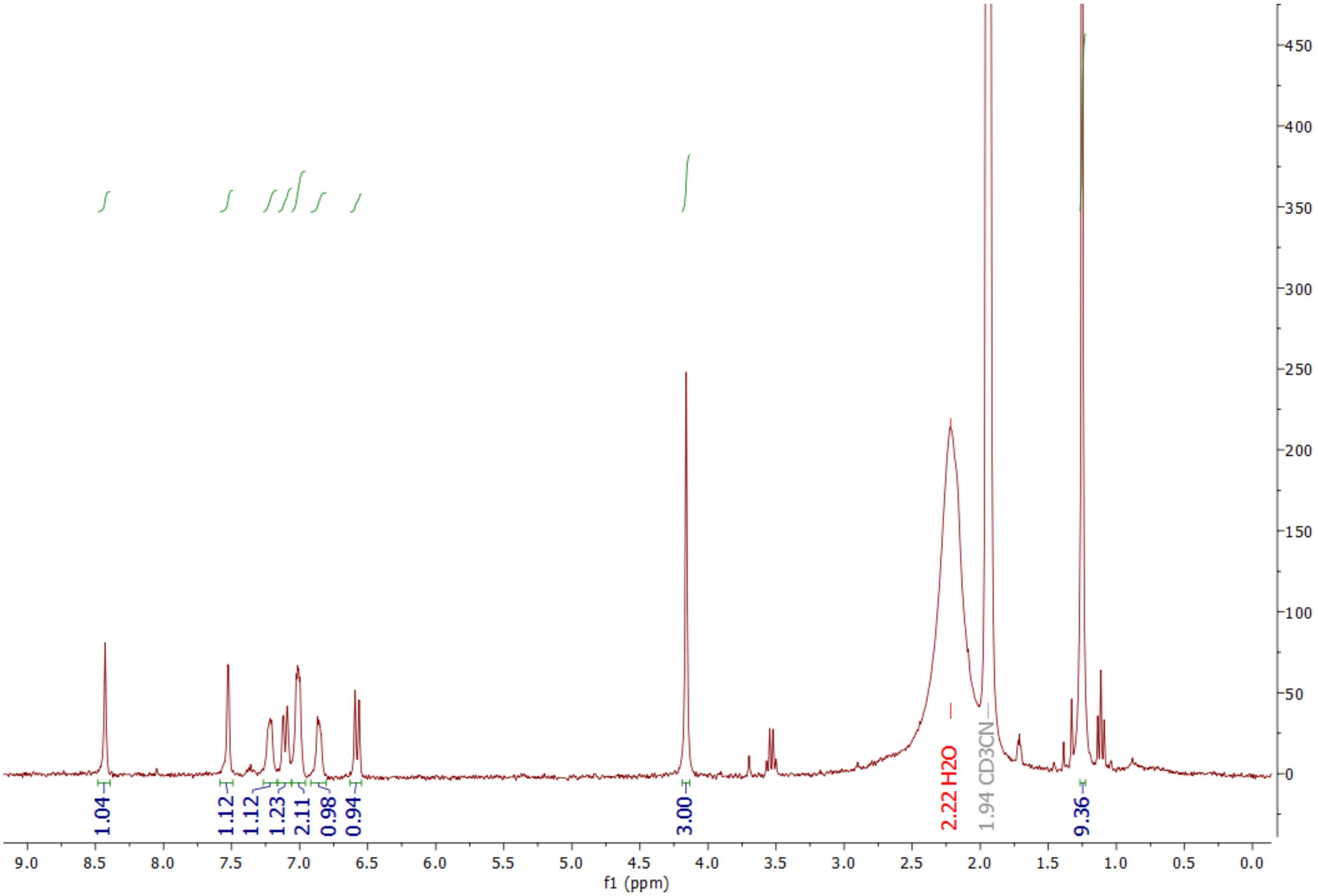

Spectrum 4. ^{1}H NMR of **9**.

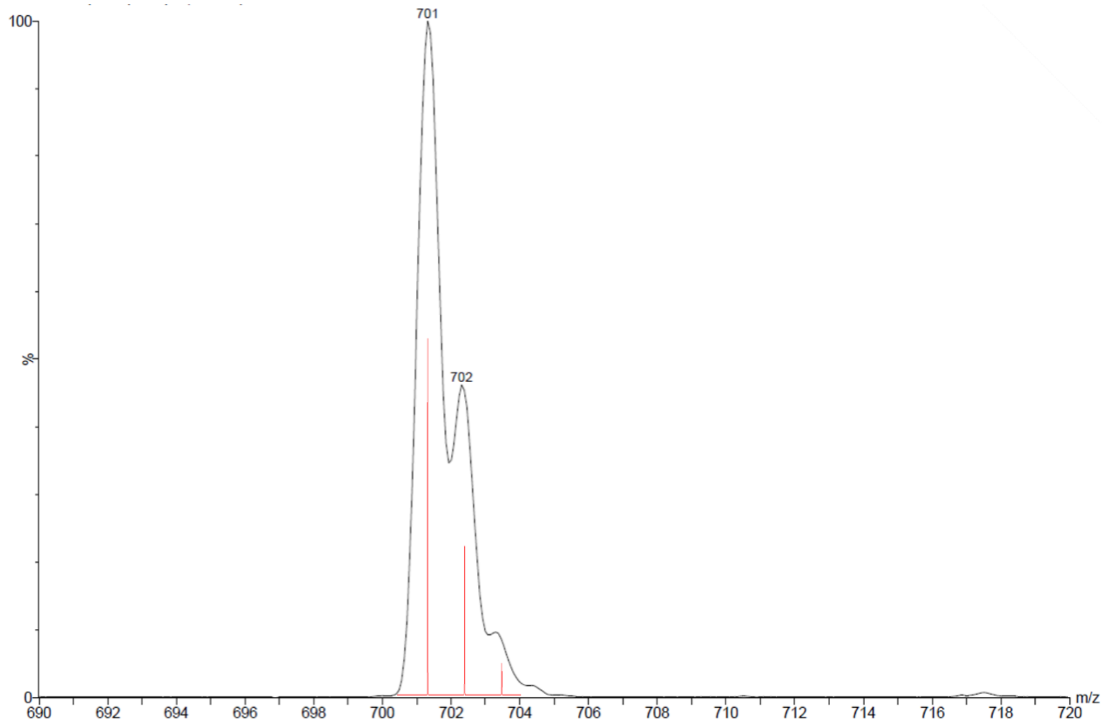

Spectrum 5. ESI-MS spectrum of **9** with theoretical prediction in red.

Complex (**10**) [Co(H$_3$**L**$^{\mathbf{1\text{-}NH}}$)$_2$](ClO$_4$)$_3$(MeOH)$_2$(H$_2$O)$_3$

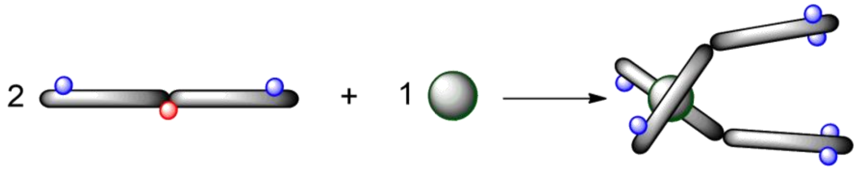

Yield: 58.5 mg, 43% based on ligand. Crystal suitable for X-ray analysis were obtained via slow diffusion methods in MeOH, MeCN/iPr$_2$O system. IR (KBr, cm^{-1}): ν_{broad}(O-H)$_{methanol}$ 3434; ν(N-H) 3140 ; ν(C-H)$_{ar}$ 3039; ν_{as}(CH$_3$) 2950; ν_s (CH$_3$) 2863 ν(C=C)$_{ar}$ 1653, 1611, 1560; ν(C=N) 1488, 1445, 1361, 1227; ν(C-O) 1272; δ(OClO) 1158, 1089; γ(C-H)$_{ar}$ 1052, 1010, 973, 931, 826, 741, 686, 535; γ(ClO) 624. ESI-MS(+) m/z (%):1143 (100) [Co(H$_3$**L**$^{\mathbf{1\text{-}NH}}$)(H$_2$**L**$^{\mathbf{1\text{-}NH}}$)(ClO$_4$)]$^+$; 1043 (50) [Co(H$_2$**L**$^{\mathbf{1\text{-}NH}}$)$_2$]$^+$. Microanalysis was not carried out due to the explosive properties of perchlorates.

Complex (**11**) [Ni(H$_3$**L**$^{\mathbf{1\text{-}NH}}$)$_2$](ClO$_4$)$_2$(MeOH)$_3$

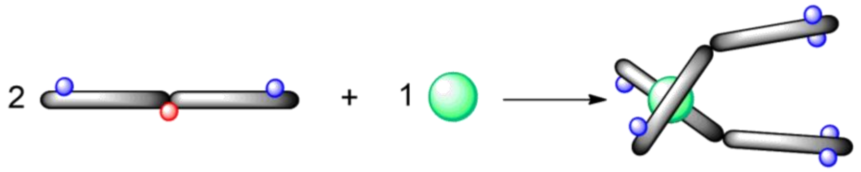

Yield: 78.8 mg, 68% based on ligand. Crystal suitable for X-ray analysis were obtained via slow diffusion methods in MeOH, MeCN/tBuOMe system. IR (KBr, cm^{-1}): ν_{broad}(O-H)$_{methanol}$ 3474; ν(N-H) 3158 ; ν(C-H)$_{ar}$ 3094, 3051; ν_{as}(CH$_3$) 2953; ν_s (CH$_3$) 2862 ν(C=C)$_{ar}$ 1630, 1599; ν(C=N) 1467, 1439, 1378, 1361, 1217, 1146; ν(C-O) 1274; δ(OClO) 1187, 1120; γ(C-H)$_{ar}$ 1044, 1005, 931, 897, 823, 758, 736, 665, 608, 530, 483, 431; γ(ClO) 628. ESI-MS(+) m/z (%): 551 (18) [Ni(H$_2$**L**$^{\mathbf{1\text{-}NH}}$)]$^+$, 1045 (10) [Ni$_2$(H$_2$**L**$^{\mathbf{1\text{-}NH}}$)(H$_3$**L**$^{\mathbf{1\text{-}NH}}$)]$^+$. Microanalysis was not carried out due to the explosive properties of perchlorates.

Complex (**12**) [Zn(H**L**^{2})$_2$]

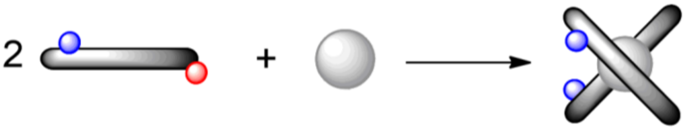

Yield: 87.0 mg, 79% based on ligand. IR (KBr, cm^{-1}): ν(C-H)$_{ar}$ 3035; ν_{as} (CH_3) 2964; ν_s (CH_3) 2902, 2864; ν(C=C)$_{ar}$: 1627, 1612, 1571, ν(C=N) 1464, 1431, 1376, 1360, ν_{as}(SO_3); 1306; ν_{as}(CF_3) 1269, 1254; ν_s(CF_3); 1180; ν_s(SO_3) 1026(s), γ(C-H)$_{ar}$ 1005(s), 938, 871, 846, 826, 757, 737, 675, 638, 614, 583, 513, 433. ESI-MS(+) m/z (%):321 (100) $[H_4\mathbf{L}^2]^+$, 301 (10) $[Zn_2(H_2\mathbf{L}^2)(CF_3SO_3^-)]^{2+}$. Anal. calc. for $[Zn(H_2\mathbf{L}^2)_2]\cdot 3H_2O$ (762.22): C, 59.88; H, 6.35; N, 14.70; found: C, 59.06; H, 5.64; N, 14.40%. ^{1}HNMR (400 MHz, d_6 -DMSO): δ = 1.29 (d, 9H), 3.61 (d, 3H), 6.96 (m, 6.5H), 7.88 (s, 1H), 8.06 (s, 0.5H) ppm.

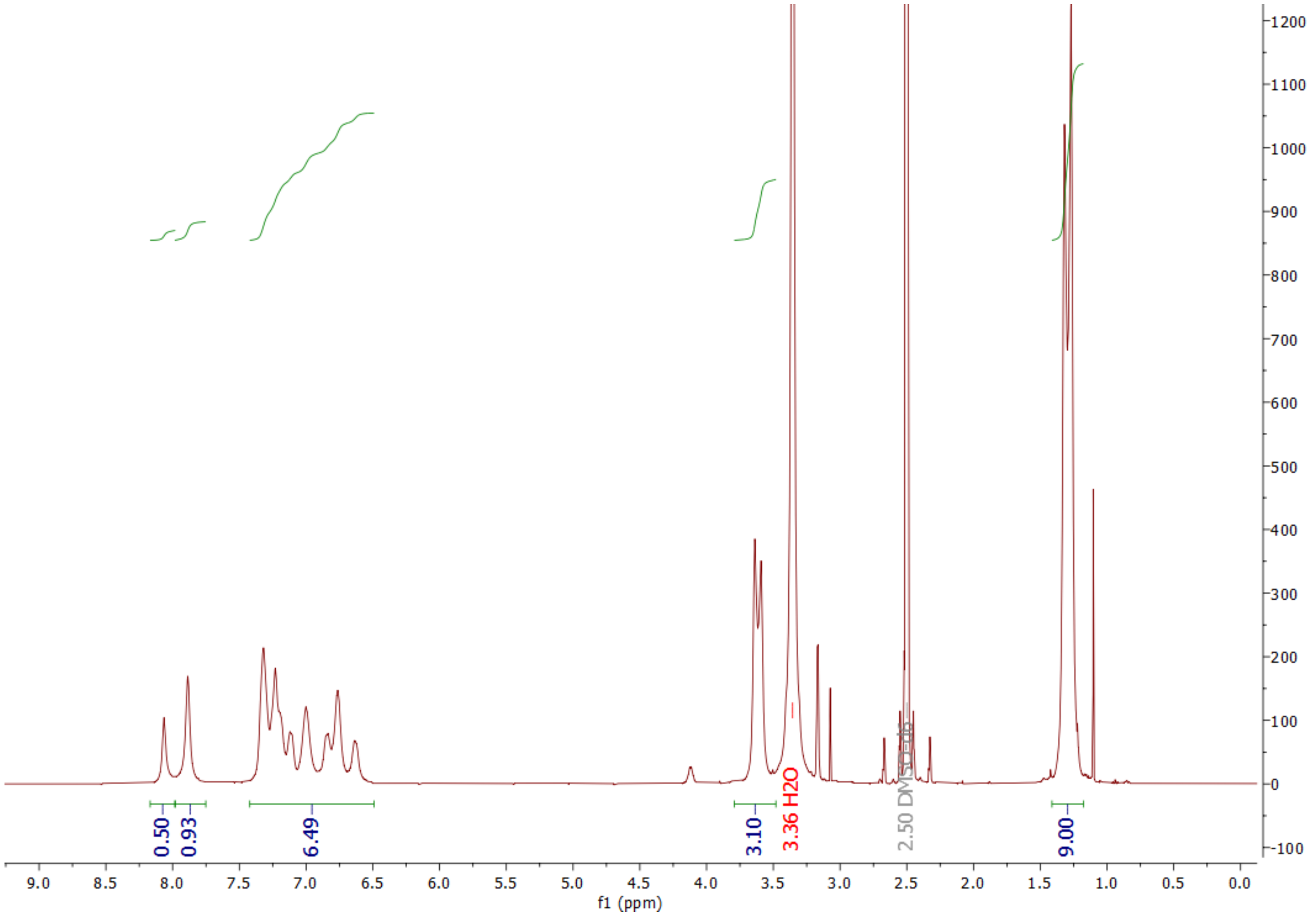

Spectrum 6. ^{1}H NMR of **12**.

Complex (**13**) $[Cd(H\mathbf{L}^2)_2]$

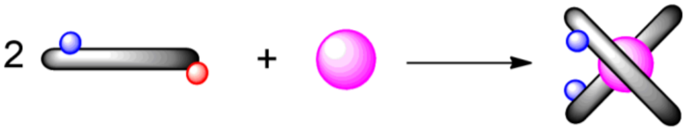

Yield: 88.1 mg, 75% based on ligand. IR (KBr, cm^{-1}): ν(N-H) 3275; ν(C-H)$_{ar}$ 3059, 3024; ν_{as} (CH_3) 2962; ν_s (CH_3) 2906, 2869; ν(C=C)$_{ar}$ 1630(s), 1609, 1578; ν(C=N) 1488, 1471, 1431, 1394, 1372, 1362, 1291, 1222, 1183; ν(C-O) 1261; δ(OClO) 1121, 1073; γ(C-H)$_{ar}$ 1040, 1006, 957, 946, 920, 905, 824, 805, 756, 738; γ(ClO) 625. ESI-MS(+) m/z (%):321 (50) $[H_4\mathbf{L}^2]^+$, 421 (40) $[(H_5\mathbf{L}^2)(ClO_4^-)]^+$, 645 (10) $[Cd_2(H\mathbf{L}^2)ClO_4]^+$. Microanalysis was not carried out due to the explosive properties of perchlorates. ^{1}HNMR (400 MHz, d_6 -DMSO): δ = 1.33 (m, 10H), 3.63 (s, 3H), 6.55 (s, 0.21H), 7.16 (m, 7H), 7.89 (d, 0.73H), 8.06(d, 1.14H) ppm.

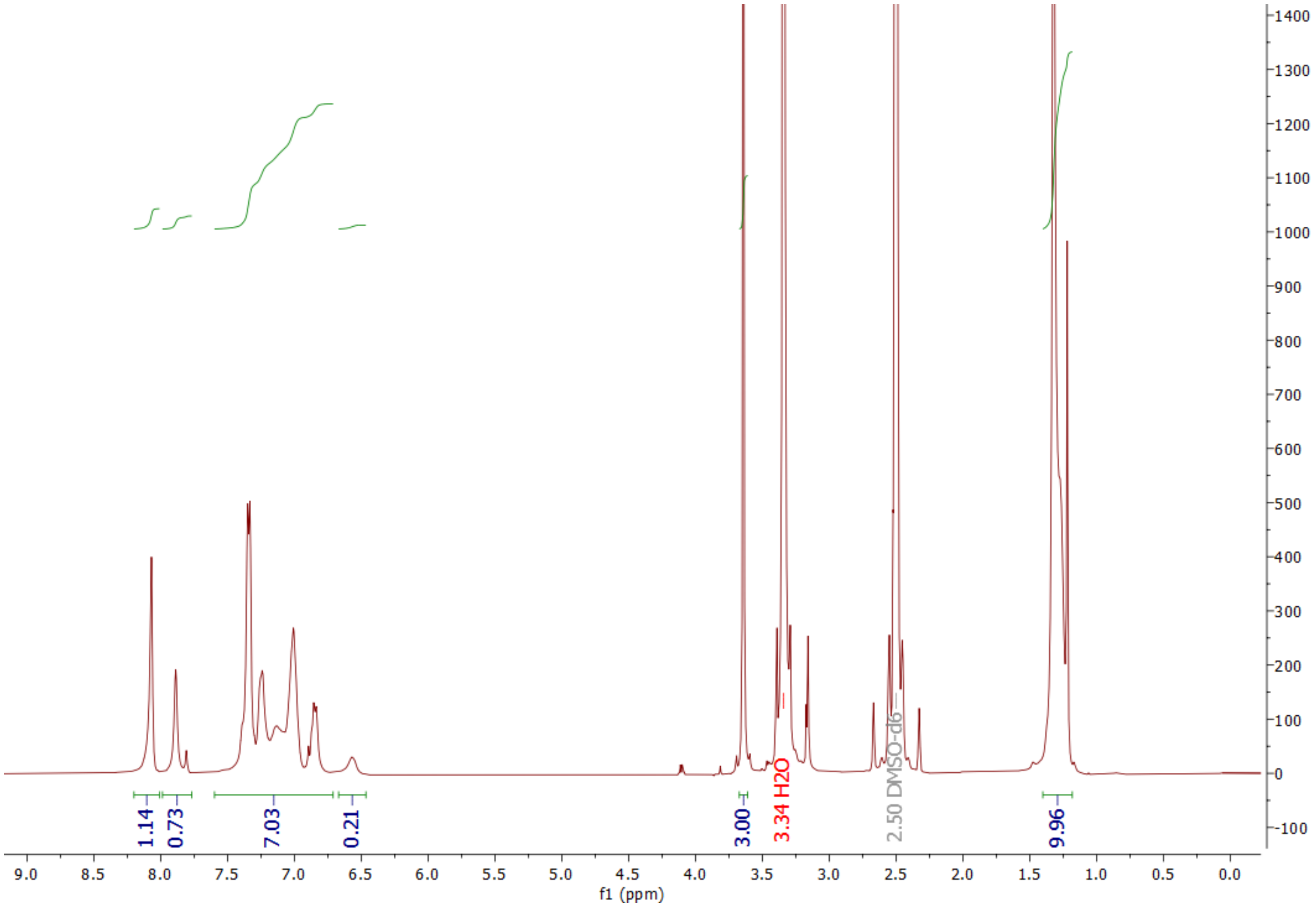

Spectrum 7. ^{1}H NMR of **13**.

III. Crystallographic data

X-ray crystallography Diffraction data were collected by the ω-scan technique, for **2**, **3**, **7**, **9** at 100(1) K, for **8** and **10** at 130(1) K, and for **4** and **6** at room temperature, on Agilent Technologies Xcalibur four-circle diffractometer with Eos CCD detector and graphite-monochromated MoK_{α} radiation (λ=0.71069 Å), and for **1**, **5** and **11** at 130(1) K and for H_3**L**^{1} at room temperature on Agilent Technologies SuperNova four-circle diffractometer with Atlas CCD detector and mirror-monochromated CuK_{α} radiation (λ=1.54178 Å). The data were corrected for Lorentz-polarization as well as for absorption effects.[4] Precise unit-cell parameters were determined by a least-squares fit of reflections of the highest intensity, chosen from the whole experiment. The structures were solved with SIR92[5] and refined with the full-matrix least-squares procedure on F^2 by SHELXL-2013.[6] All non-hydrogen atoms were refined anisotropically, hydrogen atoms were placed in idealized positions and refined as 'riding model' with isotropic displacement parameters set at 1.2 (1.5 for methyl or hydroxyl groups) times U_{eq} of appropriate carrier atoms. Positions of those hydrogen which cannot be reasonably placed by this procedure (water molecules, some hydroxyl groups) were calculated according to potential hydrogen bonds. The crystals of H_3**L**^{1} and **1** turned out to be twinned, (which was taken into account during both data reduction and refinement); the BASF factor, describing the content of one of the component[6] refined at 79.0(3)% for H_3**L**^{1} and at 78.0(7)% for **1**.

In almost all structures the solvent molecules were found. Additionally, in some of them (**2**, **5**, **7**, **8**, **10**, **11**) the large voids filled with diffused electron density were found; as the modellings of solvent molecules were in these cases unsuccessful, the SQUEEZE procedure[7] was applied.

Crystal data: $[H_5L^1]^{2+}$: $2(C_{28}H_{32}N_8O)^{2+}\cdot 4(ClO_4)^{-}\cdot C_7H_8\cdot CH_3OH$, M_r=1515.21, triclinic, P-1, a=11.4426(5) Å, b=13.0011(5) Å, c= 24.5126(10) Å, α= 90.991(3)°, β= 91.953(4)°, γ= 92.005(4)°, V= 3641.7(3) Å^3, Z=2, d_x=1.38 g·cm^{-3}, μ=2.16 mm^{-1}, F(000)=1584. 25627 reflections collected up to 2Θ = 67.5°, 12871 symmetry independent, (R_{int}=8.60%), 7644 with I>2σ(I). Final R[I>2σ(I)]=10.20%, wR2[I>2σ(I)]=23.13%, R[all refl.]=14.69%, wR2[all refl.]=26.03%, S=1.05, max/min Δρ = 1.52/-0.58 e·Å^{-3}.

1: $C_{56}H_{58}Cd_2N_{16}O_2\cdot 2(ClO_4)$, M_r=1410.90, monoclinic, C2/c, a = 24.6186(15)Å, b = 11.8872(6) Å, c= 25.2254(19) Å, β= 117.853(9)°, V= 6526.9(9) Å^3, Z=4, d_x=1.44 g·cm^{-3}, μ=6.52 mm^{-1}, F(000)=2864. 48493 reflections collected up to 2Θ = 75.5°, 6604 symmetry independent (R_{int}=8.60%), 6302 with I>2σ(I). Final R[I>2σ(I)]=10.59%, wR2[I>2σ(I)]=28.70%, R[all refl.]=11.03%, wR2[all refl.]=29.02%, S=1.08, max/min Δρ = 2.91/-1.81 e·Å^{-3}.

2: $C_{56}H_{58}Mn_2N_{16}O_2\cdot 2(NO_3)$, M_r=1221.08, monoclinic, C2/c, a= 22.6232(16) Å, b= 23.8721(19) Å, c= 11.7805(11) Å, β= 101.465(8)°, V= 6235.3(9)Å^3, Z=4, d_x=1.30 g·cm^{-3}, μ=0.47 mm^{-1}, F(000)=2536. 13702 reflections collected up to 2Θ = 50°, 5495 symmetry independent (R_{int}=6.46%), 3285 with I>2σ(I). Final R[I>2σ(I)]=7.85%, wR2[I>2σ(I)]=19.56%, R[all refl.]=12.78%, wR2[all refl.]=21.76%, S=1.13, max/min Δρ = 1.02/-0.56 e·Å^{-3}.

3: $C_{56}H_{56}Cd_2N_{16}O_{12}\cdot 4(C_7H_8)\cdot 2(CH_3OH)$, M_r=1642.58, monoclinic, C2/c, a= 26.597(3) Å, b= 10.8810(8) Å, c= 28.386(2) Å, β= 102.252(10)°, V= 8027.9(13) Å^3, Z=4, d_x=1.36 g·cm^{-3}, μ=0.59 mm^{-1}, F(000)=3408. 17410 reflections collected up to 2Θ = 28.3°, 8384 symmetry independent (R_{int}=4.21%), 6602 with I>2σ(I). Final R[I>2σ(I)]=6.08%, wR2[I>2σ(I)]=15.90%, R[all refl.]=7.83%, wR2[all refl.]=17.46%, S=1.04, max/min Δρ = 2.73/-1.84 e·Å^{-3}.

4: $C_{56}H_{56}Mn_2N_{16}O_2\cdot 4(C_7H_8)\cdot 2(CH_3OH)$, M_r=1527.66, monoclinic, C2/c, a= 26.9331(16) Å, b= 10.5896(6) Å, c= 28.319(3) Å, β= 103.216(7)°, V= 7863.0(11) Å^3, Z=4, d_x=1.29 g·cm^{-3}, μ=0.38 mm^{-1}, F(000)=3224. 15194 reflections collected up to 2Θ = 25°, 6904 symmetry independent (R_{int}=3.17%), 5073 with I>2σ(I). Final R[I>2σ(I)]=5.58%, wR2[I>2σ(I)]=15.58%, R[all refl.]=8.42%, wR2[all refl.]=16.80%, S=1.12, max/min Δρ = 1.36/-0.60 e·Å^{-3}.

5: $C_{56}H_{59}N_{16}O_3Zn_2\cdot 3(CF_3SO_3)\cdot C_6H_{14}O\cdot CH_3OH$, M_r=1716.40, triclinic, P-1, a = 13.0126(3) Å, b = 14.8306(4) Å, c = 22.5212(5) Å, α = 85.340(2)°, β = 81.718(2)°, γ = 76.453(2)°, V = 4176.16(18) Å^3, Z=2, d_x=1.37 g·cm^{-3}, μ=2.15 mm^{-1}, F(000)=1772. 40230 reflections collected up to 2Θ = 67.5°, 14994 symmetry independent (R_{int}=3.95%), 13218 with I>2σ(I). Final R[I>2σ(I)]=14.65%, wR2[I>2σ(I)]=32.07%, R[all refl.]=15.48%, wR2[all refl.]=32.48%, S=1.04, max/min Δρ = 3.84/-3.35 e·Å^{-3}.

6: $C_{62}H_{72}N_{18}Ni_2O_4\cdot 4(ClO_4)\cdot 4(CH_3OH)$, M_r=1776.76, monoclinic, $P2_1/c$, a= 18.1687(4) Å, b= 24.4309(6) Å, c= 19.1441(4) Å, β= 111.448(3)°, V= 7909.2(3) Å^3, Z=4, d_x=1.49 g·cm^{-3}, μ=0.70 mm^{-1}, F(000)=3704. 33065 reflections collected up to 2Θ = 26.67°, 15279 symmetry independent (R_{int}=3.51%), 10196 with I>2σ(I). Final R[I>2σ(I)]=6.72%, wR2[I>2σ(I)]=16.29%, R[all refl.]=11.14%, wR2[all refl.]=18.90%, S=1.03, max/min Δρ = 1.85/-1.10 e·Å^{-3}.

7: $C_{28}H_{29}Cl_2FeN_8O\cdot Cl_4Fe\cdot CH_3CN$, M_r=900.10, triclinic, P-1, a = 12.0163(16) Å, b = 12.469(2) Å, c = 15.283(3) Å, α = 110.832(18)°, β = 106.727(15)°, γ = 101.067(13)°, V = 1935.7(6) Å^3, Z=2, d_x=1.54 g·cm^{-3}, μ=1.21 mm^{-1}, F(000)=918.

13535 reflections collected up to 2Θ = 25°, 6810 symmetry independent (R_{int}=8.50%), 3300 with I>2σ(I). Final R[I>2σ(I)]=8.99%, wR2[I>2σ(I)]=17.42%, R[all refl.]=18.10%, wR2[all refl.]=21.15%, S=1.06, max/min Δρ = 1.18/-0.79 e·Å^{-3}.

8: $C_{59}H_{68}Co_4N_{17}O_7 \cdot 4(ClO_4) \cdot CH_3OH \cdot H_2O$, M_r=1810.88, monoclinic, P2$_1$/n, a = 11.8757(8) Å, b = 29.4996(19) Å, c = 23.369(4) Å, β = 102.610(12)°, V = 7989.2(15) Å^3, Z=4, d_x=1.51 g·cm^{-3}, μ=1.03 mm^{-1}, F(000)=3716. 33667 reflections collected up to 2Θ = 25°, 14030 symmetry independent (R_{int}=8.59%), 7973 with I>2σ(I). Final R[I>2σ(I)]=11.48%, wR2[I>2σ(I)]=27.66%, R[all refl.]=16.49%, wR2[all refl.]=30.06%, S=1.34, max/min Δρ = 2.09/-0.92 e·Å^{-3}.

9: $C_{38}H_{42}CoN_8O_2 \cdot (ClO_4) \cdot CH_3OH$, M_r=923.33, monoclinic, P2$_1$/c, a = 14.5982(6) Å, b = 13.8618(8) Å, c = 23.7026(10) Å, α = 90°, β = 106.384(4)°, γ = 90°, V = 4601.6(4) Å^3, Z=4, d_x=1.33 g·cm^{-3}, μ=0.493 mm^{-1}, F(000)=1944. 19246 reflections collected up to 2Θ = 25°, 9852 symmetry independent (R_{int}=8.50%), 6080 with I>2σ(I). Final R[I>2σ(I)]=8.68%, wR2[I>2σ(I)]=17.39%, R[all refl.]=14.26%, wR2[all refl.]=20.88%, S=1.04, max/min Δρ = 1.52/-1.05 e·Å^{-3}.

10: $C_{56}H_{57}CoN_{16}O_2 \cdot 3(ClO_4) \cdot 3(H_2O) \cdot 2(CH_3OH)$, M_r=1461.58, triclinic, P-1, a = 14.9788(10) Å, b = 15.0390(10) Å, c = 20.7602(11) Å, α = 99.992(5)°, β = 91.101(5)°, γ = 110.153(6)°, V = 4307.8(5) Å^3, Z=2, d_x=1.13 g·cm^{-3}, μ=0.36 mm^{-1}, F(000)=1522. 25520 reflections collected up to 2Θ = 25°, 15045 symmetry independent (R_{int}=6.60%), 5918 with I>2σ(I). Final R[I>2σ(I)]=11.53%, wR2[I>2σ(I)]=26.15%, R[all refl.]=20.80%, wR2[all refl.]=29.15%, S=1.09, max/min Δρ = 1.43/-0.93 e·Å^{-3}.

11: $C_{56}H_{56}N_{16}NiO_2 \cdot 2ClO_4 \cdot 2(CH_3OH)$, M_r=1308.86, hexagonal, P6$_1$22, a= 14.2268(5) Å, 57.828(4), V= 10136.3(12) Å^3, Z=6, d_x=1.22 g·cm^{-3}, μ=1.29 mm^{-1}, F(000)=3906. 42204 reflections collected up to 2Θ = 67.5°, 6088 symmetry independent (R_{int}=8.95%), 5452 with I>2σ(I). Final R[I>2σ(I)]=8.77%, wR2[I>2σ(I)]=21.24%, R[all refl.]=8.77%, wR2[all refl.]=21.68%, S=1.13, max/min Δρ = 0.77/-0.28 e·Å^{-3}.

Crystallographic data (excluding structure factors) for the structural analysis has been deposited with the Cambridge Crystallographic Data Centre, Nos. CCDC-1482453-1482463 for compounds [H_5**L**^{1}]$(ClO_4)_2$, **1** – **11**. Copies of this information may be obtained free of charge from: The Director, CCDC, 12 Union Road, Cambridge, CB2 1EZ, UK. Fax: +44(1223)336-033, e-mail:deposit@ccdc.cam.ac.uk, or www: www.ccdc.cam.ac.uk.

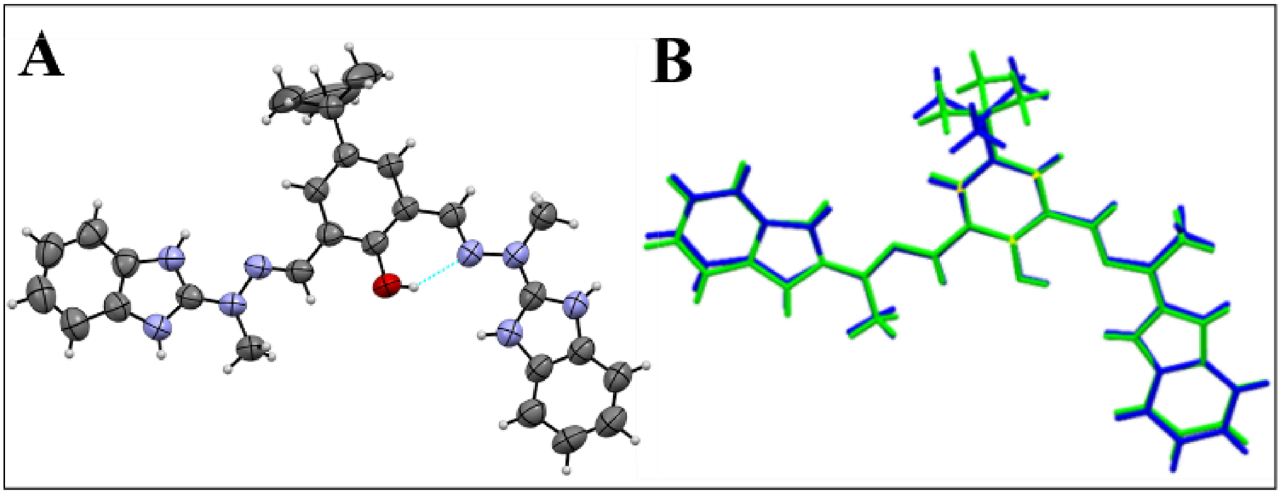

Figure S1. (top left) Anisotropic ellipsoid representation of the molecule A of a cation (H_5**L**^{1})$^{2+}$; ellipsoids are drawn at the 50% probability level, hydrogen atoms are shown as spheres of arbitrary radii, hydrogen bond is shown as thin blue line. (top right) Comparison of two symmetry-independent cations (fitting of the central rings).[1]

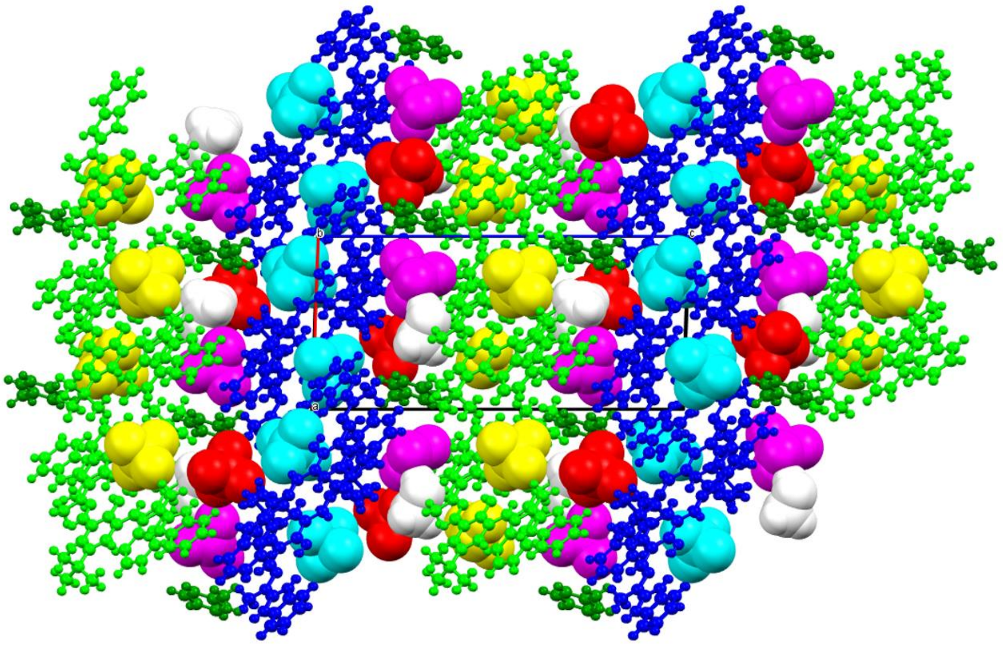

Figure S2. A crystal structure of $[H_5\mathbf{L}^1](ClO_4)_2$ as seen along b-direction. Anions and solvent molecules are shown in van der Waals spheres representation in order to visualize their filling-structure role.

Table S1. Relevant geometric parameters (Å, °).A, B, C, D, E – mean planes of subsequent planar fragments: A: N1 – N9; B: C2, N10, N12, C13, C14; C: C14, C15, C17, C18, C19, C24; D: C17, C25, N26, N27, C29; E: C30 – N37. The three (for c.n. 6) or two (c.n. 5) largest angles around metal ion are listed.

	$[H_5\mathbf{L}^1]^{2+}$	**1**	**2**	**3**	**4**	**5**	**6**	**7**	**8**	**9**	**10**	**11**
C-N	1.328<br>1.343<br>1.344<br>1.338	1.364<br>1.383	1.355<br>1.344<br>1.363<br>1.337	1.393<br>1.375	1.390<br>1.371	1.371<br>1.356<br>1.368<br>1.363	1.355<br>1.344<br>1.363<br>1.337	1.336<br>1.322	1.349<br>1.353<br>1.380<br>1.359	1.348(6)<br>1.289(6)	1.346<br>1.360<br>1.370<br>1.340	1.362<br>1.335
N-N	1.389<br>1.392<br>1.397<br>1.396	1.376<br>1.393	1.394<br>1.387<br>1.390<br>1.389	1.374<br>1.383	1.369<br>1.388	1.393<br>1.375<br>1.368<br>1.371	1.394<br>1.387<br>1.390<br>1.389	1.400<br>1.395	1.393<br>1.382<br>1.388<br>1.387	1.407(5)<br>1.404(5)	1.415<br>1.387<br>1.383<br>1.382	1.382<br>1.394
C=N	1.266<br>1.286<br>1.281<br>1.288	1.309<br>1.275	1.287<br>1.293<br>1.298<br>1.287	1.289<br>1.292	1.280<br>1.289	1.305<br>1.274<br>1.304<br>1.288	1.287<br>1.293<br>1.298<br>1.287	1.278<br>1.265	1.301<br>1.257<br>1.290<br>1.320	1.296(6)<br>1.289(6)	1.310<br>1.279<br>1.259<br>1.286	1.282<br>1.281
C-O	1.347<br>1.352	1.328	1.340<br>1.342	1.324	1.320	1.314<br>1.336	1.340<br>1.342	1.312	1.373<br>1.354	1.322(5)<br>1.337(6)	1.318<br>1.320	1.333
C-N-C	108.6<br>110.3<br>109.0	**104.1**<br>108.4<br>***106.1***	**105.4**<br>106.8<br>107.6	**103.1**<br>102.7<br>105.9	**102.3**<br>102.1<br>106.5	**105.2**<br>107.3<br>107.6	**105.4**<br>106,8<br>107.6	**105.3**<br>106.4<br>**106.2**	**104.6**<br>105.1<br>105.2	107.1(4)<br>106.6(4)<br>105.9(4)	107.6<br>107.5<br>105.7	**105.2**<br>105.6<br>108.1

	108.0<br>109.1<br>108.7<br>109.0<br>109.1	*105.4*<br>***102.0***<br>*105.3*	108.2<br>**105.1**<br>106.5	**104.5**	**103.4**	**105.5**<br>**103.2**<br>107.0<br>108.2<br>103.3	108.2<br>**105.1**<br>106.5<br>108.0<br>108.5	109.3	**106.5**<br>**104.5**<br>106.3<br>107.9<br>**104.1**	105.3(4)	**106.5**<br>108.6<br>107.6<br>105.9<br>**106.4**	107.6
A/B	6.7<br>5.8	4.6	13.7<br>10.0	6.1	5.8	5.9<br>3.6	13.7<br>10.0	6.1	2.2<br>27.3		6.4<br>4.4	20.0
B/C	1.5<br>6.4	19.5	33.2<br>33.5	12.3	13.6	17.1<br>40.7	33.2<br>33.5	13.2	24.4<br>31.6		42.5<br>13.4	38.3
C/D	1.5<br>3.2	18.5	18.8<br>23.0	22.5	22.7	25.8<br>14.8	18.8<br>23.0	12.3	26.0<br>25.8		27.7<br>23.6	9.6
D/E	6.0<br>6.0	*4.3*<br>*23.1*	6.4<br>4.9	2.5	3.4	0.2<br>23.0	6.4<br>4.9	1.9	12.6<br>14.7		20.0<br>22.4	15.9
A/E	13.5<br>10.2	*33.6*<br>*12.2*	12.4<br>14.6	29.2	29.3	39.8<br>4.8	12.4<br>14.6	2.0	14.8<br>11.1		14.0<br>14.6	46.0
M-O16		2.248(7)<br>2.257(6)	2.111(3)<br>2.164(3)	2.239(3)<br>2.262(4)	2.131(2)<br>2.159(2)	1,989(5)<br>2.021(5)<br>2.084(5)<br>2.178(5)	2.027(3)<br>2.148(3)<br>2.020(3)<br>2.112(3)	1.899(5)<br>1.84(1)	2.115(6)<br>2.368(6)<br>2.071(6)<br>2.324(6)	1.897(3)<br>1.864(3)	1.869(4)<br>1.867(5)	2.037(5)
M-N		2.26(1)<br>*2.27(9)*<br>*2.36(1)*<br>2.41(8)	2.190(4)<br>2.202(4)<br>2.363(5)<br>2.364(4)	2.172(4)<br>2.262(4)<br>2.453(4)<br>2.463(4)	2.169(3)<br>2.233(3)<br>2.366(3)<br>2.366(3)	2.023(6)<br>2.238(6)<br>2.095(7)<br>2.237(6)<br>2.253(6)	2.020(4)<br>2.048(4)<br>2.054(4)<br>2.036(4)<br>2.043(4)<br>2.062(4)	2.038(7)<br>2.167(6)<br>2.15(1)<br>2.26(1)	2.052(8)<br>2.047(8)<br>2.058(7)<br>2.050(7)<br>2.088(8)<br>2.052(8)<br>2.066(7)	1.932(4)<br>1.906(4)<br>1.903(4)<br>1.917(4)	1.919(6)<br>1.923(6)<br>1.933(5)<br>1.959(6)	2.085(6)<br>2.088(5)
M-O(water)						2.010(6)			2.120(7)<br>2.081(7)			
M-O(methanol)							2.148(3)<br>1.118(3)		2.150(7)			
M-OH									2.071(6)<br>2.088(6)<br>1.995(5)<br>2.086(6)<br>2.019(6)			
M-N(CH3CN)									2.094(9)			
M-Cl								2.204(3)				

								2.277(3)<br><br>2.22(1)				
angles		161.4(2)<br>141.5(5)<br>126.3(5)	161.8(1)<br>140.9(1)<br>136.5(1)	163.1(1)<br>133.3(1)<br>133.3(1)	158.6(1)<br>13.7(1)<br>135.8(1)	150.7(3)<br>143.1(3)<br><br>159.6(2)<br>147.2(2)<br>142.2(2)	171.9(2)<br>169.6(2)<br>166.8(1)<br><br>173.0(2)<br>168.1(1)<br>167.9(1)	151.2(2)<br>139.9(2)<br><br>173.2(6)<br>140.1(5)	172.1(2)<br>168.4(3)<br>167.2(3)<br><br>173.5(2)<br>166.4(3)<br>162.1(3)<br><br>172.0(3)<br>169.5(2)<br>165.1(3)<br><br>172.4(3)<br>170.8(3)<br>163.2(3)		176.4(3)<br>175.8(2)<br>175.4(2)	174.8(2)<br>162.7(2)<br>162.7(2)

Table S2. Hydrogen bond data (Å, °)

D	H	A	D-H	H··A	D···A	D-H···A
**$(H_5L^1)^{2+}$**						
N1A	H1A	O4C	0.86	2.02	2.87498)	170
N3A	H3A	$O2D^{i}$	0.86	2.07	2.852(8)	151
O16A	H16A	N26A	0.82	2.00	2.722(6)	146
N30A	H30A	01G	0.86	1.98	2.838(8)	176
N37A	H37A	O4D	0.86	1.97	2.754(7)	152
N1B	H1B	O4F	0.86	1.98	2.765(7)	152
N3B	H3B	$01C^{ii}$	0.86	2.10	2.883(8)	152
016B	H16B	N12B	0.82	1.99	2.697(6)	144
N30B	H30B	$O1F^{iii}$	0.86	2.11	2.853(8)	144
N37B	H37B	O3E	0.86	2.02	2.869(8)	169
O1G	H1G	O2E	0.82	2.06	2.873(10)	171
**1**						
N3A	H3A	$O1^{XI}$	0.86	2.06	2.889(16)	161
N37A	H37A	$O1^{xii}$	0.86	2.08	2.912(17)	164
N37B	H37B	$O1^{xii}$	0.86	1.92	2.744(16)	159
**2**						
N3A	H3A	$O2C^{xiii}$	0.88	2.26	3.000(7)	142

N3A	H3A	O3C[xiii]	0.88	2.23	3.054(6)	155
N3B	H3B	O3C[xiv]	0.88	1.99	2.856)6=	169
			**3**			
N30A	H30A	O1B[xv]	0.88	1.83	2.685(5)	164
O1B	H1B	N3A[xvi]	0.84	1.86	2.683(5)	165
			**4**			
N30A	H30A	O1B[xv]	0.86	1.86	2.703(3)	168
O1B	H1B	N3A[xvi]	0.82	1.88	2.689(3)	169
			**5**			
N3A	H3A	O3E[iv]	0.88	1.98	2.857(9)	177
N30A	H30A	O1D[i]	0.88	1.94	2.742(14)	151
N3B	H3B	O1E[v]	0.88	1.99	2.846(9)	165
O1G	H1G	N30B	0.82	2.01	2.787(19)	156
O1W	H1W1	O1F	0.82	1.86	2.660(8)	165
O1W	H1W2	O2E	0.82	1.95	2.774(9)	175
			**6**			
N3A	H3A	O3J[vi]	0.88	2.09	2.895(5)	152
N30A	H30A	O1L	0.88	1.91	2.711(6)	151
N37A	H37A	O1K	0.88	1.99	2.809(6)	155
N3B	H3B	O2H	0.88	1.98	2.838(6)	166
N30B	H30B	O1N	0.88	1.83	2.702(6)	169
N37B	H37B	O2J[vi]	0.88	2.10	2.885(7)	148
O1E	H1E	O2G[vii]	0.88	1.97	2.708(6)	140
O1E	H1E	O2G'[vii]	0.88	2.01	2.884(7)	175
O1F	H1F	O1M	0.82	1.93	2.722(5)	164
O1K	H1K	O2H	1.00	1.89	2.870	165
O1L	H1L	O4J	1.00	1.74	2.740(6)	175
O1M	H1M	O1I	0.84	2.01	2.842(5)	171
O1N	H1N	O3H[viii]	1.00	2.25	3.003	131
O1N	H1N	O4H[ix]	1.00	2.36	3.014(9)	123
			**7**			
N3A	H3A	Cl2B[xxiii]	0.88	2.50	3.378(7)	174
N37A	H37A	N1D[xx]	0.88	2.02	2.893(10)	171
			**8**			
N3A	N3A	N3A	N3A	N3A	N3A	N3A
N30A	N30A	N30A	N30A	N30A	N30A	N30A
N3B	N3B	N3B	N3B	N3B	N3B	N3B
N30B	N30B	N30B	N30B	N30B	N30B	N30B
O1D	O1D	O1D	O1D	O1D	O1D	O1D
O1D	O1D	O1D	O1D	O1D	O1D	O1D

O1E	O1E	O1E	O1E	O1E	O1E	O1E
O1F	O1F	O1F	O1F	O1F	O1F	O1F
O1K	O1K	O1K	O1K	O1K	O1K	O1K
O1M	O1M	O1M	O1M	O1M	O1M	O1M
O1N	O1N	O1N	O1N	O1N	O1N	O1N
O1N	O1N	O1N	O1N	O1N	O1N	O1N
**9**						
N00A	N00A	N00A	N00A	N00A	N00A	N00A
**10**						
N1B	N1B	N1B	N1B	N1B	N1B	N1B
N3B	N3B	N3B	N3B	N3B	N3B	N3B
N30B	N30B	N30B	N30B	N30B	N30B	N30B
O1F	O1F	O1F	O1F	O1F	O1F	O1F
O1W	O1W	O1W	O1W	O1W	O1W	O1W
O2W	O2W	O2W	O2W	O2W	O2W	O2W
O2W	O2W	O2W	O2W	O2W	O2W	O2W
O2W	O2W	O2W	O2W	O2W	O2W	O2W
O3W	O3W	O3W	O3W	O3W	O3W	O3W
**11**						
N37A	H37A	O1D	0.88	2.19	2.945(8)	143
N30A	H30A	O1C^{xviii}	0.88	1.82	2.692(8)	173
O1C	H1C	O16A	0.84	1.75	2.592(8)	178

IV. ^{1}H NMR solution studies

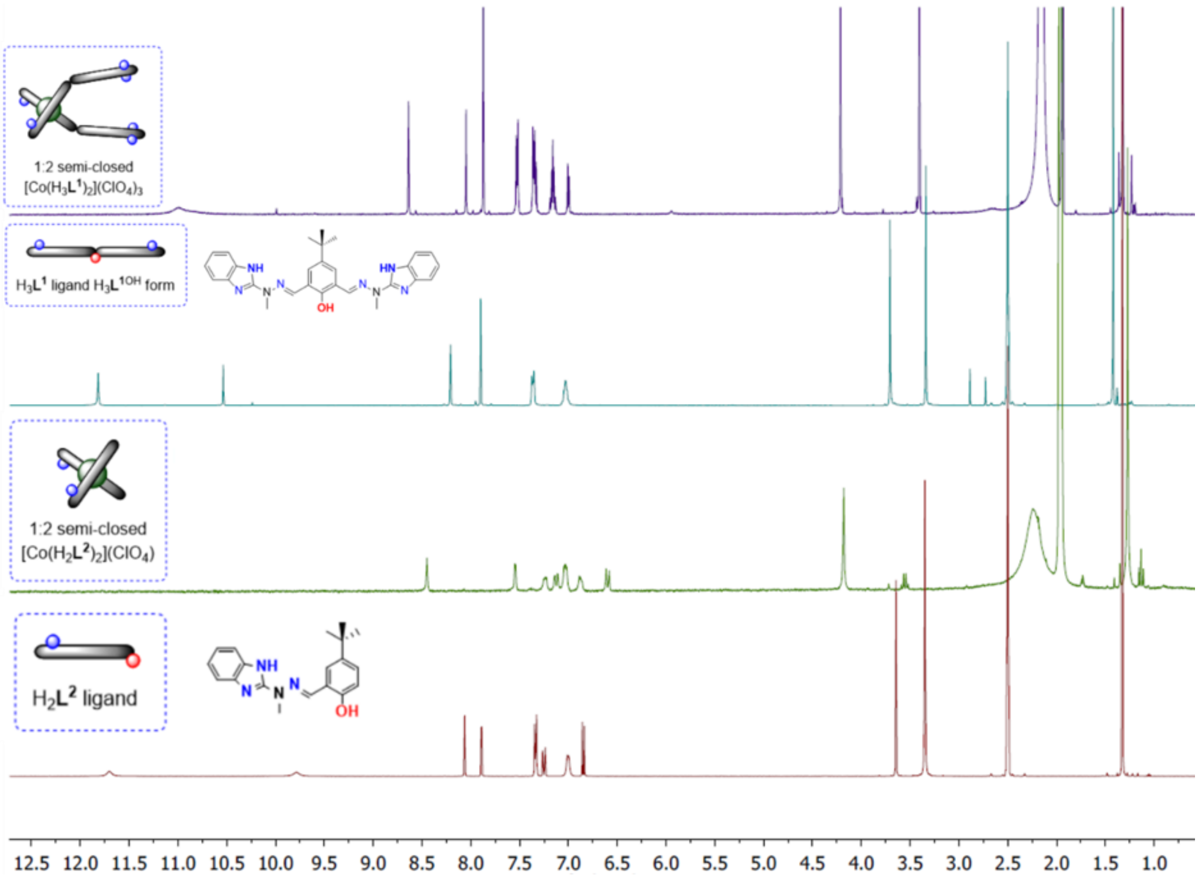

Figure S3. Comparison of $H_3\mathbf{L}^1$ and $H_2\mathbf{L}^2$ ligands with their semi-closed 1:2 complexes with Co^{III} ions in the presence of perchlorate. Please note that solvent for ligands is d^6-DMSO, whereas for complexes it is CD_3CN.

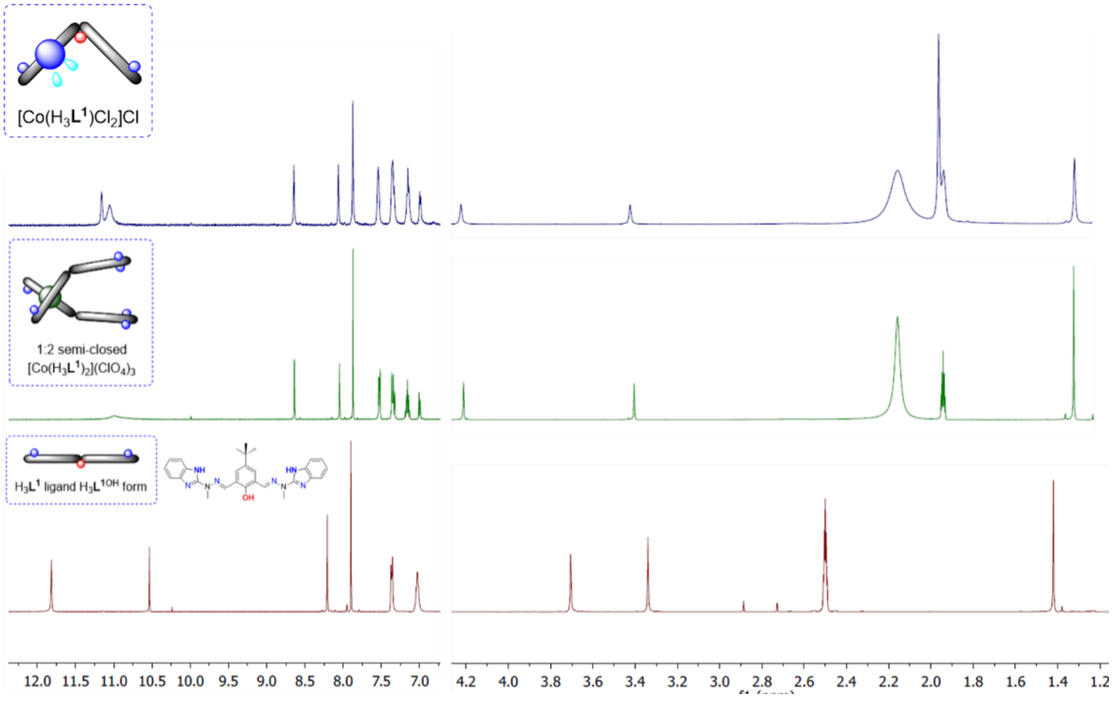

Figure S4. Comparison of $H_3\mathbf{L}^1$ ligand with semi-closed 1:2 and 1:1 complexes with Co^{III} ions in the presence of perchlorate and chloride anions. Please note that solvent for ligand is d^6-DMSO, whereas for complexes it is CD_3CN.

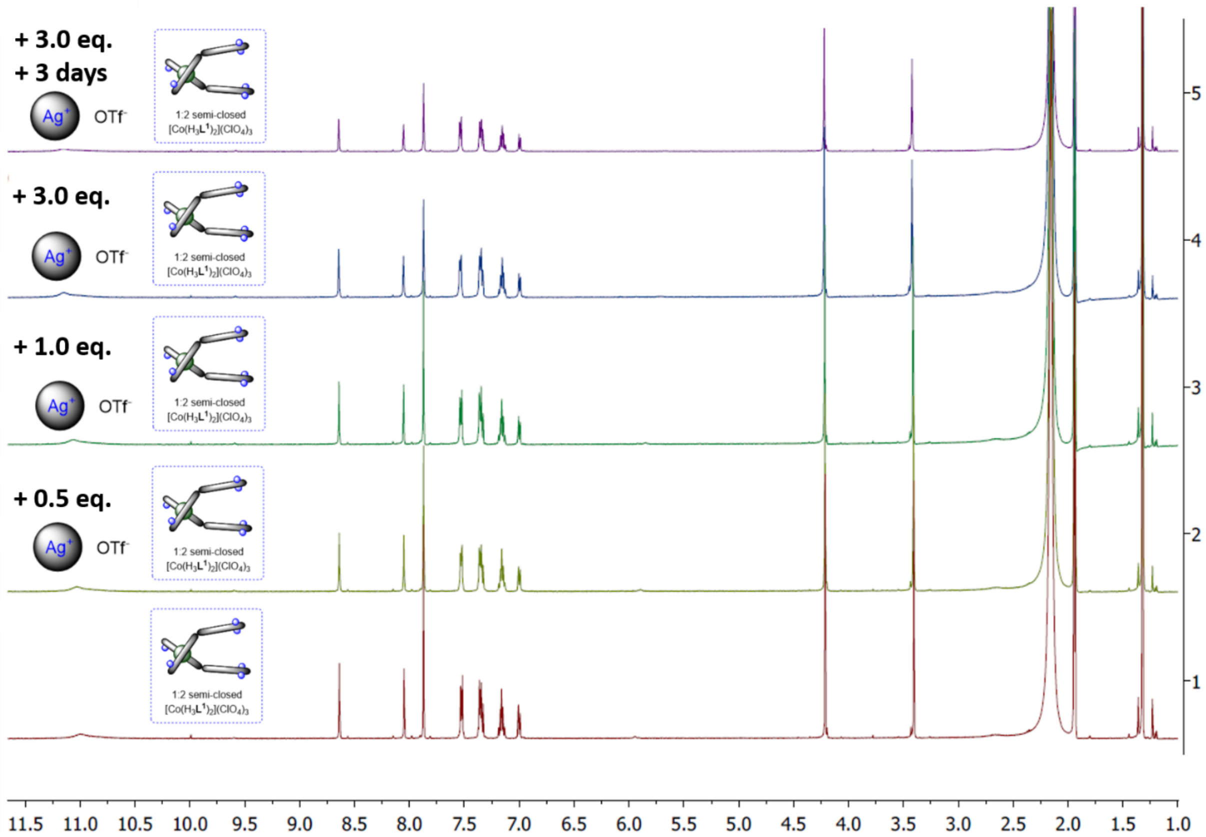

Figure S5. Titration of $[Co(H_3\mathbf{L}^{1\text{-}NH})_2](ClO_4)_3$ (**10**) with AgOTf in CD_3CN.

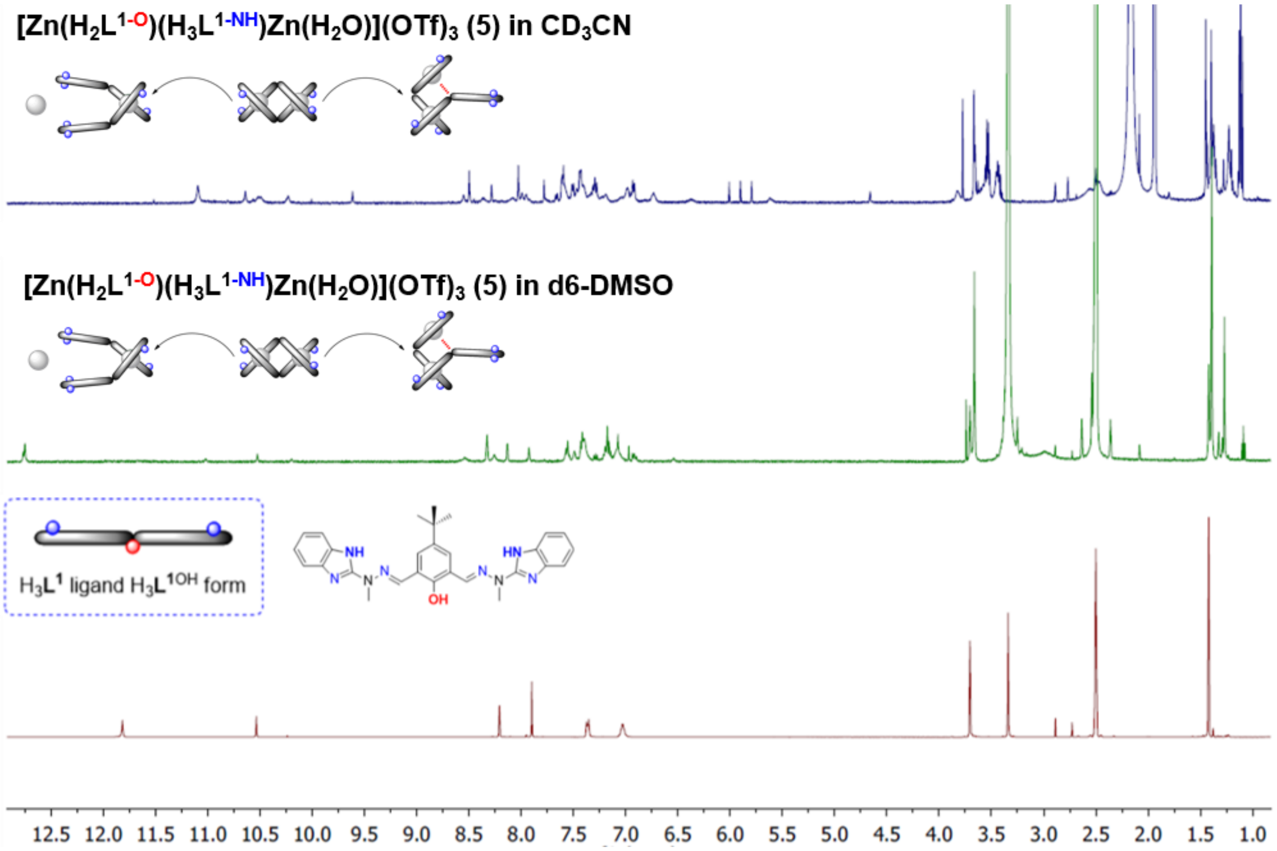

Figure S6. Comparison of $H_3\mathbf{L}^1$ ligand with semi-closed 2:2 $Zn(OTf)_2$ complex **5** in deuterated acetonitrile and DMSO.

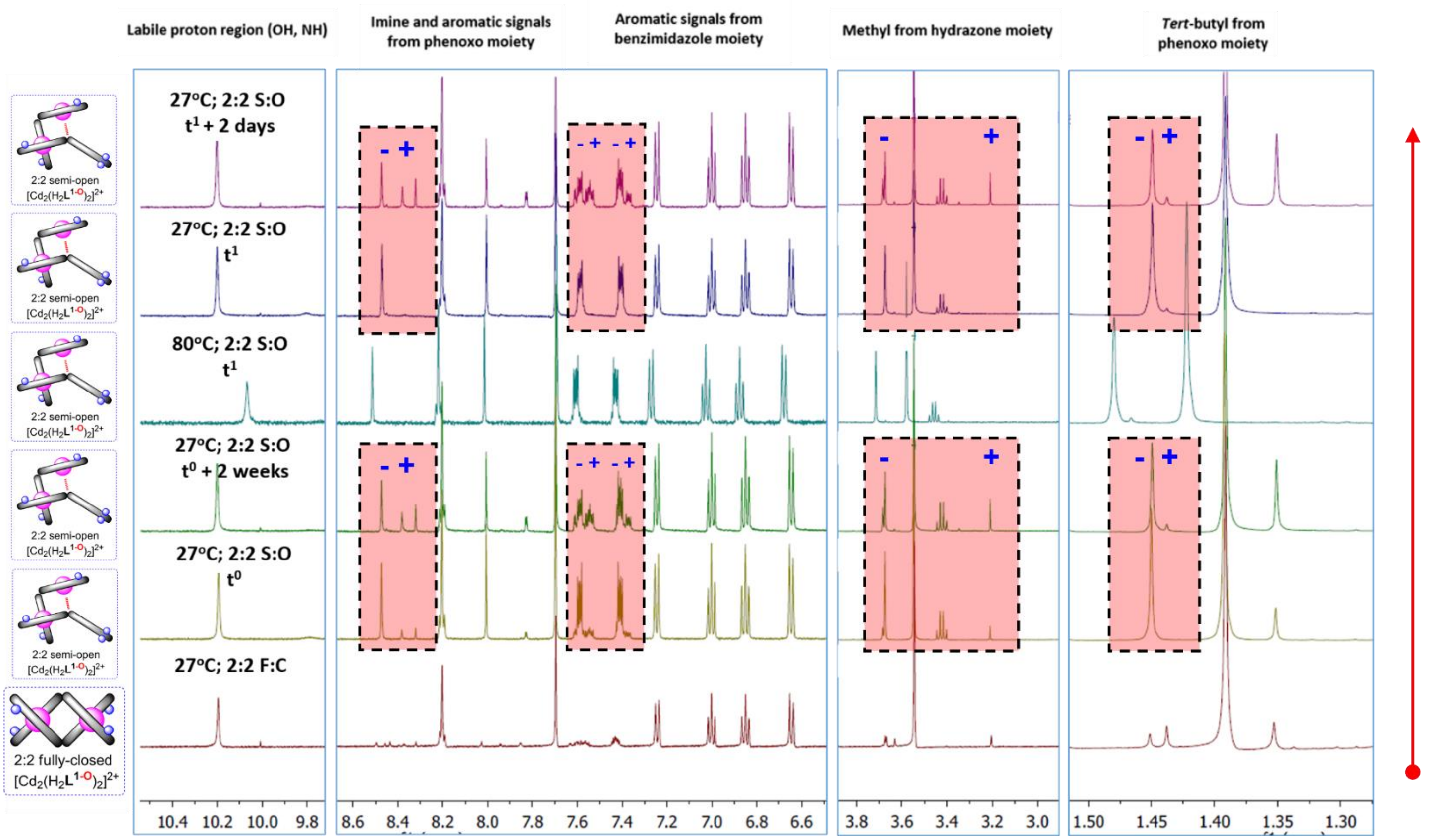

Figure S7. Time-and temperature dependent changes of the $[Cd_2(H_2\mathbf{L}^{\mathbf{1\text{-}O}})_2](ClO_4)_2$ (**1**) complex in CD_3CN solvent; F-C structure is obtained only upon immediate dissolution of crystals of **1** and measurement; t^0 is the moment when strong signal of imine is observed, followed other changes in the spectrum marked with blue marks, t^1 denotes heating to 80°C, which 'resets' the equilibrium present in solution.

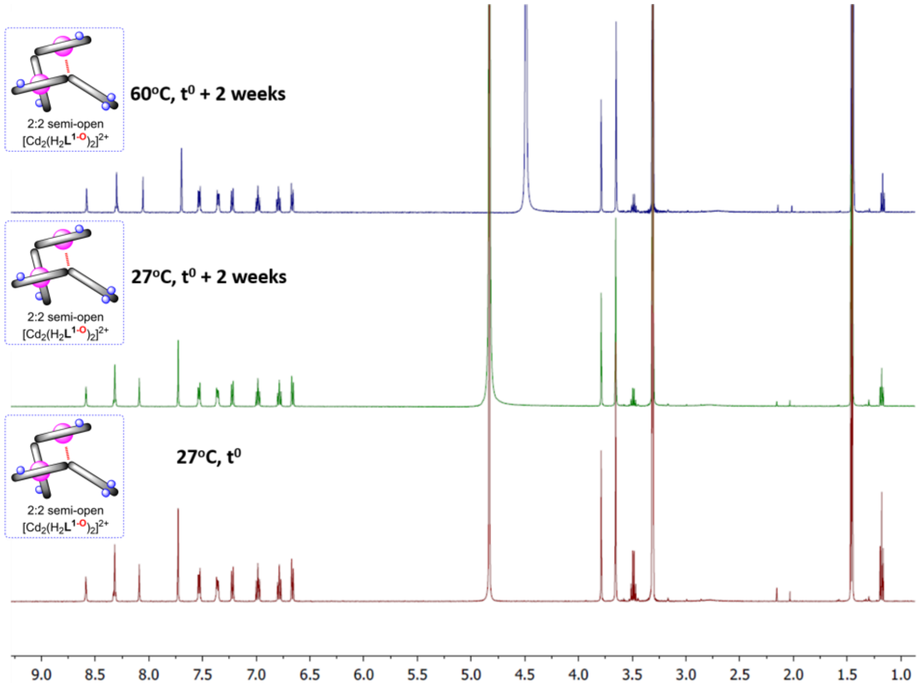

Figure S8. Time-and temperature dependent changes of the $[Cd_2(H_2\mathbf{L}^{\mathbf{1\text{-}O}})_2](ClO_4)_2$; (**1**) complex in CD_3OD solvent; t^0 is the moment when crystals of (**1**) are dissolved in solvent and the spectrum measured.

V. Absorption and emission spectra

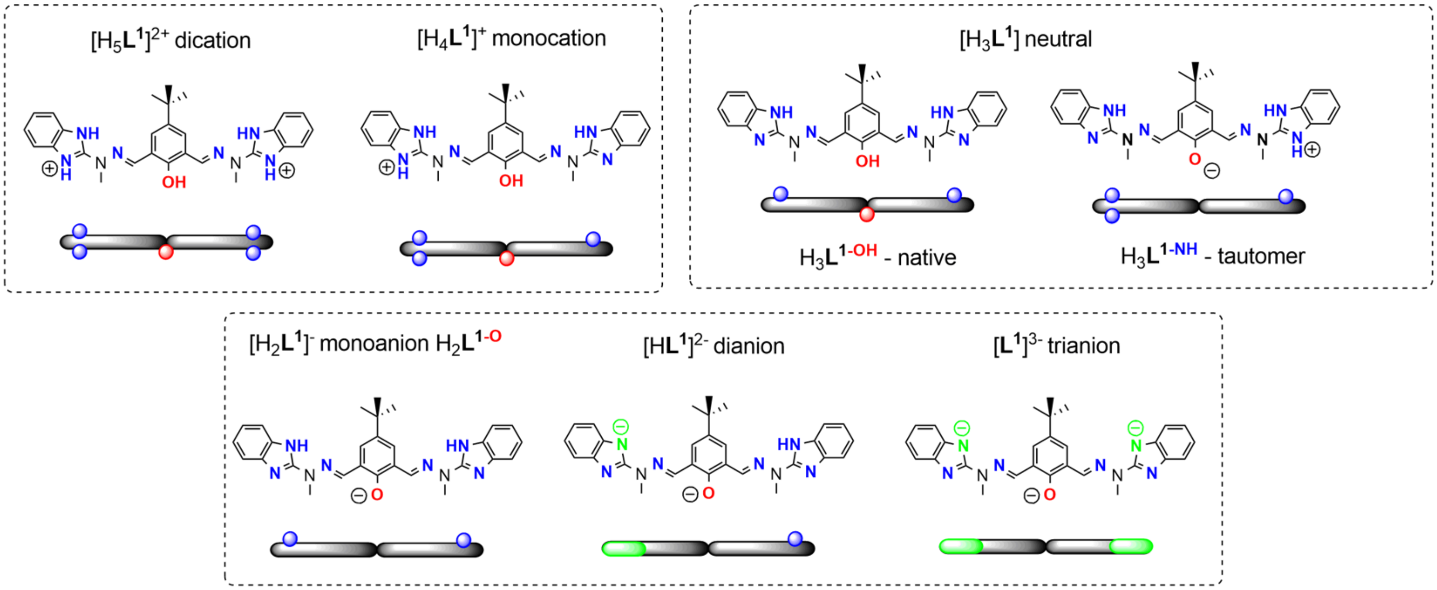

Scheme S3. Possible form of H_3**L**[1] ligand in a function of their protonation and deprotonation.

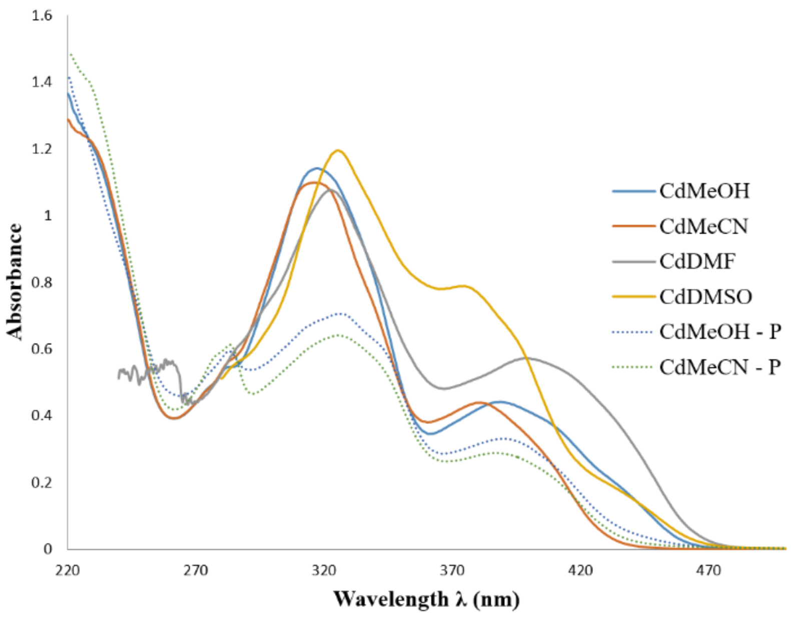

Figure S9. Absorption spectra of compound **1** in different solvents. P- measurement after 5 days of dissolution. All absorption spectra were recorded using solutions 2 x 10^{-5} M with respect to the metal ions. Extinction coefficients were presented in Table S3.

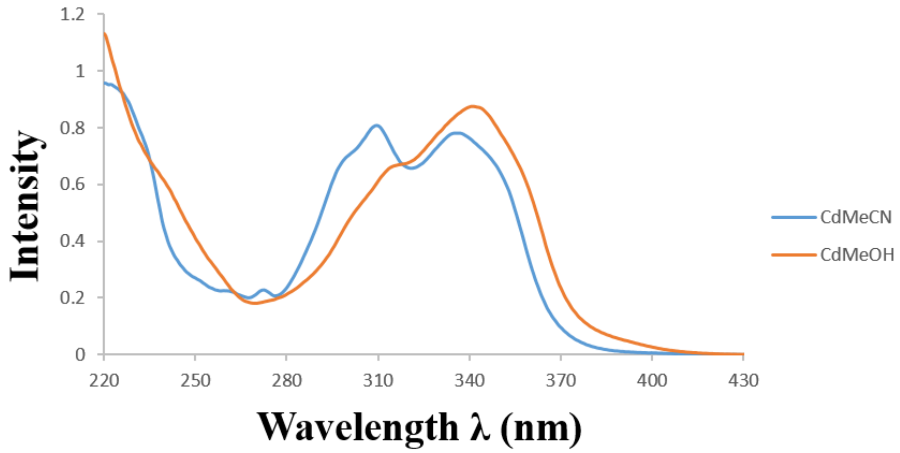

Figure S10. Absorption spectra of compound **13**. All absorption spectra were recorded using solutions 2 x 10^{-5} M with respect to the metal ions. Extinction coefficients are presented in Table S3.

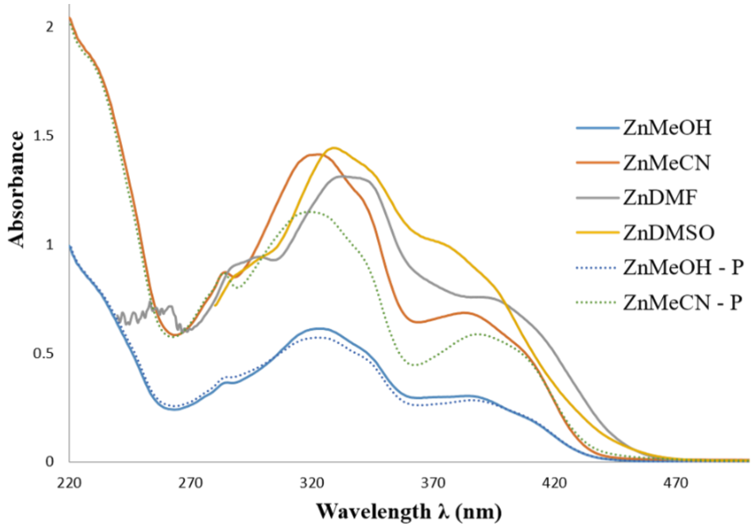

Figure S11. Absorption spectra of compound **5** in different solvents. P- measurement after 5 days of dissolution. All absorption spectra were recorded using solutions 2 x 10^{-5} M with respect to the metal ions. Extinction coefficients are presented in Table S3.

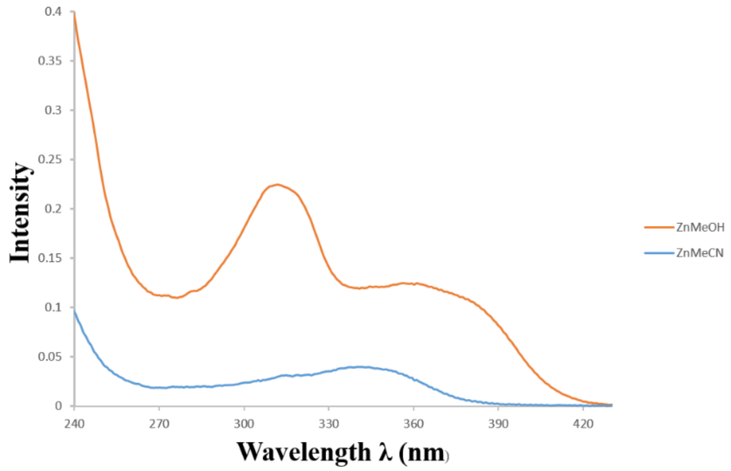

Figure S12. Absorption spectra of compound **12** in methanol and acetonitrile. All absorption spectra were recorded using solutions 2 x 10^{-5} M with respect to the metal ions. Extinction coefficients are presented in Table S3.

Table S3. Molar extinction coefficients for compounds **1**, **5**, **12**, **13**.

Compound	Solvent	Absorbance	Absorbance maximum (nm)	Molar extinction coefficient (cm^{-1} M^{-1})
**1**	MeOH	1.146	317	57 300
**1**	MeCN	1.106	316	55 300
**1**	DMF	1.081	323	54 050
**1**	DMSO	1.201	325	60 050
**1 – P**	MeOH	0.712	326	35 600
**1 – P**	MeCN	0.649	326	32 450
**5**	MeOH	0.621	322	31 050
**5**	MeCN	1.415	321	70 750
**5**	DMF	1.318	333	65 900
**5**	DMSO	1.444	329	72 200
**5 – P**	MeOH	0.578	322	28 900
**5 – P**	MeCN	1.152	321	57 600
**12**	MeOH	0.223	314	11 150
**12**	MeCN	0.038	346	1 900
**13**	MeOH	0.875	341	43 750
**13**	MeCN	0.808	310	40 400

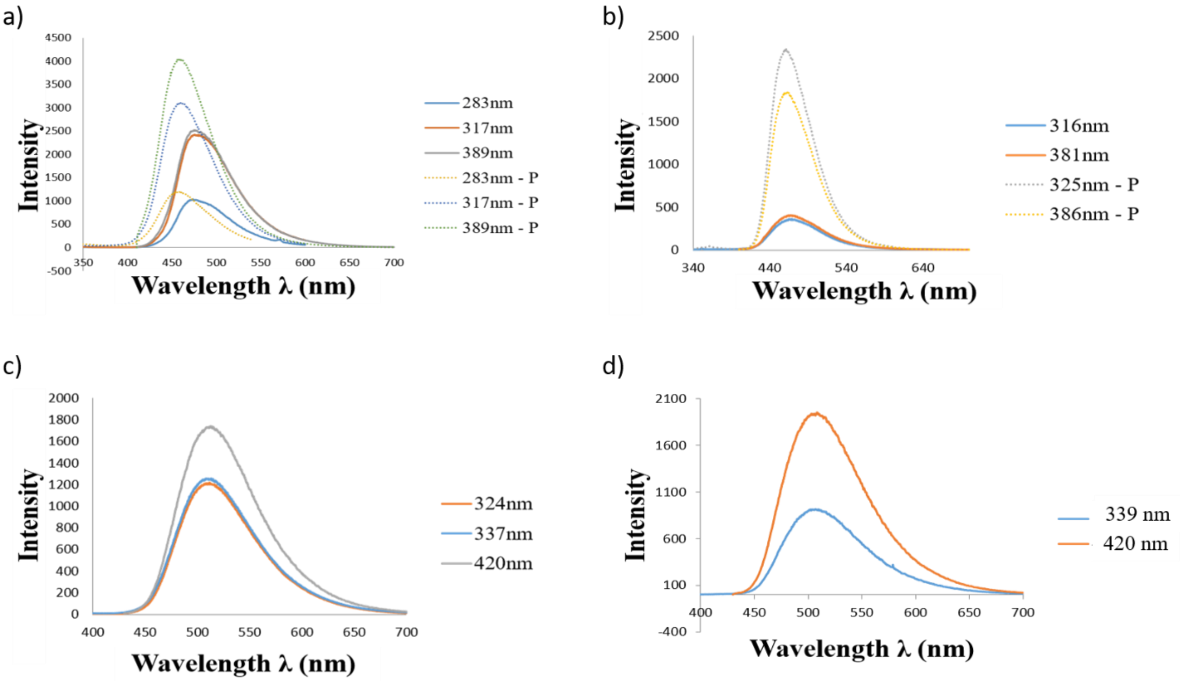

Figure S13. Emission spectra of compound **1** in: a) methanol, b) acetonitrile, c) dimethylformamide, d) dimethylsulfoxide. P- measurement after 5 days of dissolution; wavelength values denote the excitation wavelengths.

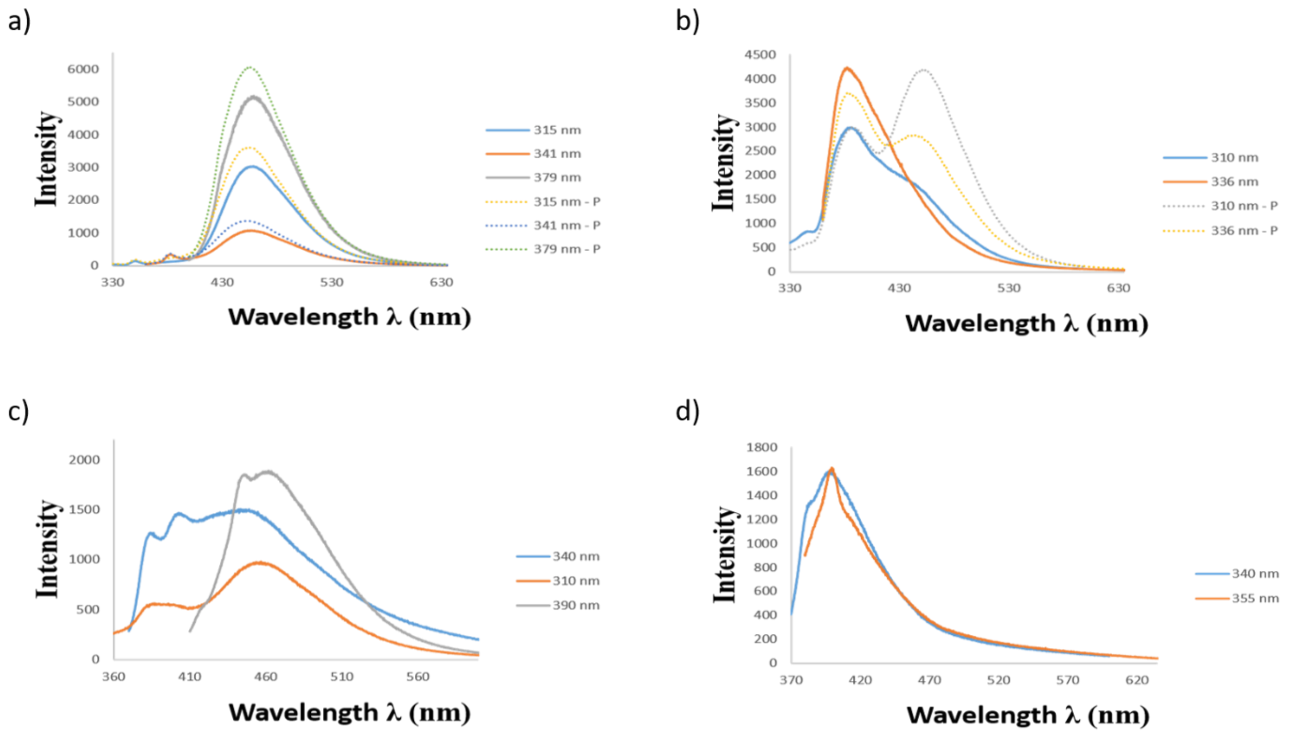

Figure S14. Emission spectra of compound **13** in: a) methanol, b) acetonitrile, c) dimethylformamide, d) dimethylsulfoxide. P- measurement after 5 days of dissolution; wavelength values denote the excitation wavelengths.

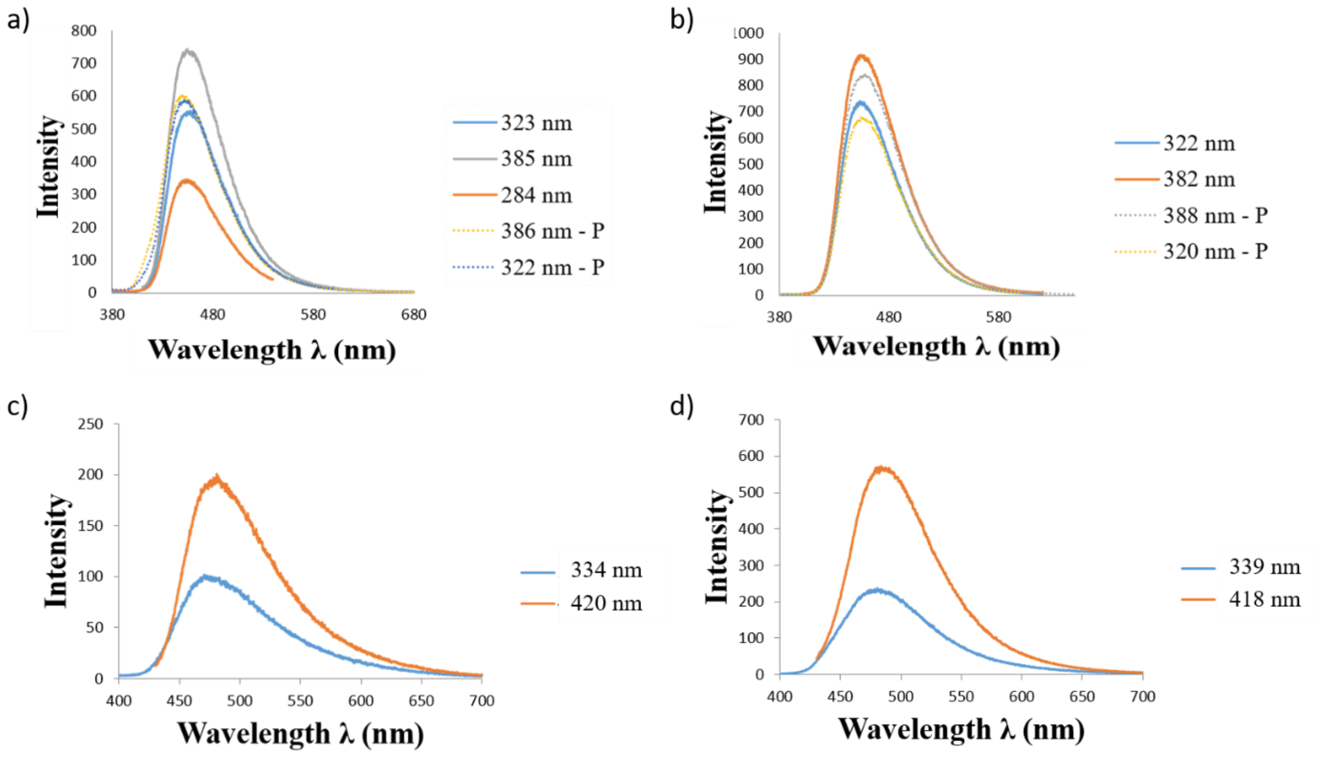

Figure S15. Emission spectra of compound **5** in: a) methanol, b) acetonitrile, c) dimethylformamide, d) dimethylsulfoxide. P- measurement after 5 days of dissolution; wavelength values denote the excitation wavelengths.

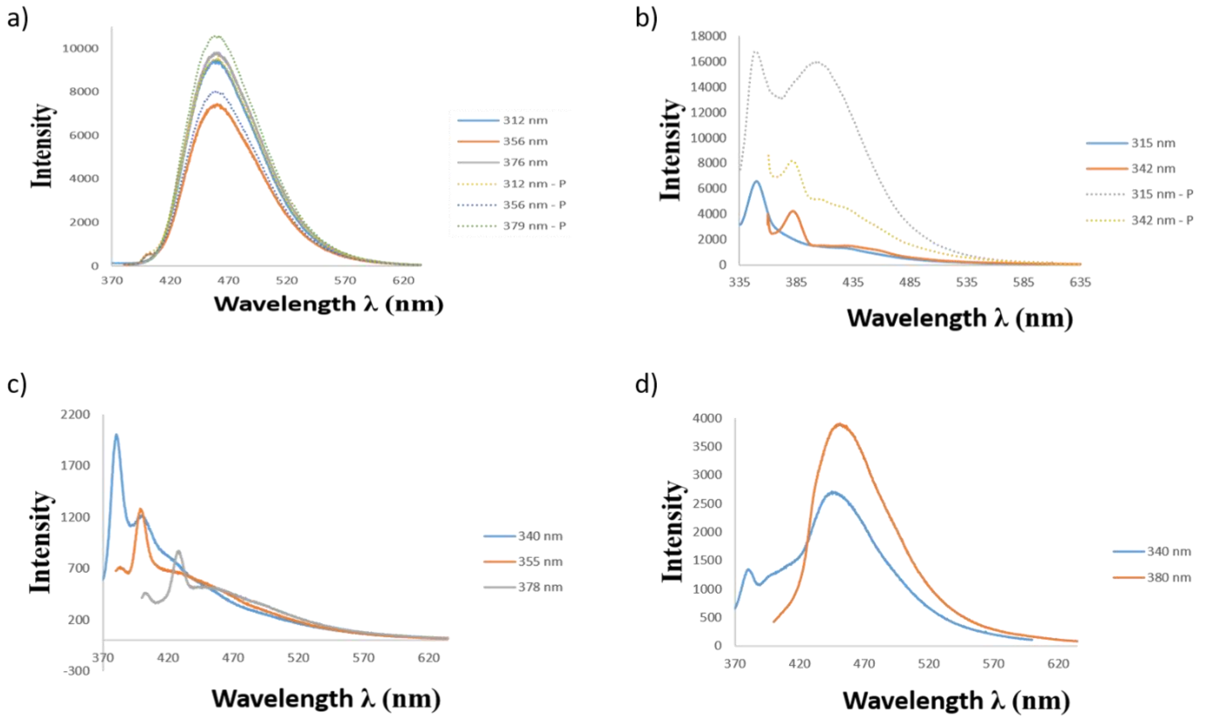

Figure S16. Emission spectra of compound **12** in: a) methanol, b) acetonitrile, c) dimethylformamide, d) dimethylsulfoxide. P- measurement after 5 days of dissolution; wavelength values denote the excitation wavelengths.

Table S4. Collected results of quantum yields for complexes **1** and **5** with schematic representation.

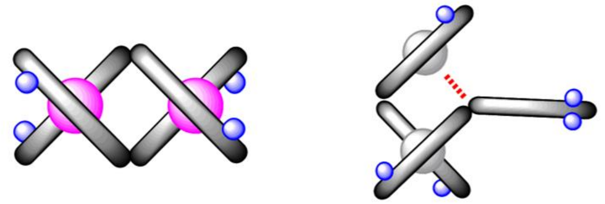

$[Cd_2(H_2\mathbf{L}^{1\text{-}O})](ClO_4)$ (**1**)

$[Zn(H_2\mathbf{L}^{1\text{-}O})(H_3\mathbf{L}^{1\text{-}NH})Zn(H_2O)](OTf)_3$ (**5**)

Compound	Solvent	Quantum yield [%]
FLUORESCEIN		
1	MeOH	1.12
5	MeOH	0.66
1	MeCN	0.46
5	MeCN	0.55
QUININE SULFATE		
1	DMF	2.74
5	DMF	0.96
1	DMSO	0.73
5	DMSO	3.15

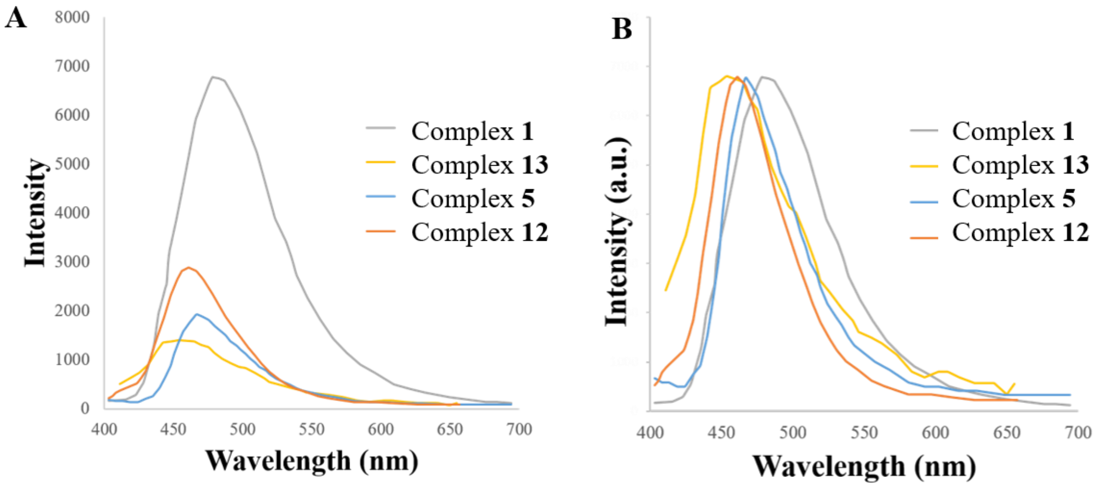

Figure S17. (**A**) Solid state emission spectra for compounds **1**, **5**, **12**, **13**. (**B**) Normalized solid state emission spectra for compounds **1**, **5**, **12**, **13**.

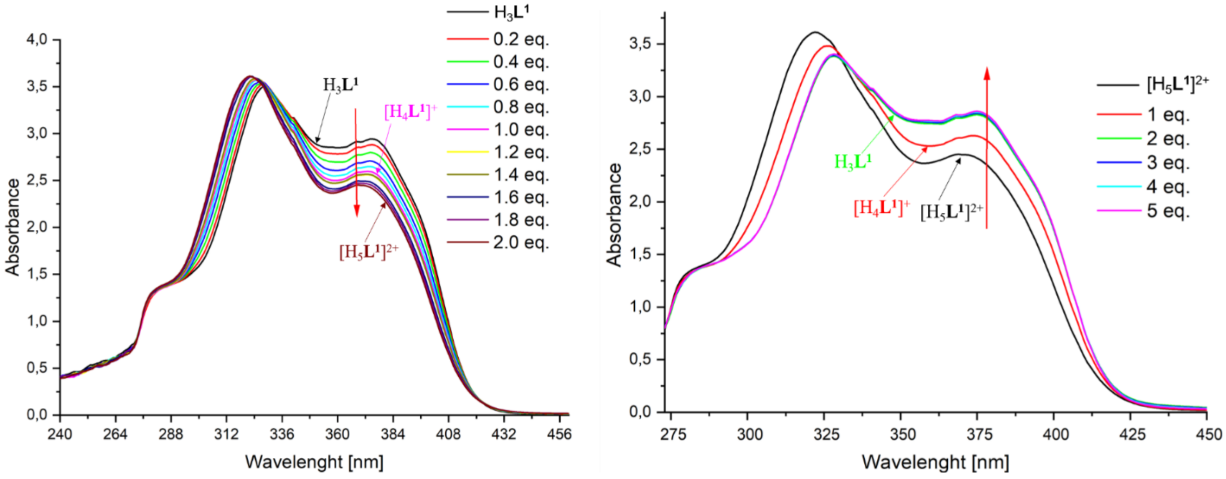

Figure S18. Absorption spectra of titration of ligand $H_3\mathbf{L^1}$ (up) with acid - HCl and (bottom) with base – triethylamine (c = 2 · 10^{-5} M in methanol).

Spectra show two bands, whose absorption maxima are at λ = 328 nm and λ = 378 nm. During titration with acid, the absorption intensity decreases and a slight hypochromic shift (blueshift) is present. As the first acid equivalent is added, a proportional decrease in absorbance were observed. This indicates the formation of another protonated form of the ligand, respectively $H_3\mathbf{L^1} \rightarrow [H_4\mathbf{L^1}]^+$ after the addition of 1 equivalent, and $[H_4\mathbf{L^1}]^+ \rightarrow [H_5\mathbf{L^1}]^{2+}$ after addition of second equivalent of acid, no further changes were observed at UV spectra, which means that we were able to protonate the ligand to form $[H_5\mathbf{L^1}]^{2+}$ (with two additional protons on N atom from imidazole ring – compare with X-ray in Section 2.1.1.). The base titration of the protonated form of the ligand $[H_5\mathbf{L^1}]^{2+}$ illustrates its deprotonation with the addition of subsequent base equivalents and the transition, respectively, from the form of $[H_5\mathbf{L^1}]^{2+} \rightarrow [H_4\mathbf{L^1}]^+ \rightarrow H_3\mathbf{L^1}$. Further addition of triethylamine did not cause any change in the in absorbance intensity, indicating that triethylamine is too weak a base to yield more deprotonated forms of the ligand ($[H_2\mathbf{L^1}]^-$, $[H\mathbf{L^1}]^{2-}$, $[\mathbf{L^1}]^{3-}$).

VI. Literature

1 Gorczyński, A., Kubicki, M., Szymkowiak, K., Łuczak, T. & Patroniak, V. Utilization of a new gold/Schiff-base iron(iii) complex composite as a highly sensitive voltammetric sensor for determination of epinephrine in the presence of ascorbic acid. *RSC Adv.* **6**, 101888-101899, doi:10.1039/C6RA22028B (2016).

2 Loos, M., Gerber, C., Corona, F., Hollender, J. & Singer, H. Accelerated isotope fine structure calculation using pruned transition trees. *Anal. Chem.* **87**, 5738-5744 (2015).

3 Chilton, N. F., Anderson, R. P., Turner, L. D., Soncini, A. & Murray, K. S. PHI: A powerful new program for the analysis of anisotropic monomeric and exchange-coupled polynuclear d-and f-block complexes. *J. Comput. Chem.* **34**, 1164-1175 (2013).

4 Technologies, A. (Agilent Technologies Ltd, 2011).

5 Altomare, A., Cascarano, G., Giacovazzo, C. & Guagliardi, A. Completion and refinement of crystal structures with SIR92. *J. Appl. Crystallogr.* **26**, 343-350 (1993).

6 Sheldrick, G. M. Crystal structure refinement with SHELXL. *Acta Crystallogr. C. Struct. Chem.* **71**, 3-8 (2015).

7 Spek, A. L. PLATON SQUEEZE: a tool for the calculation of the disordered solvent contribution to the calculated structure factors. *Acta Crystallogr. C. Struct. Chem.* **71**, 9-18 (2015).
