# Supplementary material for: Unexpected structural complexity of d-block metallosupramolecular architectures within the benzimidazole-phenoxo ligand scaffold for crystal engineering aspects
Source: Sci Rep. 2023 Oct 23;13:18055. doi: 10.1038/s41598-023-45109-z (PMC10593740; doi:10.1038/s41598-023-45109-z)
Supplement: Supplementary file 2 — Supplementary Information 2. [file 41598_2023_45109_MOESM2_ESM.docx]

- Multidentate ligand with benzimidazole and phenolate subunits effectively coordinates variety of d-block metal ions
- Unexpected structural diversity can be tuned via metal ion, counterion, pH and solvent
- X-ray studies show the importance of hydrogen bonding and benzimidazole moiety
- Subtle structural changes result in different magnetic exchange interactions
- Photoluminescence studies are affected by the solution equilibria
